# Circulating biomarkers associated with pediatric sickle cell disease

**DOI:** 10.3389/fmolb.2024.1481441

**Published:** 2024-12-19

**Authors:** Cecilia Elorm Lekpor, Felix Abekah Botchway, Adel Driss, Alaijah Bashi, Afua D. Abrahams, Kwadwo Asamoah Kusi, Godfred Futagbi, Ernest Alema-Mensah, William Agbozo, Wesley Solomon, Adriana Harbuzariu, Andrew A. Adjei, Jonathan K. Stiles

**Affiliations:** ^1^ Department of Microbiology, Biochemistry and Immunology, Morehouse School of Medicine, Atlanta, GA, United States; ^2^ Department of Pathology, Korle-Bu Teaching Hospital, University of Ghana Medical School, Accra, Ghana; ^3^ Department of Animal Biology and Conservation Sciences, University of Ghana, Accra, Ghana; ^4^ Department of Medical Laboratory Science, Accra Technical University, Accra, Ghana; ^5^ Department of Physiology, Morehouse School of Medicine, Atlanta, GA, United States; ^6^ Department of Immunology, Noguchi Memorial Institute for Medical Research, University of Ghana, Accra, Ghana; ^7^ Community Health and Preventive Medicine, Morehouse School of Medicine, Atlanta, GA, United States; ^8^ Emory Stem Cell Core, Emory University, Atlanta, GA, United States

**Keywords:** global health, inflammation biomarkers, hemoglobinopathies, pediatric hematology, oxidative stress

## Abstract

**Introduction:**

Sickle cell disease (SCD) is a genetic blood disorder caused by a mutation in the HBB gene, which encodes the beta-globin subunit of hemoglobin. This mutation leads to the production of abnormal hemoglobin S (HbS), causing red blood cells to deform into a sickle shape. These deformed cells can block blood flow, leading to complications like chronic hemolysis, anemia, severe pain episodes, and organ damage. SCD genotypes include HbSS, HbSC (HbC is an abnormal variant of hemoglobin), and HbS/β-thalassemia. Sickle cell trait (SCT), HbAS, represents the carrier state, while other hemoglobin variants include HbCC, HbAC, and the normal HbAA. Over 7.5 million people worldwide live with SCD, with a high mortality rate in sub-Saharan Africa, including Ghana. Despite its prevalence, SCD is underdiagnosed and poorly managed, especially in children. Characterized by intravascular hemolysis, SCD leads to oxidative stress, endothelial activation, and systemic inflammation. Identifying circulating blood biomarkers indicative of organ damage and systemic processes is vital for understanding SCD and improving patient management. However, research on biomarkers in pediatric SCD is limited and few have been identified and validated. This study explores specific circulating biomarkers in pediatric SCD in Ghana (West Africa), hypothesizing that inflammatory and neuronal injury markers in children with SCD could predict disease outcomes.

**Methods:**

Clinical data were collected from 377 children aged 3–8 years with various Hb genotypes, including SCD and SCT, at Korle-Bu Teaching Hospital in Accra, Ghana (2021–2022). A total of 80 age- and sex-matched subjects were identified. A cross-sectional study utilized a multiplexed immunoassay procedure to evaluate serum biomarkers, including cytokines, chemokines, vascular injury markers, systemic inflammation markers, cell-free heme scavengers, brain-derived neurotrophic factor (BDNF), and angiogenic factors.

**Results:**

Elevated levels of BDNF, Ang-2, CXCL10, CCL11, TNF-α, IL-6, IL-10, IL12p40, ICAM-1, VCAM-1, Tie-2, and VEGFA were observed in HbSS subjects, correlating with hemoglobin level, leukocyte, and erythrocyte counts. Heme scavengers like HO-1, hemopexin, and haptoglobin also correlated with these parameters. ROC and AUC analyses demonstrated the potential of these biomarkers in predicting SCD outcomes.

**Conclusion:**

These findings suggest that there are significant differences between biomarker expression among the different genotypes examined. We conclude that a predictive algorithm based on these biomarkers could be developed and validated through longitudinal assessment of within-genotype differences and correlation of the data with disease severity or outcomes. With such a tool one can enhance SCD management and improve patient outcomes. This approach may pave the way for personalized interventions and better clinical care for pediatric SCD patients.

## Introduction

Sickle cell disease (SCD) refers to a group of inherited disorders affecting red blood cells, driven by a specific mutation in the beta-globin gene. This mutation leads to the substitution of valine for glutamic acid at the sixth position in the beta-globin chain, promoting the formation of hemoglobin S (HbS) and the characteristic sickling of red blood cells, which underlies the clinical complications of the disease (Kato et al., 2018; Driss et al., 2009; Bunn, 1997; Pauling and Itano, 1949). The mutation results in abnormal, sickle-shaped red blood cells that obstruct blood flow, causing vaso-occlusion, organ damage, and systemic inflammation (Abdulmalik et al., 2020) as well as reduced life expectancy (Akinsheye and Klings, 2010). Sickle Cell Anemia (SCA), the most severe form, affects individuals homozygous for the HbS allele (HbSS) and is associated with acute complications such as anemia, infections and sepsis, as well as chronic issues including renal dysfunction, neurological decline, and impaired quality of life (Ware et al., 2017; Elmariah et al., 2014; Fitzhugh et al., 2010). Acute chest syndrome (ACS) and stroke present potentially life-threatening conditions that necessitate immediate and well-coordinated efforts for early detection as well as urgent and specialized care. Moreover, SCD significantly impacts cognitive and psychosocial functioning (Prussien et al., 2020). Individuals with the HbAS genotype (carriers of one mutated allele) are asymptomatic and are generally protected from severe manifestations, while those with HbSS experience a full spectrum of symptoms and complications (Ware et al., 2017; Eltzschig and Eckle, 2011; Lee et al., 2013).

One of the harmful by-products of SCD is cell-free heme, released during hemolysis, which triggers inflammation, oxidative stress, and vascular complications, including brain damage (Kato et al., 2017). Understanding the role of heme is essential for developing effective therapies. In 2021, an estimated 7.74 million individuals globally were affected by SCD (Thomson et al., 2023), with approximately 75% residing in sub-Saharan Africa (Wastnedge et al., 2018; Makani et al., 2011). In the United States, around 100,000 individuals, predominantly of African descent, are affected (CDC, 2024). Worldwide, SCA affects over 300, 000 newborns annually (WHO, 2024; Piel et al., 2013). A genetic survey estimates indicate SCD may account for approximately 50%–90% of child mortality in children under the age of five (Grosse et al., 2011). With reduced infectious disease mortality, SCD has become a significant contributor to childhood deaths from non-communicable diseases, making it a global health priority for achieving sustainable development goals (Ware et al., 2017; McGann, 2016). In Ghana, where SCD is highly prevalent, approximately 2% of newborns are diagnosed annually, with many carrying HbAS, HbSS, or HbSC genotypes (Oppong et al., 2020; Ohene-Frempong et al., 2008). The HbC variant, common in West Africa (Weatherall, 2008) is associated with milder forms of SCD complications and provides some protection against *Plasmodium falciparum* infections (Weatherall, 2008; Rihet et al., 2004; Kreuels et al., 2010; Driss et al., 2011; Iqbal et al., 2016). A review in Ghana’s largest teaching hospital revealed that SCD cases constituted a substantial proportion of the patient population, with many presenting for management of complications (Asare et al., 2018). Children with SCD in malaria-endemic regions are at high risk of complications, including severe anemia, chronic intravascular hemolysis, and painful vaso-occlusive crises (VOC), which can lead to silent cerebral infarcts (Platt, 2005; Hulbert et al., 2011) and Cerebral Malaria (CM) (Santaterra et al., 2020; Schiess et al., 2020).

Recent studies have underscored the high risk of stroke in children with SCA, especially without early intervention and treatment (Hulbert et al., 2011; Verduzco and Nathan, 2009; Chambliss et al., 2021). Limited healthcare access and financial constraints contribute to the high mortality rate among children with SCA in sub-Saharan Africa (Tshilolo et al., 2008). Early diagnosis of SCD is typically achieved through symptomatic presentation or neonatal screening. Thus, there is an urgent need to identify predictive markers for severe and potential risk of complications in these patients.

Recent research has highlighted the need for predictive biomarkers to improve early diagnosis, detection of complications and intervention in SCD (Rees and Gibson, 2012).

Brain-derived Neurotrophic Factor (BDNF), a nerve growth factor, plays a crucial role in neuronal survival, adaptation, and response to ischemic brain injury, with implications for endothelial cells (ECs) survival and neo-angiogenesis in ischemic tissues (Hyacinth et al., 2012; Chen et al., 2013; Bathina and Das, 2015; Kim and Winstein, 2017; Ramaswamy et al., 2009). Proinflammatory cytokines and chemokines, and angiogenic markers like Placental Growth Factor (PIGF) have been linked to SCD severity and complications (Zhang and An, 2007; Hasanvand, 2022; Korobova et al., 2023; Perelman et al., 2003; Gu et al., 2018). These biomarkers are crucial in understanding pathological mechanisms like hemolysis, inflammation, and endothelial dysfunction in SCD (Rees and Gibson, 2012). A detailed role of biomarkers associated with SCD is presented in Table 2. Currently, there is no reliable diagnostic test to measure plasma-free heme levels limiting clinicians' ability to assess heme-induced inflammation and its harmful effects (Immenschuh et al., 2017). The scavenger proteins help reduce the accumulation of cell-free Hb and free heme during excessive hemolysis by sequestering and facilitating their clearance (Balla et al., 2005; Schaer et al., 2013). Identifying robust biomarkers could improve disease outcome prediction, support personalized treatment strategies, and prevent irreversible complications. Early identification of these biomarkers could facilitate the development of personalized treatment strategies and improve patient outcomes (Conran and Belcher, 2018). Therefore, we investigated specific circulatory markers associated with inflammation and brain injury that might serve as indicators of life-threatening complications in children with heme-induced inflammation, such as in SCD. By analyzing these biomarkers across different genotypes, we seek to identify prognostic indicators that can inform clinical management and improve patient outcomes (Brousse et al., 2014). Additionally, this strategy could enhance differentiation between mild and severe SCD, deepen our understanding of its mechanisms, and improve clinical management. To our knowledge, this study is the first to explore circulating factors as both indicators and predictors of SCD progression and complications.

## Methods

### Ethical considerations

The present study received approval from the ethics boards of the Morehouse School of Medicine (approval number 1404521-9) and the University of Ghana College of Health Sciences (CHS-Et/M.6-P4.8/2021). Parents/guardians of participating children read and signed parental informed consent.

### Study subjects

This study enrolled volunteer children diagnosed with SCD, including HbSS and HbSC genotypes, Sickle cell trait (SCT) with HbAS and HbAC genotypes, and HbCC, along with a control group with normal HbAA. Eligible participants were children aged 3–8 years, confirmed through Hb Electrophoresis testing. Participants were recruited from the Child Health SCD Clinic at the Korle-Bu Teaching Hospital (KBTH), Accra, Ghana, between 2021 and 2022. Control participants were drawn from neighboring communities.

### Inclusion criteria

Eligible participants were children diagnosed with SCD (HbSS, HbSC), SCT (HbAS, HbAC), HbCC, or HbAA as a control group. All children aged 3–8 years old had their SCD status confirmed through hemoglobin electrophoresis. To ensure that inflammatory markers studied were solely attributed to SCD, only participants who were HIV-negative and not infected with *P. falciparum* were included in the study.

### Exclusion criteria

Children diagnosed with thalassemia syndromes, other hemoglobinopathies, leukemia, or other cancers were excluded from the study. Participants with no history of sickle cell crises or blood transfusions within the past 3 months were also excluded, as well as those with acute bacterial, viral, or parasitic infections, including P. falciparum. Additionally, individuals undergoing hydroxyurea therapy, children under the age of 2, and those who tested positive for HIV were excluded.

### Data collection, site and clinical assessment

A voluntary, in-person questionnaire was administered to gather health information, with a focus on SCD. The questionnaire, available in English and translated into local dialects (Ga, Twi, Ewe, and Hausa) as needed, collected data on recent infections requiring treatment, current pain status, age at diagnosis, frequency of pain episodes, ongoing treatments, and other SCD severity related information. Clinical data from children within the specified age group presenting with SCD, SCT, and other Hb genotypes at the Child Health Department (CHD) of KBTH were extracted using a standardized data abstraction method. This instrument documented clinic attendance/visits, phenotypes, *Plasmodium* parasitemia, hematological parameters, and complications. KBTH serves as a major pediatric referral center in Greater Accra and includes facilities such as La General Hospital, Princess Marie Louise Children Hospital, Kaneshie Polyclinic, Ussher Polyclinic, and Mamprobi Polyclinic. Additionally, the CHD houses the Sickle Cell Unit, a specialized unit catering to approximately 200 children biweekly, and an emergency unit for children experiencing crisis episodes.

### Sample size and selection

A total of 377 samples were collected between July 2021 and July 2022 as part of NIH/NINDS R01NS091616 (Stiles, PI) and NIH/FIC UJMT Fogarty Global Health Fellows Program #D43TW009340 (Chi PI; Lekpor, Fellow), and 1K01TW010282 (Driss, PI)-funded projects focusing on severe malaria at Morehouse School of Medicine (MSM), Atlanta, GA, USA, in collaboration with the Department of Pathology, University of Ghana Medical School. From this pool, we randomly selected 80 age- and sex-matched participants representing all hemoglobin (Hb) genotype groups. This subset consisted of 16 individuals, each with HbAA, HbSS, and HbSC, 14 individuals, each with HbAS and HbAC, and 4 with HbCC, all drawn from the Greater Accra region. The participants were selected using convenience sampling, which may introduce selection bias and may limit the representativeness of the larger population.

### Blood sample collection and processing

Blood samples were collected into sodium citrate tubes (Cat # 454322, Bio-ONE, United States), and plasma was isolated using SepMate™ tubes (STEM Cell Technologies, Cat# 854115) and processed within 4 h of collection. The isolated plasma samples were stored at −80°C and later thawed gradually at room temperature before analysis, as recommended by other groups (Grievink et al., 2016).

### Hemoglobin status determination

Hemoglobin status was determined using cellulose acetate membrane electrophoresis, following method described by Ngwengi et al. (Ngwengi et al., 2020). This analysis was conducted in the Hematology Department of Korle-Bu Teaching Hospital. Complete blood count (CBC) was performed on whole blood samples using an ABX Micros ES 60, an 18-parameter hematology analyzer, also within the Department of Hematology of the Korle Bu Teaching Hospital.

### Malaria and HIV testing

Malaria infection status was assessed using Rapid Diagnostic Test (RDTs) Kits (First Response® Malaria Ag. pLDH/HRP2 Combo Card Test kit (WHO reference number: PQDx0285-010-00, PI16FRC25), which detects Plasmodium falciparum-specific HRP2 and Pan lactate dehydrogenase (LDH) to identify multiple malaria species. This test was done to exclude malaria-positive participants. HIV status was determined using First Response® HIV-1–2 rapid diagnostic test (RDT) kits (Cat# PI05FRC30) to exclude HIV-positive participants.

### Multiplexed immunoassay measurement

The plasma levels of biomarkers were quantified using the MSD Multi-Spot Assay System MESO Scale QuickPlex® (SQ 120, MSD, Maryland, United States). This system was chosen for its sensitivity and specificity in measuring multiple cytokines simultaneously. The cytokine analysis was performed on customized U-plex plates and V-plex with undiluted plasma samples, adhering strictly to the manufacturer’s protocol. A comprehensive range of cytokines was targeted using the U-PLEX Development Pack (MSD® k15231N) and V-PLEX Plus Neuroinflammation Panel 1 (Meso-Scale Diagnostics Cat#: K151ACM-1). The U-PLEX enabled the complexing of selected biomarkers of interest, including Angiopoietin 1 (Ang 1), Angiopoietin 2 (Ang 2), Brain-Derived Neurotrophic Factor (BDNF), C-X-C motif chemokine ligand 10, CXCL10. It is also known as Interferon Gamma-Induced Protein 10 (IP-10), Interleukin-1 alpha (IL-1α), Interleukin-6 (IL-6), Heme Oxygenase-1, Haptoglobin, Hemopexin (HO-1, Hp, Hpx). The V-PLEX panel allowed for the simultaneous measurement of 37 cytokines, including C-Reactive Protein (CRP), Eotaxin, Eotaxin-3, Fibroblast Growth Factor (basic FGF), Intercellular Adhesion Molecule 1 (ICAM-1), Interferon Gamma (IFN-γ), a variety of interleukins (IL-1β, IL-2, IL-4, IL-5, IL-6, IL-7, IL-8, IL-10, IL-12/IL-23p40, IL-13, IL-15, IL-16, and IL-17A), CXCL10, Monocyte Chemoattractant Protein-1 (MCP-1), MCP-4, Macrophage-Derived Chemokine (MDC), Macrophage Inflammatory Protein-1 alpha (MIP-1α), MIP-1β, Placental Growth Factor (PlGF), Serum Amyloid A (SAA), Thymus and Activation-Regulated Chemokine (TARC), Tie-2, Tumor Necrosis Factor Alpha (TNF-α), TNF-β, Vascular Cell Adhesion Molecule 1 (VCAM-1), Vascular Endothelial Growth Factor A (VEGF-A), VEGF-C, VEGF-D, and VEGF Receptor 1 (VEGFR-1/Flt-1). Plasma heme levels were quantified in triplicates using 25 μL aliquots of each sample. A colorimetric assay (QuantiChrom™ Heme Assay Kit Cat# DIHM-250, BioAssay Systems, USA) was employed, and the assays were conducted at the MSM Core Lab. This approach was selected to represent a diverse range of high and low heme levels.

### Statistical analysis

The D'Agostino & Pearson normality test determined the data’s distribution. For normally distributed data, differences between two groups were evaluated using an unpaired two-tailed Student’s t-test. For data not following a normal distribution, the nonparametric Mann-Whitney U test was used for two-group comparisons (as detailed in Table S3). Statistical analyses were conducted using GraphPad PRISM version 9.5.1 for Windows 10 (GraphPad Software, La Jolla, California). Plasma biomarker concentrations were presented as mean ± standard deviation (SD). One-way ANOVA tests, supplemented by Tukey’s multiple comparison tests, were used to compare biomarker levels across different Hb subgroups and complete blood count parameters. Student's t-tests were employed to analyze differences in mean values for two-group comparisons. This approach allowed for the assessment of specific differences between groups. The study was powered based on preliminary multiplex immunoassay data from a subset of samples. Assuming a minimum detectable difference of 2 standard deviations between group means, a sample size of 10 per group was calculated to provide a minimum of 90% power at a 95% confidence level. Correlation analyses were performed using Pearson or Spearman coefficients, as appropriate. A *p*-value threshold of <0.05 was set for statistical significance, and exact *p*-values were reported. Cytokines, chemokines, growth factors and heme scavenger analyte concentrations outside the linear detection range of the immunoassays were excluded from the analysis.

### Predictive analysis using cytokine ratio calculations

The ratios of circulating biomarker levels across different Hb variants were calculated to identify distinct biomarker profile changes associated with Hb sickle status. Heme levels were measured and divided by the cytokine of interest for the heme-to-cytokine ratio to assess whether cell-free heme levels impact inflammation. Similarly, the circulating biomarker-to-scavenger ratio was used to explore how cytoprotective proteins might modulate inflammation. Additionally, ratios of two distinct inflammatory markers were analyzed to evaluate how specific interactions vary across different genotypes. By integrating both heme and other biomarkers, this approach facilitates the development of predictive algorithms to estimate the risk of severe complications due to hemolysis based on the cumulative effects of diverse inflammatory mediators associated with different genotypes.

### Receiver operating characteristics (ROC)

ROC curves were generated to assess the discriminative power of biomarker tests between different study groups. The Area Under the ROC Curve (AUC) was used to evaluate the performance of the diagnostic tests, focusing on their specificity and sensitivity in distinguishing between the groups. An AUC value closer to one indicates a higher discriminatory ability, implying that the test results more effectively separate the distributions of the analyzed groups. The ROC analysis determined cutoff values that optimized sensitivity and specificity. The curves and the associated AUC metrics quantified the overall accuracy and validity of the experimental tests in categorizing individuals based on their biomarker profiles, which indicate their specific disease or Hb sickle state (Harp et al., 2020). A *p*-value of <0.05 was set as the threshold for statistical significance in all tests.

### Principal component analysis (PCA)

PCA was conducted using Past 4.1.0 software (Hammer and Harper, 2001). This analysis covered biomarkers targets, scavengers, and their respective concentrations, including Ang 1, Ang 2, BDNF, TNF-α, CXCL10, IL-1α, IL-6, CXCL10, CCL11, IFN-γ, IL-10, TNF-α, MDC, MIP-1β, MCP-1, IL-1α, IL-6, IL-16, IL-12p40, VEGFA, VEGF-C, VEGF-D, PlGF, bFGF, Flt-1, Tie 2, HO-1, Hpx Hp, and complete blood count parameters [White Blood Cells (WBC), Red Blood Cells (RBC), Hemoglobin (Hb), and Platelets (PLT)]. For the PCA, although no data was missing, one low value due to measurement units was present and was excluded to ensure a consistent and comprehensive analysis. A correlation matrix analysis evaluated the relationships between the variables across different groups.

## Results

### Demographics and hematological findings

Significant variations in White Blood Cell (WBC) counts were observed among the groups, with the highest levels found in the HbSS group, followed by HbSC, HbAS, HbAC, and HbAA. Specifically, WBC counts were significantly higher in HbSS compared to HbAS (*p* = 0.0001), HbAA (*p* = 0.0107), HbAC (*p* = 0.0178), and HbSC (*p* = 0.0145). For Red Blood Cell (RBC) levels, the HbSS group had significantly lower levels compared to all other groups, with HbSS being lower than HbAA, HbAS, HbAC, HbSC, and HbCC (*p* < 0.0001 for all comparisons). Additionally, significant differences in RBC counts were observed between HbAA and HbSC (*p* = 0.0375), and between HbSC and HbCC (*p* = 0.0156) and Platelet (PLT) counts differed significantly in HbSS compared to other Hb genotypes (*p* < 0.0001) (Table 1; Supplementary Table S1).

**TABLE 1:** Mean and standard deviation of clinical characteristics of all sickle Hb genotypes groups of individuals based defined. There was significant difference in age and gender distribution among the groups.

Mean ± SD	HbAA n = 16	HbAS n = 14	HbSS n = 16	HbAC n = 14	HbSC n = 16	HbCC n = 4	Normal ranges
Male/female	8/8	7/7	8/8	8/6	8/8	2/2	
Age (years)	4.44 ± 1.89	5.0 ± 2.18	6.06 ± 1.53	4.0 ± 1.41	6.0 ± 2.19	6.50 ± 2.38	
WBC (x 10^3^/mm^3^)	8.82 ± 3.79	7.14 ± 1.88	12.71 ± 4.04	8.88 ± 1.74	8.93 ± 3.61	8.46 ± 0.80	4.0–12.0/4.5–14.50
RBC (x 10^3^/µL)	4.50 ± 0.49	4.38 ± 0.50	2.42 ± 0.59	4.22 ± 0.49	3.77 ± 0.65	5.15 ± 0.34	3.5 to 5.2/4.0 to 5.5
Hemoglobin (g/dL)	11.67 ± 0.6	11.0 ± 1.46	7.61 ± 1.28	9.8 ± 1.49	9.90 ± 0.90	10.9 ± 0.57	12.0 to 16.0/11.0 to 16.0
Hematocrit (%)	34.25 ± 1.87	31.85 ± 3.98	22.31 ± 3.72	47.11 ± 72.71	27.69 ± 2.87	31.62 ± 2.65	34.0–49.0
MCV (fL)	76.60 ± 7.08	73.05 ± 8.28	94.56 ± 12.54	65.76 ± 5.08	71.35 ± 22.02	61.72 ± 8.22	80.0 to 100.0/80.0 to 95.0
MCH (pg)	26.15 ± 2.87	25.22 ± 3.26	32.19 ± 4.18	23.14 ± 2.13	26.78 ± 4.57	20.92 ± 2.19	27.0–34.0
MCHC (g/dL)	34.11 ± 0.97	34.49 ± 0.89	34.03 ± 0.87	35.12 ± 0.94	35.76 ± 0.76	34.00 ± 1.00	31.0–37.0
PLT (x10^3^/µl)	298.5 ± 103.42	339.71 ± 72.3	441.6 ± 118.1	322.9 ± 82.61	341.6 ± 117.9	387.7 ± 155.12	100–300/150–450

As expected, there was a significant reduction in Hb levels in the sickle cell group compared with other Hb genotypes. Hb levels significantly differed between HbSS *versus* the following: HbAA (*p* < 0.0001), HbAS (*p* < 0.0001), and HbCC (*p* = 0.0001). Furthermore, MCV (*p* < 0.001), levels were higher in the HbSS group compared to the other groups, whilst MCH (*p* = 0.0045), and MCHC (*p* < 0.0001) were generally normal across the other Hb groups (Table 1). MCV may be slightly elevated due to the presence of larger reticulocytes but remains within the normal range. This elevation is not related to hydroxyurea use as patients on hydroxyurea were excluded from this study. Supplementary Table S1 provides all other statistically significant hematologic comparisons between Hb subgroups.

### Variations of circulating plasma inflammatory markers across different Hb genotypes

**FIGURE 1:**
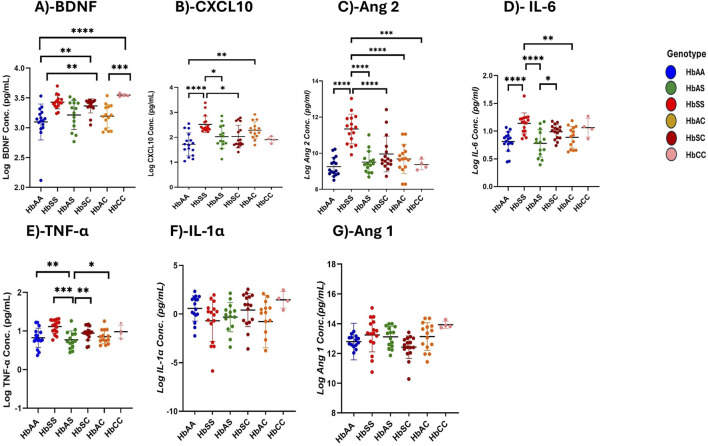
Differential Expression of Inflammatory, Vascular, and Neurotrophic Markers Across Sickle Hemoglobin Genotypes. Multiple comparisons for each inflammatory marker using one-way ANOVA with Turkey’s comparison tests. Scatter plots indicate minimum and maximum mean plasma values observed. The following significant variations were observed across different sickle Hb genotypes: **(A)** BDNF: HbAA vs. HbSS (*p* < 0.0001), HbAA vs. HbCC (*p* < 0.0001), HbSS vs. HbAC (*p* = 0.0017), HbAC vs. HbCC (*p* = 0.0005) and HbAA vs. HbSC (*p* = 0.0069). **(B)** CXCL10: HbAA vs HbSS (P< 0.0001), HbAA vs HbAC (P = 0.0040) and HbSS vs HbSC (P = 0.0116) and HbSS vs HbAS (P = 0.0142). **(C)** Ang 2: HbSS vs. HbAA (*p* < 0.0001), HbSS vs. HbAS (*p* < 0.0001), HbSS vs. HbAC (*p* < 0.0001), HbSS vs. HbSC (*p* < 0.0001) and HbSS vs. HbCC (*p* = 0.0002). **(D)** IL-6: HbAA vs. HbSS (*p* < 0.0001) HbAS vs. HbSS (*p* < 0.0001), HbSS vs. HbAC (*p* = 0.0062) and HbAS vs. HbSC (*p* = 0.0349). **(E)** TNF-α: HbAA vs. HbSS (*p* = 0.0011), HbAS vs. HbSS (*p* = 0.0002) and HbSS vs. HbAC (P0.0017), HbSS vs. HbSC (*p* = 0.003). **(F)** Assessment of IL-1α in different sickle Hb genotypes: no significant differences observed. **(G)** Assessment of Ang 1 in different sickle Hb genotypes: no significant differences observed. Statistical significance are indicated as *p < 0.03–0.01; **p < 0.009–0.001; ***p < 0.0002–0.00019; ****p < 0.0001 ≤ 0.00001.

Mean plasma concentrations of various inflammatory markers were analyzed across different subgroups of sickle Hb genotypes to examine potential differences in their profiles associated with specific genotypes (Figure 1; Supplementary Table S2).

BDNF levels were significantly higher in individuals with SCD genotypes following the order HbSS > HbCC > HbSC > HbAS > HbAC > HbAA. Significant plasma mean differences were observed between HbAA and HbSS (1,460 ± 716.5 pg/mL vs. 2,816 ± 190.3 pg/mL, *p* < 0.0001), HbSS and HbAC (2,816 ± 190.3 pg/mL vs. 1707 ± 762.0 pg/mL, *p* = 0.0017), HbAA and HbCC (1,460 ± 716.5 pg/mL vs. 2,784 ± 795.6 pg/mL, *p* < 0.0001), HbAC and HbCC (1707 ± 762.0 pg/mL vs. 1,460 ± 716.5 pg/mL, *p* = 0.0005), and HbAA and HbSC (1,460 ± 716.5 pg/mL vs. 2,390 ± 523.4 pg/mL, *p* = 0.0069) (Figure 1A). CXCL10 concentrations were highest in the order HbSS > HbAS > HbSC > HbAC > HbAA > HbCC with significant mean plasma levels observed between HbAA and HbSS (1719 ± 458.5 pg/mL vs. 2,518 ± 334.1 pg/mL, *p* = 0.002), HbAA and HbSC (1719 ± 458.5 pg/mL vs. 2030 ± 454.8 pg/mL, *p* = 0.0089), HbSS and HbAC (2,518 ± 334.1 pg/mL vs. 2,282 ± 337.3 pg/mL, P0.0361) (Figure 1B). Plasma mean Ang 2 levels were significantly elevated in HbSS than HbAA (1,134 ± 82.1 pg/mL vs. 925.2 ± 52.0 pg/mL, *p* = 0.002), HbSS and HbAS (1,134 ± 82.1 pg/mL vs. 950.6 ± 60.7 pg/mL, *p* < 0.0001), HbSS and HbAC (1,134 ± 82.1 pg/mL vs. 968.2 ± 81.3 pg/mL, *p* < 0.0001), HbSS and HbSC (1,134 ± 82.1 pg/mL vs. 995.2 ± 98.2 pg/mL, *p* = 0.0089), HbSS and HbCC (1,134 ± 82.1 pg/mL vs. 937.0 ± 28.6 pg/mL, *p* = 0.0002), showing that HbSS had the highest concentrations across the groups (Figure 1C). IL-6 concentrations varied significantly, with higher levels in HbSS > HbSC > HbAS > HbAC > HbCC > HbAA. Significant plasma mean differences were observed between HbAA and HbSS (811.6 ± 176.3 pg/mL vs. 1,138 ± 188.3 pg/mL, *p* < 0.0001), HbAS and HbSS (777.3 ± 245.7 pg/mL vs. 1,138 ± 188.3 pg/mL, *p* < 0.0001), HbSS and HbAC (1,138 ± 188.3 pg/mL vs. 887.3 ± 193.8 pg/mL, *p* = 0.0062), and HbAS and HbSC (777.3 ± 245.7 pg/mL vs. 987.6 ± 129.3 pg/mL, *p* = 0.0349), (Figure 1D). Similarly, mean plasma TNF-α levels were highest in the order HbSS > HbSC > HbCC > HbAC > HbAS > HbAA with significant plasma mean levels between HbAA and HbSS (775.0 ± 444.8 pg/mL vs1397 ± 499.0 pg/mL, *p* = .0011), HbAS and HbSS (679.0 ± 409.4 pg/mL vs. 1,397 ± 499.0 pg/mL, *p* = .0002), HbSS and HbAC (1,397 ± 499.0 pg/mL vs. 774.6 ± 374.0 pg/mL, *p* = 0.0017), and HbSS and HbCC (1,397 ± 499.0 pg/mL vs. 979.0 ± 348.8 pg/mL, *p* = 0.003) (Figure 1E). No significant differences were found in IL-1α and Ang 1 across the different Hb genotypes, indicating comparable levels among all groups (Figures 1F, G). Additionally, there were no significant differences between males and females for all biomarkers.

### Children with SCD exhibit elevated heme and HO-1 levels but reduced Hp and Hpx compared to other sickle Hb genotypes

**FIGURE 2:**
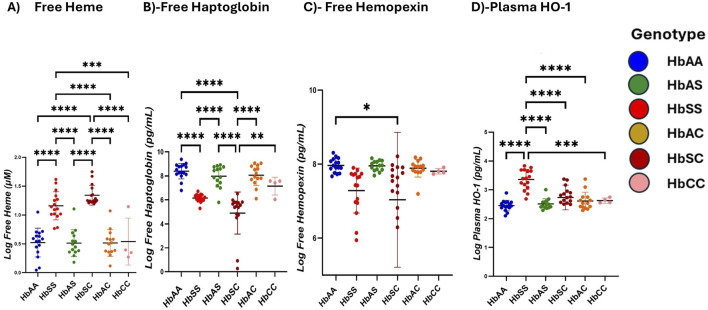
Scavenger proteins differ based on different sickle hemoglobin genotypes. Multiple comparison tests between Heme, heme scavengers, and sickle Hb genotypes using a one-way ANOVA test show significant differences in heme and scavenger protein expression across all sickle Hb genotypes. Scatter plots indicate minimum and maximum mean plasma values. The following assessments were made in: **(A)** Free heme levels (µM) between HbSS vs. HbAA (*p* < 0.0001), HbSS vs. HbAS (*p* < 0.0001), HbSS vs. HbAC (*p* < 0.0001) and HbSS vs. HbCC (*p* = 0.0002). Also, in HbAA vs. HbSC (*p* < 0.0001), (HbAS vs. HbSC (*p* < 0.0001), HbAS vs. HbAC (*p* < 0.0001), HbAC vs. HbSC (*p* < 0.0001) and HbSC vs. HbCC (*p* < 0.0001). **(B)** Haptoglobin levels (Pg/mL) in all genotypes: HbSS vs. HbAA (*p* < 0.0001), HbSC vs. HbAA (*p* < 0.0001), HbAS vs. HBSC (*p* < 0.0001), HbAC vs. HbAC (*p* < 0.0001), HbSS vs. HbAC (*p* = 0.0007) and HbAS vs. HbSS (*p* = 0.0013). **(C)** Hemopexin levels (Pg/mL) in HbAA vs. HbSC (*p* = 0.0416). **(D)** Plasma HO-1 levels (Pg/mL): HbSS vs. HbAA (*p* < 0.0001), HbSS vs. HbAS (*p* < 0.0001), HbSS vs. HbAC (*p* < 0.0001), HbSS vs. HbSC (*p* < 0.0001) and HbSS vs. HbCC (*p* = 0.0005). Statistical significances are indicated as *p < 0.03–0.01; **p < 0.009–0.001; ***p < 0.0002–0.00019; ****p < 0.0001 ≤ 0.00001.

Heme and heme scavenger (Hp, Hpx, HO-1) levels were compared across different Hb subgroups. Plasma-free heme levels were highest in the order HbSC > HbSS > HbAS > HbAS > HbCC > HbAA, with significantly higher levels between HbSS and HbAA (1,160 ± 243.9 vs. 523.4 ± 249.0 pg/mL, *p* < 0.0001), HbSS and HbAS (1,160 ± 243.9 vs. 513.0 ± 230.7 pg/mL, *p* < 0.0001), HbSS and HbAC (1,160 ± 243.9 vs. 513.9 ± 231.9 pg/mL, *p* < 0.0001), HbSS and HbCC (1,160 ± 243.9 vs. 406.7 ± 203.3 pg/mL, *p* = 0.0002) and also higher in HbSC (1,169 ± 171.5 pg/mL) compared to HbAA, HbAS, HbAC, and HbCC (all *p* < 0.0001) (Figure 2A; Supplementary Table S3). Hp levels showed significant differences across the subgroups, with the highest levels in HbAA > HbAS > HbAS > HbAC > HbCC > HbSC > HbSS. Significant plasma mean differences were observed between HbAA and HbSS (839.4 ± 64.8 vs. 614.6 ± 32.8 pg/mL, *p* < 0.0001), HbAA and HbSC (839.4 ± 64.8 vs. 489.9 ± 17.5 pg/mL, *p* < 0.0001), HbAS and HbSC (797.2 ± 90.6 vs489.9 ± 17.5 pg/mL, *p* < 0.0001), HbAC and HbCC (805.5 ± 86.6 vs. 714.8 ± 74.6 pg/mL, *p* < 0.0001), HbSS and HbAC (614.6 ± 32.8 vs. 805.5 ± 86.6 pg/mL, *p* = 0.0007) and HbAS and HbSS (797.2 ± 90.6 vs. 614.6 ± 32.8 pg/mL, *p* = 0.0013) (Figure 2B). Plasma mean Hpx levels were significantly different only between HbAA and HbSS (796.6 ± 18.6 vs. 728.7 ± 60.6 pg/mL, *p* = 0.0416) (Figure 2C). HO-1 levels were highest in the order HbSS > HbSC > HbAC > HbAS > HbAA > HbCC, with HbSS (336.1 ± 34.6 pg/mL) showing significantly higher levels compared to all other subgroups (HbAA, HbAS, HbAC, HbSC) (all *p* < 0.0001) and (HbCC, *p* = 0.0005) (Figure 2D).

### Heme increased inflammatory marker expression across sickle Hb genotypes

**FIGURE 3:**
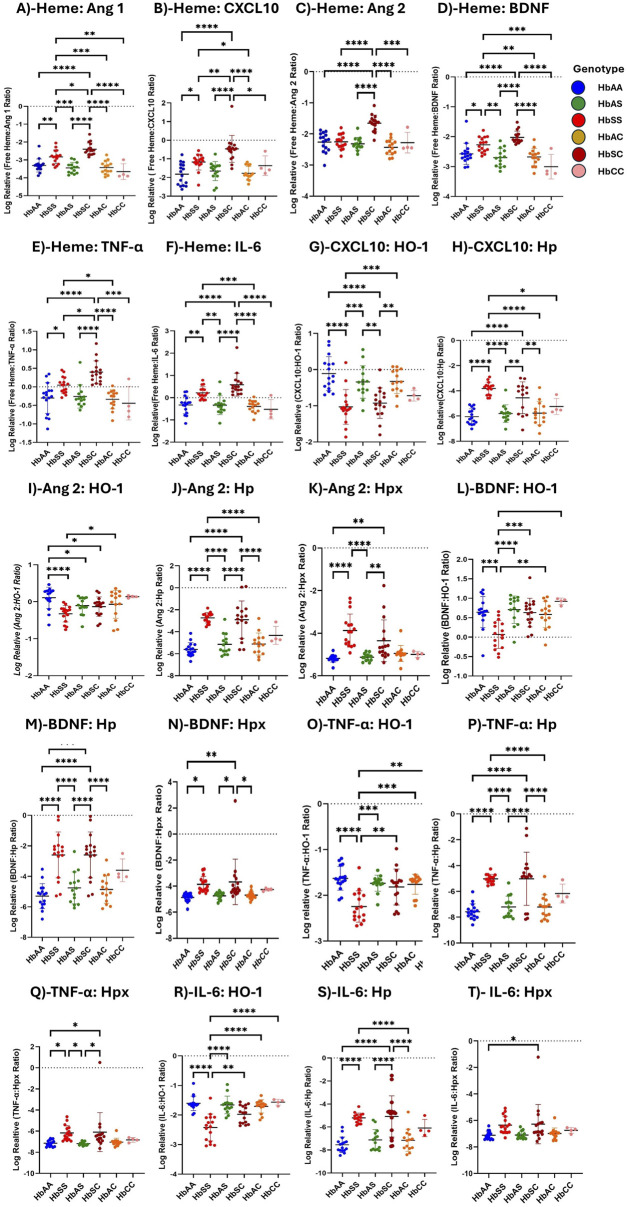
Assessment of Heme, Heme scavengers, and Inflammatory marker ratios across all sickle Hb genotypes using multiple comparison tests. Log-relative heme-to-biomarker ratios were assessed to evaluate heme levels related to inflammatory markers and scavenger protein-to-biomarker ratios were used to evaluate inflammatory markers relative to scavenger proteins. Mean plasma ratios are represented by scatter plots showing maximum and minimum values. Specific mean plasma differences were observed in the following groups: **(A)** Heme:Ang 1: HbAA vs. HbSS (−3.310 ± 0.363 vs. −2.828 ± 0.380, *p* = 0.0037); HbAA vs. HbSC (−3.310 ± 0.363 vs. −2.397 ± 0.342, *p* < 0.0001); HbSS vs. HbAS (−2.828 ± 0.380 vs. −3.447 ± 0.305, *p* = 0.0001); HbSS vs. HbSC (−2.397 ± 0.342, *p* = 0.0131); HbSS vs. HbAC (−342.8 ± 36.4, *p* = 0.0003); HbSS vs. HbCC (−3.653 ± 0.444, *p* = 0.0013); HbAS vs. HbSC (−3.447 ± 0.305 vs. −2.397 ± 0.342, *p* < 0.0001); HbSC vs. HbAC (µ-2.397 ± 0.342 vs-342.8 ± 36.4, *p* < 0.0001); HbSC vs. HbCC (−3.447 ± 0.305 vs-3.653 ± 0.444, *p* < 0.0001). **(B)** Heme:CXCL10: HbAA vs. HbSS (−1.8210 ± 5.879 vs. −1.1680 ± 4.243, *p* = 0.0127); HbAA vs. HbSC (−1.8210 ± 5.879 vs. −0.4640 ± 7.215, *p* < 0.0001); HbSS vs. HbSC (−1.1680 ± 4.243 vs. −0.4640 ± 7.215, *p* = 0.0056), HbSS vs. HbAC (−1.1680 ± 4.243 vs. −1.7690 ± 3.629, *p* = 0.0372); HbAS vs. HbSC (−2.3160 ± 2.032 vs. −0.4640 ± 7.215, *p* < 0.0001); HbSC vs. HbAC (−0.4640 ± 7.215 vs. −1.7690 ± 3.629, *p* < 0.0001); HbSC vs. HbCC (−0.4640 ± 7.215 vs. −1.3640 ± 5.272, *p* = 0.0434). **(C)** Heme:Ang 2: HbAA vs. HbSC (−2.2620 ± 3.088 vs. 2.023 ± 210.6, *p* < 0.0001); HbSS vs. HbSC (−2.2530 ± 2.356 vs. −1.6530 ± 2.574, *p* < 0.0001); HbAS vs. HbSC (−2.702 ± 3.044 vs. −1.6530 ± 2.574, *p* < 0.0001); HbSC vs. HbAC (−1.6530 ± 2.574 vs. −2.6780 ± 2.899, *p* < 0.0001); HbSC vs. HbCC (−1.6530 ± 2.574 vs. −2.2810 ± 3.306, *p* = 0.0006). **(D)** Heme:BDNF: HbAA vs. HbSS (−2.5730 ± 3.555 vs. −2.2700 ± 2.503, *p* = 0.0489); HbAA vs. HbSC (−2.5730 ± 3.555 vs. −2.023 ± 210.6, *p* < 0.0001); HbSS vs. HbAS (−2.2700 ± 2.503 vs. −2.702 ± 3.044, *p* = 0.0017); HbSS vs. HbAC (−2.2700 ± 2.503 vs. −2.6780 ± 2.899, *p* = 0.0036); HbAS vs. HbSC (−2.702 ± 3.044 vs. −2.023 ± 210.6, *p* < 0.0001); HbSC vs. HbAC (−2.023 ± 210.6 vs. −2.6780 ± 2.899 *p* < 0.0001); HbSC vs. HbCC (−2.023 ± 210.6 vs. −3.0060 ± 4.114, *p* < 0.0001). **(E)** Heme:TNF-α: HbAA vs. HbSS (−0.300 ± 0.414 vs. 0.2208 ± 0.273, *p* = 0.0334); HbAA vs. HbSC (−0.300 ± 0.414 vs. 0.3986 ± 0.304, *p* > 0.0001); HbSS vs. HbSC (0.2208 ± 0.273 vs. 0.3986 ± 0.304, *p* = .00276); HbSS vs. HbAC (0.2208 ± 0.273 vs. −0.3890 ± 0.271, *p* = 0.0184); HbAS vs. HbSC (−0.3365 ± 0.424 vs. 0.3986 ± 0.304, *p* < 0.0001); HbSC vs. HbAC (0.3986 ± 0.304 vs-0.3890 ± 0.271, *p* < 0.0001); HbSC vs. HbCC (0.3986 ± 0.304 vs. µ = −0.4419 ± 0.311, *p* = 0.0001). **(F)** Heme:IL-6: HbAA vs. HbSS (−0.3256 ± 0.394 vs. 0.2208 ± 0.273, *p* = 0.0023); HbAA vs. HbSC (−0.3256 ± 0.394 vs. 0.5846 ± 0.515, *p* < 0.0001); HbSS vs. HbAS (0.2208 ± 0.273 vs. −0.3365 ± 0.424, *p* = 0.0028); HbSS vs. HbAC (0.2208 ± 0.273 vs. 0.2208 ± 0.273, *p* = 0.0008); HbAS vs. HbSC (−0.3365 ± 0.424 vs. 0.5846 ± 0.515, *p* < 0.0001); HbSC vs. HbAC (0.5846 ± 0.515 vs. 0.2208 ± 0.273, *p* < 0.0001); HbSC vs. HbCC (0.5846 ± 0.515 vs. −0.5202 ± 0.435, *p* < 0.0001). **(G)** CXCL10:HO-1: HbAA vs. HbSS (0.1090 ± 0.467 vs. 1.032 ± 0.480, *p* < 0.0001); HbAA vs. HbSC (0.1090 ± 0.467 vs. −0.9271 ± 0.421, *p* < 0.0001); HbSS vs. HbAS (1.032 ± 0.480 vs. −0.3424 ± 0.449, *p* = 0.0005); HbSS vs. HbAC (1.032 ± 0.480 vs. −0.3271 ± 0.333, *p* = 0.0003); HbAS vs. HbSC (−0.3424 ± 0.449 vs. −0.9271 ± 0.421, *p* = 0.0048); HbSC vs. HbAC (−0.9271 ± 0.421 vs. −0.3271 ± 0.333, *p* = 0.0035). **(H)** CXCL10:Hp: HbAA vs. HbSS (−6.050 ± 0.622 vs. −3.818 ± 0.502, P= <0.0001); HbAA vs. HbSC (−6.050 ± 0.622 vs. −4.551 ± 1.262, *p* < 0.0001); HbSS vs. HbAS (−3.818 ± 0.502 vs. −5.806 ± 0.695, *p* = 0.0006); HbSS vs. HbAC (−3.818 ± 0.502 vs. −5.772 ± 0.927, *p* = 0.0008); HbAS vs. HbSC (−5.806 ± 0.695 vs. −4.551 ± 1.262, *p* = 0.0004); HbSC vs. HbAC (−4.551 ± 1.262 vs. −5.772 ± 0.927, *p* = 0.0006). **(I)** Ang 2: HO-1: HbAA vs. HbSS (0.332 ± 0.219 vs. 0.052 ± 0.371, *p* < 0.0001); HbAA vs. HbAS (0.332 ± 0.219 vs. 0.321 ± 0.157, *p* = 0.0153); HbAA vs. HbSC (0.332 ± 0.219 vs. 0.261 ± 0.547, *p* = 0.0101); and HbSS vs. HbAC (0.052 ± 0.371 vs. 0.334 ± 0.301, *p* = 0.0101). **(J)** Ang 2:Hp: HbAA vs. HbSS (−5.609 ± 0.617 vs. −2.733 ± 0.469, *p* < 0.0001); HbAA vs. HbSC (−5.609 ± 0.617 vs. −2.901 ± 1.700 *p* < 0.0001); HbSS vs. HbAS (−2.733 ± 0.469 vs. −5.143 ± 0.946, *p* < 0.0001); HbSS vs. HbAC (−2.733 ± 0.469 vs. −5.111 ± 1.004, *p* < 0.0001); HbAS vs. HbSC (−5.143 ± 0.946 vs. −2.901 ± 1.700, *p* < 0.0001); HbSC vs. HbAC (−2.901 ± 1.700 vs. −5.111 ± 1.004, *p* < 0.0001). **(K)** Ang 2:Hpx: HbAA vs. HbSS (−5.180 ± 0.168 vs. −3.874 ± 0.778, *p* = 0.0061); HbAA vs. HbSC (−5.180 ± 0.168 vs. −4.354 ± 0.977, *p* = 0.0244); HbSS vs. HbAS (−3.874 ± 0.778 vs. −5.12 ± 0.171, *p* = 0.0141); HbAS vs. HbSC (−5.12 ± 0.171 vs. −4.354 ± 0.977, *p* = 0.0493). **(L)** BDNF:HO-1: HbAA vs. HbSS (0.643 ± 0.399 vs. 0.069 ± 0.359, *p* = 0.0003); HbSS vs. HbAS (0.069 ± 0.359 vs. 0.706 ± 0.352, *p* < 0.0001); HbSS vs. HbSC (0.069 ± 0.359 vs. 0.631 ± 0.370, *p* = 0.0004); HbSS vs. HbAC (0.069 ± 0.359 vs. 0.582 ± 0.339, *p* = 0.0027); HbSS vs. HbCC (0.069 ± 0.359 vs. 0.920 ± 0.084, *p* = 0.0009). **(M)** BDNF:Hp: HbAA vs. HbSS (5.298 ± 0.818 vs. −2.594 ± 1.503, *p* < 0.0001); HbAA vs. HbSC (−5.298 ± 0.818 vs. −2.594 ± 1.503, *p* < 0.0001); HbSS vs. HbAS (−2.594 ± 1.503 vs. −4.757 ± 1.048, *p* = 0.0007); HbSS vs. HbAC (−2.594 ± 1.503 vs. = -4.862 ± 0.905, *p* = 0.0004); HbAS vs. HbSC (−4.757 ± 1.048 vs. −2.594 ± 1.503, *p* = 0.0007); HbSC vs. HbAC (−2.594 ± 1.503 vs. −4.862 ± 0.905, *p* = 0.0004). **(N)** BDNF:Hpx: HbAA vs. HbSS (−4.870 ± 0.323 vs. −3.857 ± 0.599, *p* = 0.0163); HbAA vs. HbSC (−4.870 ± 0.323 vs. −3.671 ± 1.742, *p* = 0.0024); HbAS vs. HbSC (−1.736 ± 0.177 vs. −3.671 ± 1.742, *p* = 0.0136); HbSC vs. HbAC (−3.671 ± 1.742 vs. −4.697 ± 0.298, *p* = 0.0200). **(O)** TNF-α:HO-1: HbAA vs. HbSS (−1.630 ± 0.260 vs. −2.246 ± 0.342, *p* < 0.0001); HbSS vs. HbAS (−2.246 ± 0.342 vs. −1.736 ± 0.177, *p* = 0.0004); HbSS vs. HbSC (−2.246 ± 0.342 vs. −1.819 ± 0.392*p* = 0.0012); HbSS vs. HbAC (−2.246 ± 0.342 vs. −1.759 ± 0.219, *p* = 0.0007); HbSS vs. HbCC (−2.246 ± 0.342 vs. −1.643 ± 0.094, *p* = 0.0107). **(P)** TNF-α:Hp: HbAA vs. HbSS (−7.570 ± 0.610 vs. −5.031 ± 0.345, *p* < 0.0001); HbAA vs. HbSC (−7.570 ± 0.610 vs. −5.024 ± 2.058, P < *p* < 0.0001); HbSS vs. HbAS (−5.031 ± 0.345 vs. −7.200 ± 0.830, *p* < 0.0001); HbSS vs. HbAC (−5.031 ± 0.345 vs. −7.204 ± 0.942, *p* < 0.0001); HbAS vs. HbSC (−7.200 ± 0.830 vs. −5.024 ± 2.058, *p* < 0.0001); HbSC vs. HbAC (−5.024 ± 2.058 vs. −7.204 ± 0.942, *p* < 0.0001); **(Q)** TNF-α;Hpx: HbAA vs. HbSS (−7.142 ± 0.298 vs. −6.172 ± 0.711, *p* < 0.042); HbAA vs. HbSC (−7.142 ± 0.298 vs. −6.192 ± 1.843, *p* < 0.021); HbSS vs. HbAS (−6.172 ± 0.711 vs-7.181 ± 0.160, *p* < 0.021); HbAS vs. HbSC (−7.181 ± 0.160 vs. −6.192 ± 1.843, *p* < 0.021). **(R)** IL-6:HO-1: HbAA vs. HbSS (=−1.604 ± 0.244 vs. −2.421 ± 0.455, *p* < 0.0001); HbSS vs. HbAS (−2.421 ± 0.455 vs. −1.659 ± 0.297, *p* < 0.0001); HbSS vs. HbSC (−2.421 ± 0.455 vs. −1.976 ± 0.295, *p* = 0.0017); HbSS vs. HbAC (−2.421 ± 0.455 vs. −1.707 ± 0.223, *p* < 0.0001); HbSS vs. HbCC (−2.421 ± 0.455 vs. −1.565 ± 0.097, *p* < 0.0001); **(S)** IL-6:Hp: HbAA vs. HbSS (−7.545 ± 0.674 vs. −5.207 ± 0.426, *p* < 0.0001); HbAA vs. HbSC (−7.545 ± 0.674 vs. −4.828 ± 1.792, *p* < 0.0001); HbSS vs. HbAC (−5.207 ± 0.426 vs. −7.152 ± 0.963, *p* = 0.0009); HbAS vs. HbSC (−7.122 ± 0.907 vs. −4.828 ± 1.792, *p* < 0.0001); HbSC vs. HbAC (−4.828 ± 1.792 vs. −7.152 ± 0.963, *p* < 0.0001); **(T)** IL-6:Hpx: HbAA vs. HbSC (−7.117 ± 0.303 vs. −6.278 ± 1, *p* = 0.035). Statistical significance are indicated as *: *p* < 0.03–0.01; **: *p* < 0.009–0.001; ***: *p* < 0.0002–0.00019; ****: *p* < 0.0001 ≤ 0.00001.

Ratios of heme, heme scavengers, and biomarker levels were compared across sickle Hb genotypes, including ratios of biomarkers to scavenger proteins. Heme and its scavengers significantly influenced the levels of Ang 1, CXCL10, Ang 2, TNF-α, IL-6, and BDNF across different Hb genotypes, with multiple significant differences observed. Comparisons of biomarker-to-scavenger ratios indicate that scavenger proteins play a critical role in modulating circulatory biomarker expression (Figure 3). Heme: Ang 1 ratio was increased in HbSC > HbSS > HbAA > HbAS > HbAC > HbCC, with significant differences between HbSS and all other groups, and also between HbSC and the other genotypes (Figure 3A). Also, Heme: CXCL10 levels followed the order HbSC > HbSS > HbAS > HbCC > HbAA > HbAC, with significant differences between HbAA and HbSS, HbAA and HbSC, HbSS and HbSC, HbSS and HbAC, HbAS and HbSC, HbSC and HbAC, and HbSC vs. HbCC (Figure 3B).

Heme: Ang 2 showed significant variations, with higher levels in HbSC > HbSS > HbAS > HbAC > HbAA > HbAC. Significant differences were observed between HbAA vs. HbSC, HbSS vs. HbSC, HbAS vs. HbSC, HbSC vs. HbAC, and HbSC vs. HbCC (Figure 3C). Furthermore, Heme: BDNF levels were highest in the order HbSC > HbSS > HbAS > HbAC > HbAA > HbCC, with significant differences between several groups, including HbAA vs. HbSS, HbAA vs. HbSC, HbSS vs. HbAS, HbSS vs. HbAC, HbAS vs. HbSC, HbSC vs. HbAC, and HbSC vs. HbCC (Figure 3D).

Heme: TNF-α and Heme: IL-6 ratios showed significant differences across multiple comparisons, with HbSC consistently having the highest levels, followed by HbSS, both significantly higher than those of other groups (Figures 3E, F).

Consequently, with biomarker-scavenger-ratio, CXCL10: HO-1 and CXL10: Hp ratios significantly differed in HbSS, HbSC, HbAA, HbAS, HbAC, and HbCC. In the CXCL10: HO-1 ratio, HbAA showed a higher ratio compared to all other genotypes in the order HbAA > HbAS > HbAC > HbSC > HbSS > HbCC, whereas the CXL10: Hp ratio was significantly higher in HbSS > HbSC > HbAS > HbAC > HbCC > HbAA (Figures 3G, H). Ang 2: HO-1 ratio was altered following the order HbAA > HbAC > HbAS > HbSC > HbCC > HbSS. However, Ang 2:Hp and Ang 2:Hpx ratios indicated elevated levels in HbSC and HbSS, respectively, compared to the other Hb genotypes, with significant differences across groups, including HbAA vs. HbSS, HbAA vs. HbSC, and HbSS vs. HbAC (Figures 3I–K). Further, BDNF: Hp and BDNF: Hpx ratios showed variations across genotypes, with the highest ratios in HbSS and HbSC and lower levels in HbAA, HbAS, HbAC, and HbCC, with significant differences between several comparisons. However, BDNF: HO-1 showed higher levels in the order HbAA > HbAS > HbAC > HbCC > HbSC > HbSS (Figures 3L–N). Additionally, TNF-α: HO-1, TNF-α: Hp, and TNF-α:Hpx ratios were significantly higher in HbSC and HbSS compared to HbAS, HbSC and HbAA groups in all comparisons, indicating a strong inflammatory response (Figures 3O–Q). Furthermore, IL-6: Hp and IL-6: Hpx levels showed a pattern of HbSC > HbSS > HbAS > HbAA, with significant alterations observed between several groups. IL-6: HO-1, however, showed a decreased ratio in HbSS and HbSC compared to HbAA, HbAS, HbAC, and HbCC (Figures 3R–T).

### Ratios of circulating inflammatory markers differ among children with different sickle Hb genotypes

**FIGURE 4:**
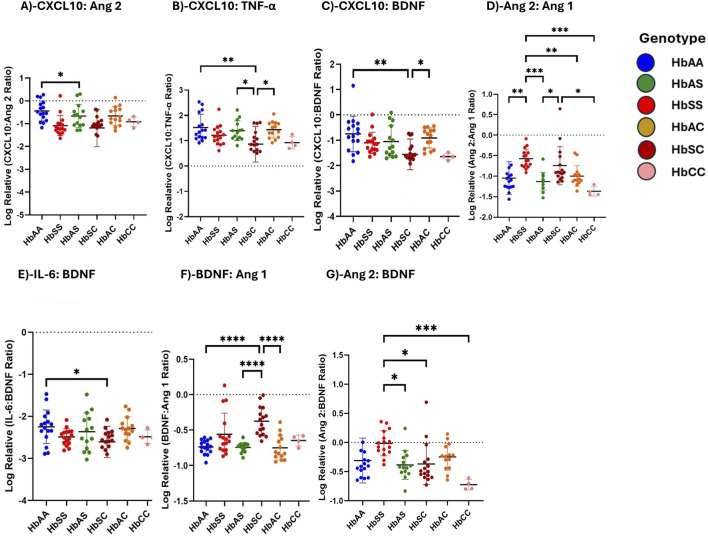
Ratios of Circulating Inflammatory Markers Differ Across Different Sickle Hb Genotypes. Assessment of inflammatory marker concentration ratios across different sickle Hb genotypes. Multiple comparisons test was used to evaluate individual biomarker ratios between each Hb genotype. The mean plasma levels of log-transformed relative individual biomarker ratios were analyzed to determine varied interactions between inflammatory markers across the sickle Hb genotypes. The following mean plasma ratios differ significantly across sickle Hb genotypes: **(A)** CXCL10:Ang 2: HbAA vs. HbSC (−0.441 ± 0.443 vs. −1.189 ± 0.817 *p* = 0.0461). **(B)** CXCL10:TNF-α: HbAA vs. HbSC (1.521 ± 0.524 vs. 0.863 ± 0.704, *p* = 0.0033), HbAS vs. HbSC (1.394 ± 0.391vs 0.863 ± 0.704, *p* = 0.0414), HbSC vs. HbAC (0.863 ± 0.704 vs. 1.432 ± 0.323, *p* = 0.0230). **(C)** CXCL10:BDNF: HbAA vs. HbSC (−0.752 ± 0.704 vs. −1.558 ± 0.603, *p* = 0.0014), HbSC vs. HbAC (−1.558 ± 0.603 vs. −0.909 ± 0.393, *p* = 0.0240). **(D)** Ang 2:Ang 1: HbAA vs. HbSS (−1.048 ± 0.400 vs. −0.5747 ± 0.230, *p* = 0.0016), HbSS vs. HbAS, (−0.5747 ± 0.230 vs. −1.130 ± 0.224, *p* = 0.0002), HbSS vs. HbAC (−0.5747 ± 0.230 vs. −0.998 ± 0.256, *p* = 0.0093), HbSS vs. HbCC (−0.5747 ± 0.230 vs. −1.372 ± 0.116, *p* = 0.0006), HbAS vs. HbSC (−1.130 ± 0.224 vs. −0.744 ± 0.465, *p* = 0.023), HbSC vs. HbCC (−0.744 ± 0.465 vs. −1.372 ± 0.116, *p* = 0.0127). **(E)** IL-6:BDNF: HbAA vs. HbSC (−2.247 ± 0.397 vs. −2.607 ± 0.372, *p* = 0.0465). **(F)** BDNF:Ang 1: HbAA vs. HbSC (−0.737 ± 0.098 vs. −0.375 ± 0.199, *p* < 0.0001) HbAS vs. HbSC (−0.744 ± 0.069 vs. −0.375 ± 0.199, *p* < 0.0001) HbSC vs. HbAC (−0.375 ± 0.199 vs. −0.749 ± 0.173, *p* < 0.0001). **(G)** Ang 2:BDNF: HbSS vs. HbAS (−0.016 ± 0.214 vs. −0.385 ± 0.246, *p* = 0.0115) HbSS vs. HbSC (−0.016 ± 0.214 vs. −0.369 ± 0.355, *p* = 0.0127), HbSS vs. HbCC (−0.016 ± 0.214 vs-0.725 ± 0.089, *p* = 0.0006). Statistical significances are indicated as *p < 0.03–0.01; **p < 0.009–0.001; ***p < 0.0002–0.00019; ****p < 0.0001 ≤ 0.00001.

Using multiple comparison tests, individual biomarker ratios between each Hb genotype were evaluated. Log-transformed relative inflammatory marker ratio values were analyzed to assess the altered interactions between inflammatory markers across sickle Hb genotypes, specifically evaluating the potential of CXCL10, BDNF, Ang 2, and IL-6 to predict the risk of complications in HbSS individuals compared to healthy controls. The ratios were compared across different Hb genotypes (Figure 4). For the CXCL10:Ang 2 ratio, significant differences were observed between HbAA and HbSC, with HbAA > HbSC (Figure 4A). CXCL10:TNF-α ratio shows significant differences between HbAA and HbSC, and between HbAS and HbSC, in the order HbAA > HbAS > HbAC > HbSC > HbSS > HbCC (Figure 4B). Additionally, for the CXCL10:BDNF ratio, significant differences were noted between HbAA and HbSC, as well as between HbSC and HbAC, with the ratios following the order HbAA > HbAS > HbAC > HbSS > HbSC > HbCC (Figure 4C). In the Ang 2:Ang 1 ratio, significant differences were observed in HbSS compared to HbAA, HbAS, HbAC, and HbCC, with significantly higher ratios following the order HbSC > HbSS > HbAC > HbAS > HbAA > HbCC (Figure 4D). Further, the IL-6:BDNF ratio showed significant differences between HbAA and HbSC, with higher ratios in the order HbAA > HbAS > HbAC > HbSS > HbSC > HbCC (Figure 4E). BDNF:Ang 1 ratio showed significant differences between HbAA and HbSC, HbAS and HbSC, and HbSC and HbAC, with higher levels in the order HbSS > HbSC > HbAC > HbAS > HbCC > HbAA (Figure 4F). For the Ang 2:BDNF ratio, significant differences were found between HbSS and HbAS, as well as between HbSS and both HbSC and HbCC, with higher levels in the order HbSS > HbSC > HbA > HbAC > HbA > HbCC (Figure 4G). The observed differences in biomarker ratios such as Ang:2 Ang 1, BNDF:Ang 1 and Ang 2:BDNF across the sickle Hb genotypes with HbSC often displaying higher biomarker ratios than HbSS, HbAA, HbAS, HbAC and HbCC suggests that HbSC patients may also experience significant vascular issues despite their overall clinical presentation being less severe than HbSS individuals. This may imply that monitoring these biomarker ratios could provide valuable insight into the risk of complications, enabling early intervention to prevent severe inflammatory responses.

### Heme and scavenger ratios differ across children with different sickle Hb genotypes

The ratios of heme and scavenger (HO-1, Hp, Hpx) plasma concentrations were analyzed to evaluate the interactions between heme and its scavengers across different sickle Hb genotypes. Supplementary Table S4; Supplementary Figure S1 illustrate the heme-to-scavenger ratios, showing significant variations across the Hb genotypes. Heme: HO-1 ratio showed significant differences between HbAA and HbSC (*p* = 0.0003), HbSS and HbSC (*p* < 0.0001), HbAS and HbSC (*p* = 0.0004), as well as HbSC and HbAC (*p* < 0.0001), HbSC and HbCC (*p* = 0.0062). HbSC showed higher heme relative to HO-1 compared to the other genotypes (Supplementary Figure S1A). For Heme: Hp ratio, significant differences were found between HbSS and HbAA, HbSS and HbAS, HbSS and HbAC, as well as between HbAA and HbSC, HbSC and HbAC (all *p* < 0.0001). HbSS and HbSC exhibited higher ratios, indicating reduced scavenging capacity of Hp in these genotypes (Supplementary Figure S1B). Additionally, in the Heme: Hpx ratio, significant differences occurred between HbAA and HbSS (*p* = 0.0031), HbAA and HbSC (*p* < 0.0001), HbSS and HbAS (*p* = 0.0048), HbSS and HbAC (*p* = 0.0086), HbAS and HbSC (*p* < 0.0001), HbSC and HbAC (*p* = 0.0001), and HbSC and HbCC (*p* = 0.0049) (Supplementary Figure S1C). HbSS and HbSC had higher heme relative to Hpx, reflecting the increased heme burden in these genotypes. The HO-1: Hpx and HO-1: Hp ratios showed significant differences between HbSS and HbAA (*p* < 0.0001), HbSC and HbAA (*p* = 0.0019), HbSC and HbAS (*p* = 0.0060), HbSC and HbAC (*p* = 0.0289), and between HbSS and both HbAA and HbCC, HbSC and both HbAS and HbAC (all *p* < 0.0001) respectively. HbSC and HbSS had higher ratios than other genotypes, indicating an increased oxidative stress response with lower Hp and Hpx availability (Supplementary Figure S1D–E). Hp:Hpx ratio showed increases in the following order: HbAA > HbAC > HbAS > HbSC > HbSS > HbCC, with significant increases in HbAA and HbSS (*p* = 0.0031), HbAA and HbAC (*p* = 0.0231) and HbSC and HbCC (*p* = 0.0219) also indicating reduced scavenging proteins in the SCD group compared to the other genotypes (Supplementary Figure S1F).

### ROC curve analysis of circulating CXCL10, BDNF, Ang 1, Ang 2, IL-6, and TNF-α levels across different sickle Hb genotypes

**FIGURE 5:**
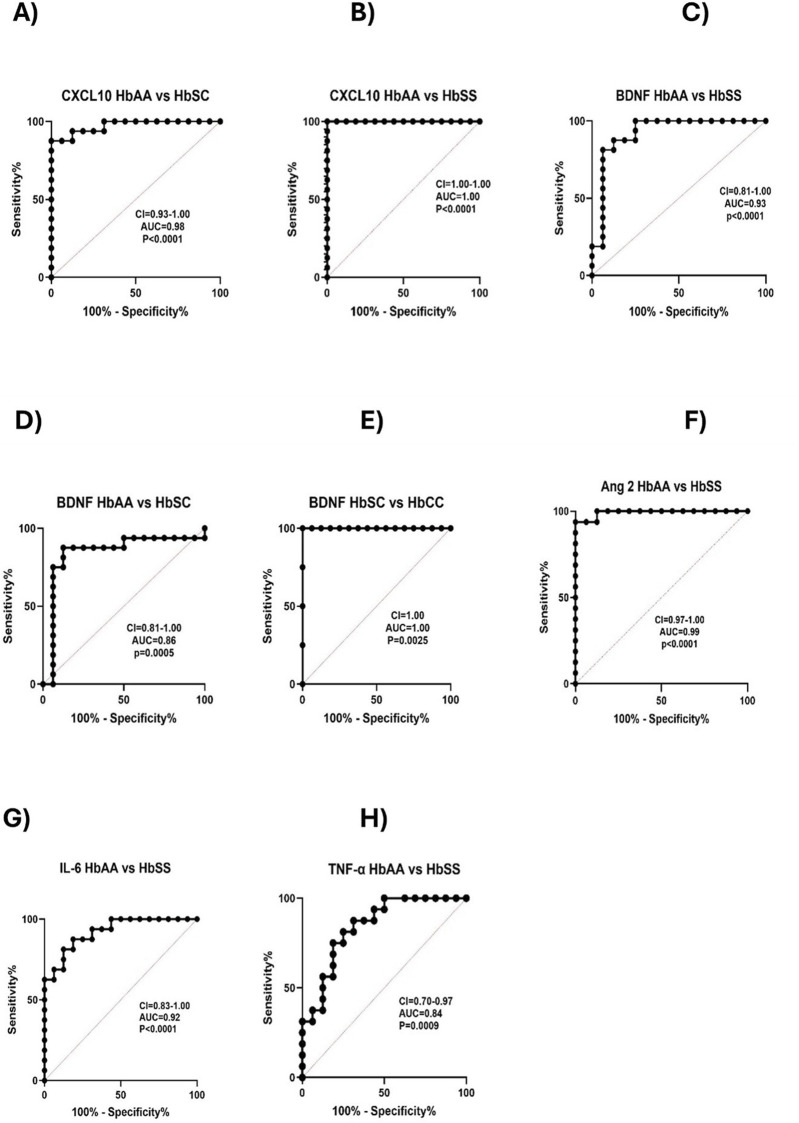
Predictive value of Circulating CXCL10, BDNF, Ang 1, Ang 2, IL-6, and TNF-α across Different Sickle Hb Genotypes. ROC analysis and its AUC were constructed to explore the usefulness of CXCL10, BDNF, Ang 1, Ang 2, IL-6, and TNF-α as potential biomarkers for predicting or as indicators of disease complications based on sickle status. Individual biomarkers independently discriminated between different Hb genotypes. The ROC plot indicated that these markers were good biomarkers: **(A)** CXCL10 between HbAA vs. HbSC (*p* < 0.0001, AUC = 0.98). **(B)** CXCL10: HbAA vs. HbSS (*p* < 0.0001, AUC = 1). **(C)** BDNF: HbAA vs. HbSS (*p* < 0.0001, AUC = 0.93). **(D)** BDNF: HbAA vs. HbSC (*p* = 0.0005, AUC = 0.86). **(E)** BDNF: HbSC vs. HbCC (*p* = 0.0025, AUC = 1. **(F)** Ang 2: HbAA vs. HbSS (*p* < 0.0001, AUC = 0.99). **(G)** IL-6: HbAA vs. HbSS (*p* < 0.0001, AUC = 0.92). **(H)** TNF-α: HbAA vs. HbSS (*p* = 0.0009, AUC = 0.84). The ROC analysis showed that CXCL10, BDNF, Ang 2, and IL-6 were effective in discriminating between different sickle Hb genotypes, with AUC values ranging from 0.86 to 1.0, indicating strong potential as predictive biomarkers for inflammatory risk and vascular complications in SCD.

Receiver Operating Characteristic (ROC) curve analysis was conducted to assess the effectiveness of circulating CXCL10, BDNF, Ang 1, Ang 2, IL-6, and TNF-α as potential biomarkers for distinguishing between different sickle Hb genotypes. The Area Under the Curve (AUC) values quantified each biomarker’s ability to differentiate between sickle Hb subgroups based on distinct biomarker profiles, with AUC values between 0.8 and 1.0 indicating strong discriminatory power. CXCL10 effectively discriminated between HbAA vs. HbSC (AUC = 0.98), HbAA vs. HbSS (AUC = 1.0), and HbAA vs. HbAS (AUC = 0.96) (Figures 5A, B). BDNF showed good discriminatory ability between HbAA vs. HbSS (AUC = 0.93), HbAA vs. HbSC (AUC = 0.86), and HbSC vs. HbCC (AUC = 1.0) (Figures 5C–E). Ang 2 also demonstrated a strong separation between HbAA vs. HbSS (AUC = 0.99) (Figure 5F). IL-6 discriminated between HbAA vs. HbSS with an AUC of 0.92 (Figure 5G), while TNF-α showed moderate discriminatory power between HbAA vs. HbSS (AUC = 0.84) (Figure 5H). CXCL10, BDNF, Ang-2, and IL-6 showed high AUC values (≥0.90), demonstrating strong discriminatory power between Hb genotypes, especially for HbSS, HbSC, and HbAA. These markers could serve as effective biomarkers for assessing disease complications and inflammatory status in SCD. The high AUC for CXCL10 and IL-6 suggests their potential to identify inflammatory states and distinguish SCD patients from healthy individuals. For example, the ROC curve analysis showed that a plasma BDNF level of 2,100 pg/mL had 87.5% sensitivity and specificity in predicting complications in SCA, while Ang-2 levels of 8,845 pg/mL were 90% sensitive and 80% specific for predicting vascular complications. However, relying on a single marker may not be sufficient to fully predict the risk of SCD complications.Elevated levels of these markers may correlate with increased risks of complications, such as vaso-occlusive crises or chronic inflammation. Similarly, high AUC values for BDNF and Ang-2 indicate their role in detecting vascular instability and neuroinflammatory responses, which are common in SCD.

### ROC curve analysis of proinflammatory and vascular injury markers for predicting risk of complications in individuals with SCD

**FIGURE 6:**
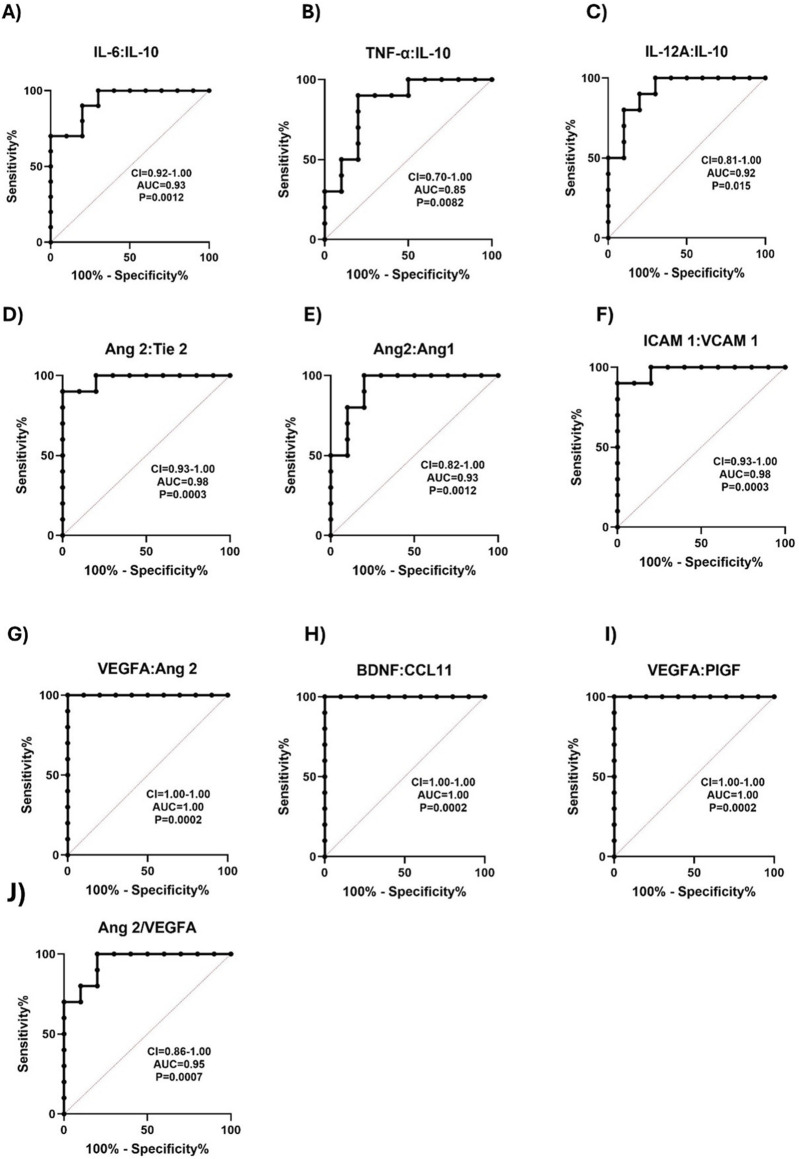
ROC curve for Proinflammatory, Vascular, and Neurotrophic Marker Ratios Distinguishes SCD from Controls. ROC analyses were plotted to explore the diagnostic power of inflammatory mediators, vascular factors, and neurotrophic factors as indicators of SCD complications from healthy controls. The ROC plot indicated that these markers were potential biomarkers for predicting SCD crisis: **(A)** IL-6:IL-10: HbAA vs. HbSS AUC = 0.93, *p* = 0.0012. **(B)** TNF-α:IL-10 HbAA vs. HbSS AUC = 0.85, *p* = 0.0082. **(C)** IL-12A:IL-10: HbAA vs. HbSS AUC = 0.92, *p* = 0.0015. **(D)** Ang 2: Tie 2: HbAA vs. HbSS AUC = 0.98 *p* = 0.0003. **(E)** Ang 2 vs. Ang 1 HbAA vs. HbSS AUC = 0.93, *p* = 0.0012. **(F)** ICAM 1: VCAM 1: HbAA vs. HbSC AUC = 0.98, *p* = 0.0003. **(G)** VEGFA: Ang 2: HbAA vs. HbSS AUC = 1.00, *p* = 0.0002. **(H)** BDNF: CCL11: HbAA vs. HbSS AUC = 1.00, *p* = 0.0002. **(I)** VEGFA: PIGF: HbAA vs. HbSS AUC = 1.00, *p* = 0.0002. **(J)** Ang 2: VEGFA: AUC = 0.96, *p* = 0.0007). The AUC for most markers of SCD relative to healthy control individuals was between 0.85–1.00. It shows that these biomarkers were not only sensitive in discriminating between SCD and healthy control but were useful markers in predicting the risk of SCD complications.

ROC curves of ratios of proinflammatory and vascular injury markers were assessed between SCD individuals and control groups, to explore the discriminatory capability of these ratios between groups. IL-6:IL-10 ratio significantly differentiated between HbAA and HbSS with an AUC = 0.93 (Figure 6A). TNF-α:IL-10 ratio differed between HbAA and HbSS with an AUC = 0.85 (Figure 6B). IL-12A:IL-10 ratio effectively discriminated between HbAA and HbSS, with an AUC = 0.92 (Figure 6C). Furthermore, Ang 2: Tie 2 ratio, differentiated between HbAA and HbSS, with an AUC = 0.98 (Figure 6D). Ang 2:Ang 1 ratio excellently discriminated between HbAA and HbSS with an AUC = 0.93 (Figure 6E). Vascular markers, ICAM 1:VCAM 1 ratio differentiated between HbAA and HbSC, with an AUC = 0.98 (Figure 6F). VEGFA:Ang 2 ratio also differentiated between HbAA and HbSS excellently, with an AUC = 1.00 (Figure 6G). BDNF:CCL11 ratio, strongly differentiated between HbAA and HbSS, with an AUC = 1.00 (Figure 6H). VEGFA:PIGF ratio, excellently differentiated between HbAA and HbSS, with an AUC = 1.00 (Figure 6I). Finally, Ang 2:VEGFA effectively discriminated between HbAA and HbSS with an AUC of 0.95 (Figure 6J). These results suggest that several neuroinflammatory and vascular injury marker ratios show strong discriminatory power between individuals with SCD (specifically HbSS and HbSC) and healthy controls (HbAA). Ratios such as IL-6:IL-10, TNF-α:IL-10 and IL-12A:IL10 ratios show strong discriminatory power, highlighting their potential to detect heightened inflammatory responses in SCD, particularly between HbSS and healthy controls. Ratios of angiogenesis markers like Ang-2 involving Ang-2:Tie 2, Ang-2:Ang 1 and VEGFA:Ang 2 also demonstrate excellent discrimination, indicating their role in assessing vascular instability in SCD. Inflammatory maker ratios such as BSNF:CCL11, VEGFA:PIGF and ICAM 1:VCAM 1, which achieved AUC values of 1.0 signify an ideal biomarker, which further emphasizes their potential for distinguishing SCD patients from controls, particularly in relation to neuroinflammatory and vascular complications.

### ROC analysis of individual inflammatory marker ratios among all Hb genotypes

ROC curves of ratios of individual circulating biomarkers were assessed and compared across the Hb genotypes, and their ability to discriminate between the groups was explored. The CXCL10:TNF-α ratio effectively distinguished between HbAA and HbSS (AUC = 0.80) (Supplementary Figure S2A), while the CXCL10:BDNF ratio showed a stronger differentiation between the same groups (AUC = 0.82) (Supplementary Figure S2B). Additionally, the Ang 2:BDNF ratio had a high discriminatory ability between HbSC and HbCC (AUC = 0.95) and also differentiated HbAA from HbSS (AUC = 0.86) (Supplementary Figure S2C, D). The IL-6:BDNF ratio showed moderate differentiation between HbAA and HbSS (AUC = 0.74) and HbAA and HbSC (AUC = 0.77) (Supplementary Figure S2E, F). Similarly, the IL-6:TNF-α ratio moderately discriminated between HbAA and HbSS (AUC = 0.75) (Supplementary Figure S2G). The BDNF:Ang 1 ratio showed excellent discriminatory power between HbSC and HbCC (AUC = 0.94) and HbAA and HbSC (AUC = 0.98) (Supplementary Figure S2H, I). Ratios like CXCL10:TNF-α and BDNF:CXCL10, with AUCs above 0.80, indicate moderate power in distinguishing HbAA from HbSS, reflecting inflammatory status. Ratios of Ang-2:BDNF, with high AUC values (0.95), suggests its relevance in identifying vascular dysregulation, especially in differentiating between HbSC and HbCC or HbSS from HbAA. While the IL-6:BDNF and IL-6:TNF-α ratios showed moderate discriminatory ability with AUCs of 0.74 and 0.75 respesctively, they may still be useful in assessing inflammatory imbalances in SCD. The BDNF: Ang 1 ratio, with high AUC values (0.98), indicates its potential as a sensitive marker for distinguishing genotypes based on neurotrophic and vascular factors.

### ROC analysis of biomarker to scavenger ratio among all sickle Hb genotypes

The ROC analysis of biomarker-to-scavenger ratios demonstrated significant discriminatory power between various sickle Hb genotypes, indicating their potential utility for diagnostic and prognostic applications.

CXCL10:HO-1 ratio exhibited strong discrimination between HbAA and HbSC, with an AUC of 0.93 (Supplementary Figure S3A). Similarly, this ratio significantly differentiated HbAA from HbSS, also with an AUC of 0.93 (Supplementary Figure S3B). CXCL10:Hpx ratio showed moderate discrimination between HbAA and HbSS, with an AUC of 0.75 (Supplementary Figure S3C).

Similarly, the CXCL10: Hp ratio exhibited perfect discrimination between HbAA and HbSS, with an AUC of 1.00, indicating an ideal biomarker for distinguishing these genotypes (Supplementary Figure S3D). The Ang 2:HO-1 ratio differentiated HbAA from HbSC and HbSS, with an AUC of 0.75 for both comparisons, indicating moderate discriminatory power (Supplementary Figure S3E, F). Further, the Ang 2: Hpx ratio effectively discriminated between HbAA and HbSC, and also between HbAA and HbSS, with an AUC of 0.90 for both comparisons, indicating strong discrimination (Supplementary Figure S3G, H). Ang 2:Hp ratio demonstrated perfect discrimination between HbAA and HbSS, with an AUC of 1.00, indicating its strong potential as an ideal biomarker (Supplementary Figure S3I). The BDNF: HO-1 ratio showed significant discrimination between HbSC and HbCC, as well as between HbAA and HbSS, both with an AUC of 0.88, indicating good discriminatory power (Supplementary Figure S3J, K). BDNF: Hpx Ratio effectively distinguished between HbAA and HbSC (AUC = 0.95) and also demonstrated strong discrimination between HbAA and HbSS (AUC = 0.99) (Supplementary Figure S3L, M). BDNF:Hp ratio significantly discriminated between HbAA and HbSS, with an AUC of 0.91, showing strong discrimination (Supplementary Figure S3N). The biomarker-to-scavenger ratios demonstrate significant discriminatory capabilities between various sickle Hb genotypes, with some ratios achieving perfect separation between HbAA and HbSS. These findings suggest that these ratios have potential diagnostic and prognostic applications that can be applied in clinical settings to differentiate between sickle Hb subgroups, assess disease complications, and guide personalized management strategies for sickle cell disease (SCD) patients.

### ROC analysis of neuroinflammatory/vascular markers in SCD and control individuals

ROC curves were constructed to evaluate the diagnostic accuracy of various neuroinflammatory and vascular markers in distinguishing between individuals with HbSS and healthy HbAA controls, focusing on the sensitivity and specificity of the markers of interest. CCL11 demonstrated a high discriminative capacity with an AUC of 0.95, *p* = 0.0007 (Supplementary Figure S4A). IL-8 showed moderate discrimination between the groups with an AUC of 0.74 and *p* = 0.06, but the result was not statistically significant (Supplementary Figure S4B). VEGFA exhibited an AUC of 0.82 and *p* = 0.015, indicating a strong ability to distinguish between the two groups (Supplementary Figure S4C). Other markers, including ICAM-1 (AUC = 0.90, *p* = 0.0025), VCAM-1 (AUC = 1.00 and *p* = 0.0002), Flt-1 (AUC = 0.98 and *p* = 0.0003), PIGF (AUC = 0.96 and *p* = 0.0005), VEGF-D (AUC = 0.84, and *p* = 0.01), bFGF (AUC = 0.86 and *p* = 0.006), and MDC (AUC = 0.96 and *p* = 0.0005), also demonstrated excellent discriminatory power (Supplementary Figure S4D–J). There was no significant discrimination by IL-10 (AUC = 0.73), suggesting that it may not be a useful marker for distinguishing between HbSS and HbAA individuals (Supplementary Figure S4K). The ROC analysis highlights several markers with high sensitivity and specificity in differentiating between SCD (HbSS) patients from healthy (HbAA) controls. In particular, CCL11, ICAM-1, VCAM-1, Flt-1, PIGF, and MDC showed high diagnostic accuracy and could be valuable for prognosis and risk assessment of complications associated with SCD.

### Ratios of angiogenic, chemokine, and vascular injury markers between sickle Hb group and healthy controls

Ratios of Ang 2, VEGFA, Tie 2, CCL11, IL-12p40, ICAM-1, and VCAM-1 were analyzed between SCD patients and healthy controls to determine their potential for predicting complications in SCD. Significant differences were observed in the plasma concentration ratios of key angiogenic and inflammatory markers between SCD (HbSS) patients and healthy controls (HbAA). The ratio of Ang 2:VEGF was significantly elevated in HbSS patients compared to HbAA controls (*p* = 0.0001), indicating increased vascular instability and angiogenic imbalance in SCD (Supplementary Figure S7A). A significant increase in the ratio Ang 2:Tie 2 (*p* = 0.0002) suggests a disruption in endothelial stability and angiogenic signaling, key contributors to the pathophysiology of SCD (Supplementary Figure S7B). The CCL11:IL-12p40 ratio showed a moderately significant mean increase in HbSS patients compared to controls (0.7090 ± 0.437 vs. 0.4030 ± 0.142, *p* = 0.049), highlighting an inflammatory imbalance potentially linked to the immune dysregulation in SCD (Supplementary Figure S7C). For VCAM-1:ICAM-1, HbSS patients exhibited a significant elevation in the VCAM-1:ICAM-1 ratio (2.479 ± 1.052 vs. 0.5455 ± 0.151, *p* < 0.0001), which may reflect heightened endothelial activation, adhesion, and potential for vaso-occlusion in SCD (Supplementary Figure S7D) and Ang 2:Ang 1 ratio was significantly higher in HbSS patients (*p* = 0.0011), further supporting the evidence of endothelial dysfunction and vascular instability in SCD (Supplementary Figure S7E). These findings suggest that these ratios are useful biomarkers for assessing endothelial dysfunction, angiogenic imbalance, and inflammatory status in SCD. The significant differences between HbSS and HbAA in these ratios underscore their potential clinical utility in predicting vascular complications, such as vaso-occlusive crises, and monitoring disease progression in SCD patients.

### Comparison of inflammatory mediators between SCD and control groups

**FIGURE 7:**
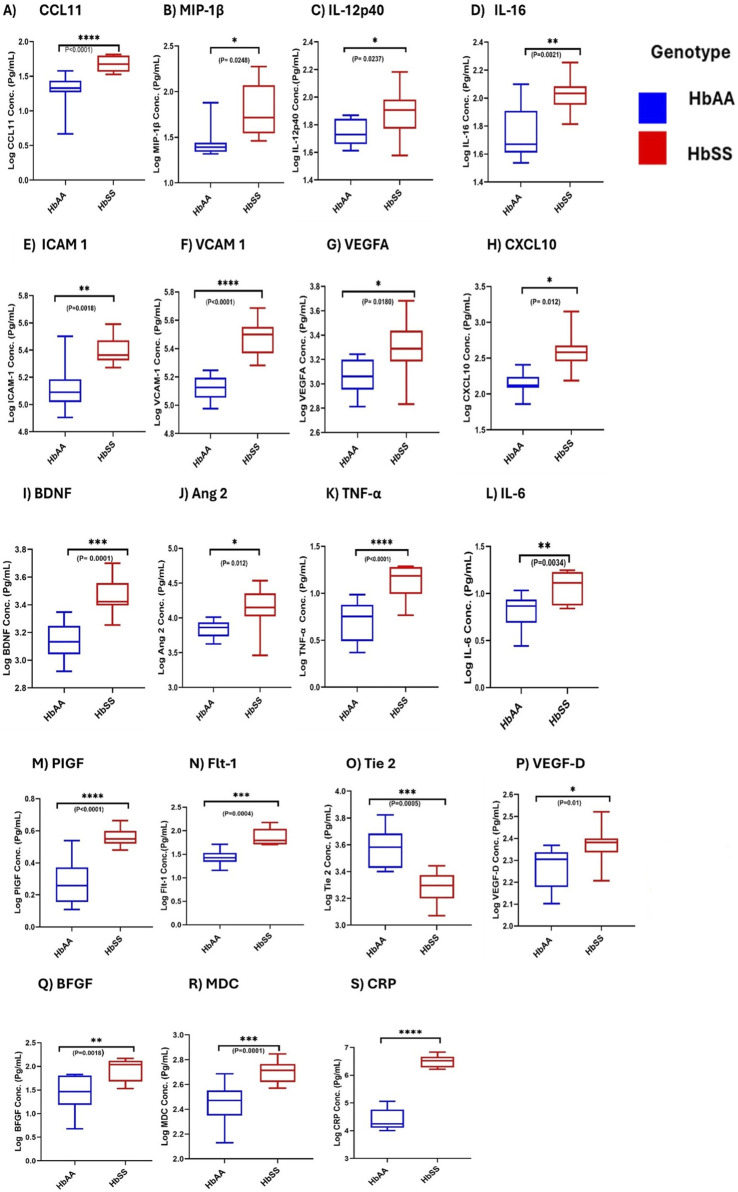
Comparative Analysis of Inflammatory, Angiogenic, and Adhesion Marker Levels in SCD and Healthy Controls. Independent sample t-test assessed the comparisons of individual neuroinflammatory markers between SCD individuals (HbSS) and the control (HbAA). Box and whisper graph plots showing minimum and maximum mean plasma values. Significant increases/decreases were observed in SCD than healthy control in: **(A)** CCL11: *p* < 0.0001. **(B)** MIP-1β: *p* = 0.0248. **(C)** IL-12p40:*p* = 0.0237. **(D)** IL-16:*p* = 0.0021. **(E)** ICAM 1: *p* = 0.0018. **(F)** VCAM 1: *p* < 0.0001. **(G)** VEGFA: *p* = 0.018. **(H)** CXCL10: *p* = 0.012. **(I)** BDNF: *p* = 0.0001. **(J)** Ang 2: *p* = 0.012. **(K)** TNFα:*p* < 0.0001. **(L)** IL-6:*p* = 0.0034. **(M)** PIGF: *p* < 0.0001. **(N)** Fit-1: *p* = 0.0004. **(O)** Tie-2: *p* = 0.0005. **(P)** VEGF-D: *p* = 0.0111. **(Q)** BFGF:*p* = 0.0018. **(R)** MDC: *p* = 0.0001. **(S)** CRP: *p* < 0.0001. Statistical significances are indicated as *p < 0.03–0.01; **p < 0.009–0.001; ***p < 0.0002–0.00019; ****p < 0.0001 ≤ 0.00001.

Comparisons of plasma mean levels of inflammatory markers, including proinflammatory, angiogenic mediators, and vascular markers, were conducted to assess their potential in predicting complications in individuals with Sickle Cell Disease (SCD) compared to controls (Figure 7). Chemokines CCL11 (Eotaxin), CXCL10 and MDC showed significantly higher levels in HbSS than HbAA, with plasma mean levels of CCL11(49.2 ± 13.26 vs. 22.6 ± 9.7 pg/mL, *p* < 0.0001), CXCL10 (463.2 ± 353.8 vs. 148.9 ± 54.8 pg/mL, *p* = 0.0125) and MDC (512.1 ± 106.8 vs. 292.7 ± 97.5 pg/mL, *p* = 0.0001) respectively (Figures 7A, H, R). MIP-1β levels was significant higher in HbSS than in HbAA (*p* = 0.0248) (Figure 7B). Plasma mean vascular injury markers ICAM-1 and VCAM-1 levels were significantly elevated in HbSS than in HbAA (258,584 ± 73,023 vs. 143,135 ± 68,405 pg/mL, *p* = 0.0018) and (308,364 ± 90,039 vs. 134,067 ± 25,680 pg/mL, *p* < 0.0001) respectively, suggesting increased vascular inflammation and injury in SCD (Figures 7E, F). Plasma mean cytokines TNF-α, IL-12p40, IL-16 and IL-6 levels were significantly higher in HbSS (14.3 ± 4.7 vs. 5.6 ± 2.3 pg/mL, *p* < 0.0001), (83.9 ± 33.7 vs. 55.8 ± 11.9 pg/mL, *p* = 0.023) (111.0 ± 31.23 vs. 61.7 ± 30.2 pg/mL, *p* = 0.0021) and (12.38 ± 4.50 vs 6.84 pg/mL, *p* = 0.0034) indicating increased proinflammatory activity compared to controls respectively (Figures 7C, D, K, L). For Angiogenic markers, significant plasma mean differences were found in VEGFA, PIGF, Flt-1, VEGF-D, Ang 2 and bFGF, with higher levels in HbSS compared to HbAA. VEGFA levels were higher in HbSS (238.1 ± 43.6 pg/mL) than in HbAA (185.9 ± 38.6 pg/mL, *p* = 0.018). PIGF levels were higher in HbSS (36.5 ± 4.9 pg/mL) than in HbAA (19.6 ± 6.7 pg/mL, *p* < 0.0001). Flt-1 levels were higher in HbSS (78.8 ± 35.6 pg/mL) than in HbAA (28.1 ± 10.2 pg/mL, *p* = 0.0004). Additionally, VEGF-D levels were significantly higher in HbSS (2,188 ± 1,137 pg/mL) than in HbAA (205 ± 367.7 pg/mL, *p* = 0.01), Ang 2 levels higher in HbSS than HbAA (16,110 ± 8,703 vs. 7,153 ± 1,954 pg/mL, *p* = 0.012), BDNF levels were also higher in HbSS than in HbAA (2,789 ± 902.3 vs. 1437 ± 440.6 pg/mL, *p* = 0.0004) and bFGF levels were higher in HbSS than in HbAA (92.3 ± 42.8 vs. 35.5 ± 23.82 pg/mL, *p* = 0.0018). Plasma mean Tie-2 levels were, however, lower in HbSS (2,009 ± 512.2 pg/mL) than in HbAA (3,988 ± 1,398 pg/mL, *p* = 0.0005), suggesting dysregulated angiogenesis in SCD (Figures 7G, I, J, M–Q). Systemic inflammatory marker CRP levels were significantly elevated in HbSS than in HbAA (3,537,499 ± 1,697,120 vs. 36,587 ± 11,647 pg/mL, *p* < 0.0001), further highlighting the heightened inflammatory response (Figure 7S). These results highlight several key inflammatory mediators that are significantly altered in individuals with SCD, suggesting an increased inflammatory burden, vascular injury, and dysregulated angiogenesis compared to controls. Elevated levels of TNF-α, VCAM-1, VEGFA, and CRP suggest a heightened risk of complications such as vaso-occlusive crises. Monitoring these markers could provide valuable information for risk assessment and help guide targeted therapeutic interventions.

### Cytokine concentration correlates with complete blood count (Hb, WBC, and RBC) in individuals with different sickle Hb genotypes

**FIGURE 8:**
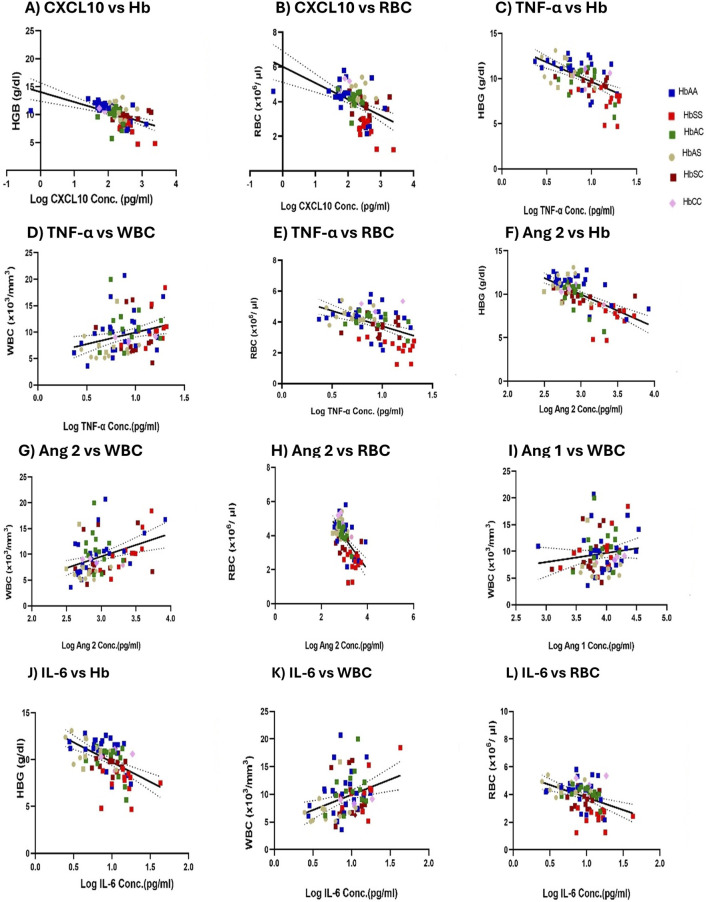
Mean plasma Inflammatory Marker Concentrations Correlate with Hb levels, WBC, and RBC counts Across sickle cell Genotypes. A two-tailed Pearson correlation with a 95% confidence interval and a linear regression was used for all comparisons and shown as log-transformed. Squares with different colors represent individuals’ HB levels, WBC, and RBC counts correlated with mean plasma concentrations of biomarkers, including a solid line of best fit and a dotted 95% confidence bands. Specific correlations were observed in: **(A)** CXCL10 vs. Hb: R2 = 0.1780, *p* < 0.0001, and Y = −1.788*X + 14.04. **(B)** CXCL10 vs. RBC: *R* ^2^ = 0.1620, *p* = 0.0002 and Y = −0.9413*X + 6.018. **(C)** TNF-α vs. Hb: *R* ^2^ = 0.351, *p* < 0.0001 and Y = −4.334*X + 13.96. **(D)** TNF-α vs. WBC: *R* ^2^ = 0.1342, *p* = 0.0008 and Y = 4.412*X + 5.519. **(E)** TNF-α vs. RBC: *R* ^2^ = 0.2586, *p* < 0.0001 and Y = −1.989*X + 5.714. **(F)** Ang 2 vs. Hb: *R* ^2^ = 0.283, *p* < 0.0001 and Y = −3.752*X + 21.24. **(G)** Ang 2 vs. WBC: *R* ^2^ = 0.223, *p* < 0.0001 and Y = 4.464X - 3.815. **(H)** Ang 2 vs. RBC: *R* ^2^ = 0.245, *p* < 0.0001 and Y = −1.889X + 9.552. **(I)** Ang 1 vs. WBC: *R* ^2^ = 0.1307, *p* = 0.0001 and Y = 1.710X + 2.857. **(J)** IL-6 vs. Hb: *R* ^2^ = −0.252 *p* < 0.0001 and Y = −4.211*X + 13.95. **(K)** IL-6 vs. WBC: *R* ^2^ = 0.229, *p* < 0.0001 and Y = 5.521*X + 4.388. **(L)** IL-6 vs. RBC: *R* ^2^ = −0.1732, *p* = 0.0001 and Y = −1.809*X + 5.590.

The correlation analysis revealed no significant relationships between the inflammatory and platelet levels. However, several significant correlations were observed between specific biomarkers and complete blood counts (CBC) (Figure 8). CXCL10 showed a significant positive correlation with Hb levels (*r* ^2^ = 0.178, *p* < 0.0001) (Figure 8A) and RBC counts (*r* ^2^ = 0.162, *p* = 0.0002) (Figure 8B). TNF-α demonstrated a significant positive correlation with Hb levels (*r* ^2^ = 0.351, *p* < 0.0001) (Figure 8C) and WBC counts (*r* ^2^ = 0.134, *p* = 0.0008) (Figure 8D), while it was negatively correlated with RBC counts (*r* ^2^ = −0.259, *p* < 0.0001) (Figure 8E). Similarly, plasma Ang 2 levels had a significant negative correlation with Hb levels (*r* ^2^ = −0.283, *p* < 0.0001) (Figure 8F) and RBC counts (*r* ^2^ = −0.245, *p* < 0.0001) (Figure 8G) and a significant positive correlation with WBC counts (*r* ^2^ = 0.223, *p* < 0.0001) (Figure 8H). Additionally, plasma Ang 1 levels correlate positively with WBC counts (*r* ^2^ = 0.1307, *p* = 0.0001) (Figure 8I). IL-6 was significantly and negatively correlated with Hb levels (*r* ^2^ = −0.252, *p* < 0.0001) (Figure 8J) and RBC counts (*r* ^2^ = −0.173, *p* = 0.0001) (Figure 8K), while it showed a significant positive correlation with WBC counts (*r* ^2^ = 0.229, *p* < 0.0001) (Figure 8L).

Further analysis revealed genotype-specific correlations between CXCL10, TNF-α, Ang 2, BDNF, Ang 1, and IL-6 with Hb, WBC, and RBC levels (Supplementary Table S6–S8). Among only the HbSS group, plasma CXCL10 and IL-6 levels were significantly and negatively correlated with Hb levels (*r* ^2^ = −0.7402, *p* = 0.0010 and *r* ^2^ = −0.7537, *p* = 0.0019) and For HbAA genotype, CXCL10 was positively correlated with Hb levels (*r* ^2^ = 0.524, *p* = 0.0371) (Supplementary Table S6). Similarly, plasma BDNF and Ang 2 levels positively correlated with WBC counts in the HbSS group (*r* ^2^ = 0.8397, *p* = 0.0001 and *r* ^2^ = 0.4894, *p* = 0.0453), while plasma CXCL10 negatively correlated with WBC counts (*r* ^2^ = −0.6272, *p* = 0.0093) (Supplementary Table S7). Finally, plasma BDNF, TNF-α, Ang1 and Ang 2 levels all showed a significant positive correlation with RBC counts in the HbSS group (*r* ^2^ = 0.5445, *p* = 0.0292, *r* ^2^ = 0.5737, *p* = 0.0438, *r* ^2^ = 0.6329, *p* = 0.0085 and *r* ^2^ = 0.8358, *p* < 0.0001), indicating a strong positive association (Supplementary Table S8).

For HbAS Individuals, BDNF was positively correlated with Hb (*r* ^2^ = 0.623, *p* = 0.0174). TNF-α was negatively correlated with Hb (*r* ^2^ = −0.755, *p* = 0.0018). HbAC Individuals: TNF-α was negatively correlated with Hb (*r* ^2^ = −0.590, *p* = 0.0264) and also with WBC (*r* ^2^ = −0.666, *p* = 0.0094). IL-6 also showed a negative correlation with Hb (Supplementary Table S6). In HbAA Individuals, CXCL10 was positively correlated with Hb (*r* ^2^ = 0.524, *p* = 0.0371). In HbAC Individuals, BDNF was positively correlated with WBC in HbAC (*r* ^2^ = 0.790, *p* = 0.0008), but BDNF showed a negative correlation with RBC in HbAS (*r* ^2^ = −0.623, *p* = 0.0173), and HbCC (*r* ^2^ = −0.955, *p* = 0.0445) (Supplementary Table S8). The significant positive correlations of TNF-α and IL-6 with WBC counts suggest an association between these pro-inflammatory markers and an elevated immune response in individuals with SCD. The negative correlations between CXCL10 and IL-6 with Hb indicate potential links to anemia and hemolysis in SCD patients, suggesting that higher levels of these biomarkers are associated with worse hematological outcomes. Genotype-specific correlations observed between biomarkers and CBC parameters provide insights into the differential regulation of inflammation and blood cell parameters in SCD. Positive correlations of BDNF, TNF-α, Ang 1 and Ang 2 with RBC counts in HbSS individuals indicate their involvement in maintaining erythropoiesis and vascular health in response to disease severity, suggesting a compensatory or disease-specific processes interacting with inflammation. Conversely, negative correlations between TNF-α and Hb/WBC levels in HbAS and HbAC individuals suggest an inhibitory effect of inflammation on hematological parameters.

The study also identified significant correlations between heme and heme scavenger proteins (HO-1, Hp, and Hpx) and the CBC counts (Hb, WBC, and RBC) among specific genotypes (Supplementary Figure S5; Supplementary Table S9–S11). In the HbAC genotype, heme showed a negative correlation with Hb levels (*r* ^2^ = −0.637, *p* = 0.0143), whereas HO-1 showed a negative correlation with Hb levels (*r* ^2^ = −0.637, *p* = 0.0143) (Supplementary Table S9). Heme and HO-1 both had negative correlations with RBC counts (*r* ^2^ = −0.667, *p* = 0.0092; *r* ^2^ = −0.748, *p* = 0.0021, respectively) (Supplementary Table S11).

For HbAS genotype, HO-1 showed a negative correlation with Hb levels (*r* ^2^ = −0.798, *p* = 0.0006) and WBC counts (*r* ^2^ = −0.623, *p* = 0.0173) (Supplementary Table S9, S10). In HbSS group, Heme was positively correlated with WBC counts (*r* ^2^ = 0.865, *p* = 0.0495) but Hpx showed a negative correlation with WBC counts (*r* ^2^ = −0.518, *p* = 0.0400). For HbSC genotype, HO-1 showed a negative correlation with Hb levels (*r* ^2^ = −0.653, *p* = 0.0061) and Hpx was positively correlated with RBC counts (*r* ^2^ = 0.681, *p* = 0.0037). Hpx was positively correlated with WBC counts (*r* ^2^ = 0.711, *p* = 0.0020), and Hp showed a positive correlation with RBC counts (*r* ^2^ = 0.506, *p* = 0.0020) (Supplementary Table S9–S11).

Finally, in the HbCC group, Hpx and Hp showed a negative correlation with WBC counts (*r* ^2^ = −0.956, *p* = 0.0445) and (*r* ^2^ = −0.956, *p* = 0.0445). The correlation analysis of heme, heme scavengers, and CBC parameters across different Hb genotypes reveals differential relationships that are genotype-specific, which provide important insights into the interactions between heme metabolism and hematologic responses in SCD. Positive correlations with scavengers such as Hpx and Hp in sickle Hb genotypes suggest a compensatory mechanism to maintain RBC levels, while negative correlations highlight the potential detrimental effects of heme accumulation and oxidative stress.

### Principal component analysis (PCA) of inflammatory markers, WBC, RBC, Hb, and PLT for all Hb genotypes

**FIGURE 9:**
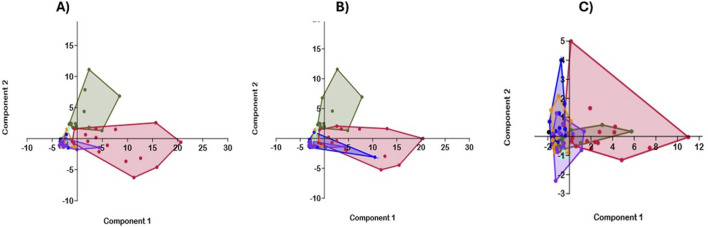
Principal Component Analysis (PCA) of Inflammatory Marker and Hematological Variables across Sickle Hb Genotypes. PCA was applied to biomarkers (BDNF, Ang 2, CXCL10, IL-6, TNF-α) and CBC (WBC, HCT, Hb, and PLT) variables. A correlation matrix was employed due to the variables being measured in various units and between-group analysis. The dark olive green represents the HbSC group; the crimson represents the HbSS group, the blue-violet represents the HbAC, and the dark orange represents the HbAS group. The blue represents the HbAA group, and the aquamarine represents the HbCC group. **(A)** Overlapping PCA of Biomarker, Heme, heme scavengers, and CBC among all genotypes. The component 1 and 2 eigenvalue and percentage variance are 24.9% and 69.0% and 5.8% and 16.2%, respectively. **(B)** PCA of scavengers and CBC among the genotypes: The PC1 and PC2 eigenvalue and percentage variance are 22.5% and 75.2% and 5.7% and 18.9%, respectively. **(C)** Displays only selected inflammatory profiles (BDNF, Ang1 Ang2, IL-6, CXCL10, TNF-α) and CBC between SCD and healthy controls. Component 1 and 2 eigenvalue and percent variance are 4.85 (57.8%) and 1.0 (12.0%), respectively.

PCA demonstrated that HbSS and HbSC formed distinct clusters, separating them from other genotypes (HbAA, HbAS, HbAC, and HbCC) (Figure 9A). The score plot indicated that HbSS and HbSC were primarily distinguished from other genotypes by the first principal component (PC1), while other Hb groups clustered within the different regions. PC1 captured the variance associated with the sickle cell Hb genotypes (HbSS and HbSC), whereas PC2 represented the remaining Hb genotypes (HbAS, HbAC and HbCC). PC1 had an eigenvalue of 24.9, explaining 69.0% of variance, while and PC2 had an eigenvalue of 5.8, accounting for 16.2% %. Similarly, in the PCA analysis of scavengers and CBC across the Hb genotypes, HbSS and HbSC again formed distinct clusters (Figure 9B). PC1, in this case, had an eigenvalue of 22.54 and explained 75.2% of variance, while PC2 explained 18.8% with an eigenvalue of 5.6. The loadings plot revealed that HO-1 and Heme showed the highest values, while Hpx and Hp were lower.

In the final analysis, which focused solely on SCD and control groups, HbSS and HbSC remained clustered together, clearly distinct from the healthy group (Figure 9C). PC1 had an eigenvalue of 154.5, explaining 63.5% of the variance and PC2 had an eigenvalue of 69.0, accounting for 28.3%. HbSS clusters were more tightly grouped and farther from the healthy control group.

## Discussion

Sickle cell disease (SCD) involves complex mechanisms such as intravascular hemolysis, recurrent vaso-occlusion, chronic vascular inflammation, and endothelial activation, leading to chronic anemia, pulmonary hypertension, and organ damage (Kato et al., 2017; Conran and Belcher, 2018; Ansari and Gavins, 2019). Traditional management focuses on acute complications and symptom relief (Ballas, 2018), but recent advances emphasize the role of specific biomarkers in improving SCD management (Salinas Cisneros and Thein, 2020). HbSS erythrocytes interact with the vascular endothelium, exacerbated by cytokines like TNF-α, IL-6, and IL-8 (Pathare et al., 2004; Makis et al., 2000).

Previous work from our lab identified inflammatory markers (CXL10, TNF-α, IL-6, IL-8) in adults with different Hb genotypes, predicting malarial and SCD severity (Harp et al., 2020). This study examines circulating biomarkers in children with different Hb genotypes, particularly SCD (HbSS and HbSC), to predict crises, guide therapy, monitor treatment, and elucidate disease mechanisms. SCD patient exhibited elevated levels of biomarkers such as BDNF, Ang 2, CXCL10, TNF-α, and IL-6) compared to control individuals (Figures 1, 7). Table 2 summarizes some of the main functions of these markers in association with SCD. The study reveals significant hematological differences across Hb groups, particularly in Hb levels, RBCs, and WBC counts. CBC values were markedly altered in the SCD (HbSS and HbSC) group compared to healthy controls (Table 1). Reduced Hb levels, hematocrit, and RBC counts in SCD patients indicate an ongoing RBC lysis, a hallmark of the hemolytic state, similar to what is observed in malaria (Kosiyo et al., 2020; Harp et al., 2021). Elevated WBC and platelet counts suggest a persistent inflammatory state, which may accelerate disease progression and has been linked to early SCD-related mortality (Platt et al., 1994). Our findings align with previous studies showing higher WBC and low RBC counts in SCA compared to control individuals (Kosiyo et al., 2020; Anyaegbu et al., 1998). Leukocytes drive inflammation, promote VOC process and are associated with complications like Acute Chest syndrome (ACS) (Castro et al., 1994), silent cerebral infarction (Kinney et al., 1999), and clinically overt stroke (Platt et al., 1994; Powars, 2000).

**TABLE 2:** Circulating Biomarkers associated with Sickle Cell Disease (SCD). This table lists biomarkers associated with SCD, their functions, and how they contribute to disease pathology and references.

Biomarkers	Function	Association in Sickle Cell Disease	References
CNS			
BDNF	Promotes cell survival, growth, and neuronal maintenance	Elevated in SCD, implicated in risk of stroke development	Chen et al. (2013), Chambliss et al. (2023)
Vascular			
Ang 1	Vascular Endothelial survival and stability. Anti-inflammatory properties that maintain vascular integrity and reduce plasma leakage. Anti-apoptotic effect on endothelial cells	Prevents vascular complications of SCD by maintaining vascular integrity	Baffert et al. (2006), Jiang et al. (2014), Harfouche et al. (2002), Koh (2013)
Ang 2	Destabilizes endothelium and promotes vascular permeability. Regulates angiogenesis and inflammation	Upregulated in response to hypoxia and inflammation common in SCD. Contribute to vascular leakage. Elevation associated with retinopathy in children with SCD	Jiang et al. (2014), Scholz et al. (2015), Andrawes et al. (2019)
Tie 2	Receptor for Ang 1 and Ang 2	Dysregulation of Tie 2 signaling contributes to endothelial dysfunction and promotes vaso-occlusion. Its suppression promotes the expression of ICAM 1 and VCAM 1	Parikh (2017), Hellenthal et al. (2022)
Flt 1	VEGF receptor induces vascular repair	Abnormal angiogenesis leads to complications like pulmonary hypertension	Niu and Chen (2010)
VEGF D	Angiogenic mediators	Elevated in SCD during imbalances in angiogenic proliferative retinopathy in HbSS individuals	Nagel et al. (2003)
VEGF	Increase permeability	Implicated in pain crises and endothelial dysfunction in SCD. Promotes inflammation and vascular permeability. Dysregulation may contribute to chronic organ damage in SCD	Jin et al. (2021)
PIGF	Angiogenic. Plays a role in angiogenesis and chronic inflammation	Upregulated in association with VEGFA and Flt1 contributes to abnormal angiogenesis and vascular permeability, leading to pulmonary hypertension in SCD. Elevated levels of PlGF may exacerbate inflammation and vaso-occlusive crises in SCD	Conran and Belcher (2018), Eddy et al. (2018), Brittain et al. (2010)
BFGF	Angiogenic mediator	Elevation in SCD contributes to proliferative vasculopathy	Niu et al. (2009)
Adhesion molecules			
VCAM 1	leukocyte and platelet tethering and adhesion molecule	Elevated levels in SCD facilitate leukocyte adhesion to the endothelium and contribute to vaso-occlusion in SCD	Santaterra et al. (2020), Belcher et al. (2010)
ICAM 1	Mediate in cell adhesion process	Elevated levels contribute to the adhesion of sickle cells to endothelial cells, promoting vaso-occlusion and ischemic damage	Belcher et al. (2010), White et al. (2020)
Proinflammatory cytokines			
TNF-α	Promotes expression of inflammatory cytokines and enhances leukocyte migration. that activates and differentiates various immune cells	TNF-α levels are elevated in SCD and contribute to chronic inflammation and pain episodes as well as endothelial cell dysfunction	Solovey et al. (2017), Gao et al. (2007)
IL-6	Induction of acute phase proteins	Increased levels in SCD may inhibit TNF-α activity and upregulate anti-inflammatory IL-10. Controls local or systemic acute inflammatory responses, associated with acute painful vaso-occlusive episodes	Pathare et al. (2004), Keikhaei et al. (2013), Xing et al. (1998)
IL-8	Neutrophil chemotaxis	Elevation contributes to the recruitment of neutrophils to the endothelium to promote vaso-occlusion, pain, and inflammation	Keikhaei et al. (2013), Makis et al. (2000)
IL-10	Regulates inflammatory responses. Potent anti-inflammatory cytokine	Limits proinflammatory cytokines	Conran and Belcher (2018), Sarray et al. (2015)
IL-16	Chemoattractant for various immune cells, Also as T cell activation	Involved in leukocyte recruitment and exacerbation of vaso-occlusive crises	Wu et al. (2023), Kaser et al. (2000)
IL-17α	Recruitment of neutrophils and macrophages	Recruitment of inflammatory cells to sites of vaso-occlusion, leading to tissue injury	Pathare et al. (2004), Makis et al. (2000)
IL-1α	Released from activated endothelial cells and amplifies the inflammatory response	Elevation in SCD induces leukocyte recruitment, endothelial cell activation and the production of other inflammatory mediators to amplify inflammatory responses in SCD with vaso-occlusive pain	Pathare et al. (2004), Driss et al. (2012), Francis and Haywood (1992), Pathare et al. (2003)
Angiostatic			
IL-12	Differentiation of naive T cells into Th1 cells is key in the adaptive immune response	May involve modulation of immune responses and potential exacerbation of inflammatory processes	Conran and Belcher (2018), Sarray et al. (2015)
CXCL10	Recruitment of leukocytes to blood vessel walls	Upregulation exacerbates vaso-occlusion crisis in SCD	Conran and Belcher (2018), Harp et al. (2020)
Proinflammatory Chemokines			
CCL11	Chemotactic agent for eosinophils to sites inflammation	Play a role in neuroinflammatory = pulmonary complications in SCD	Minniti et al. (2020)
IL-8	Chemotactic factor for neutrophils	Higher levels implicated during painful crisis in SCD	Duits et al. (1998)
MCP-1	Involved in leukocytes migration site of inflammation	Elevated in SCD during a painful crisis or steady state	Conran and Belcher (2018), Chen et al. (2020)
MIP-1β	Stimulates leukocyte proliferation and differentiation	Elevated levels in SCD contribute to inflammation and the immune response to vaso-occlusion	Qari et al. (2012)
MDC	Recruitment of dendritic cells, NK cells, and T cells Involve in immune regulation and intensifying inflammation	Recruits immune cells to sites of tissue injury, contributing to inflammation and pathophysiology of vaso-occlusive crises	Driss et al. (2012)
CRP	Acute phase protein, Indicator for Systemic Inflammation	Involve in chronic inflammatory response during SCA vaso-occlusive crisis	Mohammed et al. (2010), Krishnan et al. (2010)

Inflammatory markers, including CXCL10, BDNF, Ang 2, TNF-α, and IL-6 correlated with hematological values across the sickle Hb genotypes (Figure 7; Supplementary Table S6–S8). As noted in previous studies, these markers are potential predictors of complications in SCD and malaria complications (Chambliss et al., 2021; Harp et al., 2020; Qari et al., 2012; Wilson et al., 2011; Lekpor et al., 2022; Yeo et al., 2008). Chemokines such as CXCL10, MCP-1, and MIP-1β, which mediate leukocyte migration and inflammation, show elevated levels in malaria, reflecting disease severity (Chen et al., 2020; Wilson et al., 2011; Ioannidis et al., 2014). Specifically, upregulation of CXCL10 has been linked to increased disease severity in both malaria (Wilson et al., 2011; Nie et al., 2009) and SCD (Driss et al., 2012; Harp et al., 2020).

In SCD, CXCL10 recruits leukocytes to the vasculature, correlating with pain intensity and VOC events (Conran and Belcher, 2018). Thus, targeting CXCL10 and its receptor CXCR3 may reduce inflammation and clinical symptoms. CXCL10 is linked to various diseases, including cancer (Persano et al., 2007), and infectious diseases (Liu et al., 2011), making it a potential therapeutic target (Dickinson-Copeland et al., 2016; Dickinson-Copeland et al., 2015). Our findings align with previous studies showing increased Ang 2 and CXCL10 levels in serum (Wilson et al., 2011) and saliva of malarial patients (Lekpor et al., 2022), suggesting their potential as predictive markers for hemolysis-associated diseases (Wilson et al., 2013). Elevated CXCL10 has also been shown to play a significant role in SCD pathogenesis (Driss et al., 2012; Oxendine Harp et al., 2023) and our results support its utility in predicting disease outcomes. In our study, CXCL10 was differentially expressed across Hb genotypes, effectively distinguishing between HbAS, HbAC, HbCC, and healthy controls (HbAA) (Figures 1, 7). Circulating Ang 2 and CXCL10 levels positively correlated with hematological parameters such as Hb, WBC, and RBC counts (Figure 8; Supplementary Table S6–S8). Additionally, CCL11 levels were significantly higher in SCD than in healthy controls (Figure 7). Given its known role in neuroinflammatory and neurodegenerative disorders (Nazarinia et al., 2022) and cancer, (Zajkowska and Mroczko, 2021), elevated CCL11 in SCD could indicate neurologic complications during hemolysis-related crises.

BDNF, a nerve growth factor linked to neuronal survival, is associated with increased stroke risk in SCD patients, especially with higher transcranial Doppler (TCD) velocities (Chambliss et al., 2021; Hyacinth et al., 2012). Our study found significantly higher BDNF levels in genotype HbCC (Figure 1A), a variant unlike sickle-cell mutation, which provides some protection against malaria (Weatherall, 2008) but is associated with mild hemolytic anemia and joint pain (Karna et al., 2023). Altered BDNF levels have also been linked to pain severity in SCD (Sikandar et al., 2018). Consistent with previous studies (Chambliss et al., 2021; Lance et al., 2020), we observed elevated BDNF levels in children with SCD compared to healthy controls. BDNF is further implicated in ischemic and traumatic brain injuries, contributing to cerebrovascular complications in SCD (Chen et al., 2013; Korley et al., 2016; Drexelius et al., 2019). Our study identified a correlation between BDNF levels and sickle Hb genotypes (Figures 1A, 5, 7). Children with HbSS or HbSC under physiological stress may have high BDNF levels, suggesting a potential risk of stroke or pain crises.

The human immune response to diseases involves shifts in proinflammatory and anti-inflammatory cytokines levels, which play key roles in disease pathophysiology (Clark et al., 2008). In SCD, chronic inflammation and vasculopathy drive cytokine production, leukocyte release, and vascular endothelial activation, increasing adhesion molecules like ICAM 1 and VCAM 1. These changes enhance interactions between sickle erythrocytes and leukocytes, contributing to microvascular occlusion and tissue damage (Jacobs et al., 2013; Telen et al., 2019; Thomas et al., 2022). Our findings showed elevated proinflammatory cytokines and chemokines (TNF-α, IL-6, IFN-γ, IL-10, CCL11, MDC, MIP-1β, MCP-1, IL-8, IL-16, IL-12p40), vascular injury markers (Ang 1, Ang 2, ICAM 1 and VCAM 1), angiogenic factors (VEGF A, VEGF-D, PlGF, bFGF, Flt-1), free heme, HO-1 and reduced levels of heme scavenger proteins (Hemopexin, Haptoglobin) in HbSS and HbSC patients compared to HbAA controls and other sickle Hb genotypes (Figures 1, 7). Elevated IL-6 and TNF-α reflect chronic inflammation, with other cytokines enhancing RBC adhesion and contributing to vaso-occlusion (Pathare et al., 2004; Bashi et al., 2023). TNF-α, produced by macrophages and T cells, plays a central role in acute inflammation and pain crises (Conran and Belcher, 2018). Our findings showed slightly higher TNF-α levels in HbSS compared to HbSC and effectively distinguished between HbAA, HbAS, HbAC, and HbCC (Figure 1). TNF-α also activates endothelial cells, increasing vascular permeability and adhesion molecules expression (Sedger and McDermott, 2014), which exacerbates pain. Elevated TNF-α levels have been implicated in severe malaria and pediatric SCD, highlighting their significance in hemoglobinopathies (Leão et al., 2020; Perera et al., 2013; Darbari et al., 2020). In agreement with Pathare et al. (2004), elevated TNF-α and IL-6 levels observed in our study are likely linked to increased pain crises seen in SCD (Damanhouri et al., 2015). Higher TNF-α and IL-6 levels in HbSS compared to HbAA are linked to increased vaso-occlusive crises and stroke risk (Sarray et al., 2015; Darbari et al., 2020). Additionally, IL-6 also distinguished HbSS from HbAC and HbAS from HbSC, which may play a role in acute phase protein synthesis (Keikhaei et al., 2013; Qari et al., 2012). IL-6 may also suppress TNF-α and enhance IL-10 production, offering therapeutic potential insights (Xing et al., 1998). In this study, levels of Ang 1 were slightly increased, and Tie 2 was reduced in SCD compared to other Hb genotypes (Figures 1, 7), suggesting impaired endothelial function and vascular instability under hypoxic conditions common in SCD. Ang 1 stabilizes the endothelium by binding it to Tie 2, while Ang 2, Tie-2, PIGF, and VEGF play key roles in endothelial activation. Ang 2, a critical mediator of angiogenesis and inflammation, antagonizes Tie 2, promoting vascular instability (Scholz et al., 2015; Fiedler et al., 2006). The reduction in Tie 2 limits the endothelium’s ability to respond to Ang 1 despite its slight increase, leading to increased vascular permeability and heightened inflammation and organ damage (Akwii et al., 2019). This imbalance may reflect ongoing inflammation or vascular stress, such as during VOC episodes. Elevated Ang 2 levels, which have been associated with endothelial injury in severe malaria (Yeo et al., 2008; Conroy et al., 2012), also correlate with severe infections and TNF-α secretion, highlighting its potential as a therapeutic target for managing disease complications. The distinct expression patterns of BDNF, CXCL10, Ang-2, TNF-α and IL-6 across Hb genotypes, especially in SCD, highlight their potential as predictive markers for disease crises in HbSS patients. Our study also found elevated IL-12p40 and IL-16 levels in SCD compared to controls (Figure 7). IL-12 promotes Th1 induction and INF-γ expression, supporting T-cell activation and pro-inflammatory cytokine production (Pathare et al., 2004; Keikhaei et al., 2013). Chronic inflammation and cell-free Hb drive IL-12p40 output, while IL-16 acts as a chemoattractant (Vignali and Kuchroo, 2012), regulating immune responses and contributing to leukocyte recruitment and vaso-occlusive crises in SCD (Wu et al., 2023; Kaser et al., 2000; Morikis et al., 2021).

Our study found significant differences in ICAM-1 and VCAM-1 levels between HbSS and HbAA (Figure 7). These molecules promote sickle RBC and leukocyte adhesion, contributing to VOC, tissue ischemia and SCD pathology, making them potential markers and therapeutic targets (Belcher et al., 2010; White et al., 2020). SCD-associated inflammation and hypoxia elevate angiogenic mediators like VEGF-A, VEGF-D, PlGF, and Ang-2, driving abnormal angiogenesis, vascular permeability and complications such as pulmonary hypertension (Conran and Belcher, 2018; Eddy et al., 2018; Brittain et al., 2010; Niu et al., 2009; Duits et al., 2006). Our data showed similar increases in these angiogenic mediators (Figure 7). We observed significant differences in PIGF, VEGF, and Flt-1 between SCD patients and controls, with PIGF distinguishing HbSS from HbAA (Figure 7). Free heme stimulates PlGF expression, correlating with VOC episodes (Perelman et al., 2003; Gu et al., 2018) while its elevated levels may reflect compensatory responses to hypoxia and anemia crises (Tordjman et al., 2001). These mediators, also implicated in cancer, cardiovascular diseases (Luttun et al., 2002; Fischer et al., 2007), and pregnancy-related disorders (Eddy et al., 2018), are promising therapeutic targets in SCD. Understanding their roles offers new insights into managing SCD and related complications through targeted therapies.

We evaluated the expression of heme and hemolysis-related cytoprotective proteins across various sickle Hb genotypes. In SCD, increased hemolysis elevates free heme levels, managed by scavenger proteins like Hpx, Hp, and HO-1, which often decrease during severe hemolysis (Belcher et al., 2018). HO-1, Hpx, and Hp levels varied significantly across Hb genotypes and correlated with hematological parameters (WBC, RBC, Hb) (Figure 2; Supplementary Table S7–S9). Our findings showed elevated heme and HO-1 in HbSS and HbSC, with corresponding decreases in Hp and Hpx, indicating an overwhelmed scavenging system in SCA (Muller-Eberhard et al., 1968) (Figure 2). Free heme, a potent inducer of vascular inflammation and ROS is mitigated by HO-1, which generates anti-inflammatory molecules like biliverdin and carbon monoxide (Gozzelino et al., 2010). Mouse models highlight HO-1’s role in preventing vascular inflammation and reducing vaso-occlusion (Belcher et al., 2006; Datta et al., 2007). Its anti-inflammatory and anti-apoptotic functions counteract heme’s harmful effects (Conran and Belcher, 2018; Belcher et al., 2006; Datta et al., 2010; Bean et al., 2012). Free heme drives SCA pathophysiology by promoting HbS polymerization and cytotoxicity (Rees et al., 2010), while lower cell-free heme levels in HbAS and HbAA suggest a more effective scavenging system.

Our study also revealed that concentrations of several inflammatory markers correlated with hematological parameters (Hb, WBC, and RBC) across different sickle Hb genotypes, as did heme and scavenger protein levels with complete blood counts (Figure 8; Supplementary Figure S5). Our analysis identified several significant correlations (*p* < 0.05), though with low *R* ^2^ values, indicating these relationships explain only a small portion of the variance. While these findings offer initial insights, they underscore the need for larger studies to clarify these associations and their clinical relevance.

ROC analysis and AUC evaluated proinflammatory, angiogenic, and injury markers as potential predictors in SCA. Differential expressions of CXCL10, BDNF, Ang 2, TNF-α, IL-6, CCL11, ICAM-1, VCAM-1, VEGF, Flt-1, Tie-2, and PIGF was observed (Figures 5, 6) with notably high AUC values identified for CXCL10, BDNF, Ang 2, CCL11, ICAM-1, VCAM-1, Flt-1, VEGF-D, and PIGF (Figure 5; Supplementary Figure S4). While individual markers may not reliably predict SCD complications, a combination of multiple biomarkers or clinical factors could provide a more accurate assessment (Chambliss et al., 2023).

High AUC values were found for ratios such as CXCL10:BDNF, Ang 2:BDNF, BDNF:Ang 1, and Ang 2:Ang 1 across different Hb genotypes (Figure 4; Supplementary Figure S2, 3). For example, the Ang 2:Ang 1 ratio’s high AUC suggests endothelial dysfunction and more severe disease. These markers could support disease monitoring and inform treatment adjustments. Also, ratios such as Heme:BDNF, CXCL10:HO-1, CXCL10:Hp, Ang 2:HO-1, Ang 2:Hpx, Ang 2:Hp and BDNF:HO-1, BDNF:Hpx and BDNF:Hp with high AUC values suggest potential disease complications risk (Figure 3; Supplementary Figure S2, 3). Hemopexin and haptoglobin, key heme scavengers, neutralize and transport free heme for degradation. A balanced heme-to-scavenger ratio reflects effective heme clearance, reducing toxicity, while a low ratio indicates inadequate scavenging, increasing oxidative stress and inflammation, and disease severity. These findings provide insights into the interplay between pro-oxidants and antioxidants in hemolysis-driven conditions like SCD.

ROC and AUC also assess individual biomarker ratios as predictors of SCD complications. Key markers including CXCL10, BDNF, Ang 2, IL-1, TNF-α, CCL11, ICAM-1, VCAM-1, Flt-1, PIGF, and VEGFA were evaluated. Ratios such as IL-6:IL-10, TNF-α:IL-10, IL-12A:IL-10, Ang 2:Tie 2, Ang 2:Ang 1, ICAM 1:VCAM 1,VEGFA:Ang 2, BDNF:CCL11, and VEGFA:PIGF showed strong predictive power, with ratios of ICAM 1: VCAM 1, VEGFA:Ang 2, BDNF:CCL11,and VEGFA:PIGF achieve a perfect AUC of 1.00 (Figure 6). Ratios with AUC values of ≥0.75 are clinically relevant (White et al., 2023), though their utility may vary by context. For instance, the ratio IL-6:IL-10 (AUC = 0.93) indicates heightened inflammation linked to severe vaso-occlusive crises. Elevated TNF-α relative to IL-10 suggests signals of acute inflammation and predicts pain crises, while IL-12A:IL-10 ratio (AUC = 0.93) reflects immune status and inflammatory risk. These ratios help stratify patients, guide preventive care, and monitor disease activity. Higher ratios indicate increased risk, prompting proactive management. By capturing the balance between pro- and anti-inflammatory cytokines, they offer insights into treatment response and disease progression, enabling personalized care tailored to each patient’s cytokine profile for more effective SCD management.

Also, high AUC for Ang 2:Ang1, Ang 2:Tie 2, ICAM1:VCAM-1, and VEGFA: Ang 2 ratios suggest endothelial dysfunction and instability, key factors in SCD and angiogenesis. Ang 2 regulates blood vessel formation, while Tie 2 drives angiogenic signaling. Ratios with AUC of 0.98, 0.93, 0.98 and 1.00, respectively, reflect endothelial dysfunction linked to vaso-occlusive crises. Additionally, The BDNF: CCL11(AUC = 1.00) reflects the balance between neuroprotection and neuroinflammation, relevant for managing pain, cognitive issues and stroke risk in SCD, consistent with previous findings (Chambliss et al., 2021).

Furthermore, VEGFA: Ang 2 and VEGFA: PIGF with an AUC value of 1.00, respectively, highlight significant angiogenic activity. VEGFA promotes vascular growth, while Ang 2 destabilizes microvessels. A high AUC for VEGFA:Ang 2 and Ang 2:VEGFA ratios suggests a potential predictor for angiogenic imbalances in SCD, possibly linked to complications like priapism or leg ulcers. Similarly, the balance between VEGFA and PIGF, as indicated by their AUCs, may correlate with vascular outcomes and complications. Utilizing these ratios is crucial for highlighting relative differences in cytokine responses among children with SCD, SCT, and healthy controls. These ratios clarify the biological significance of changes in heme scavengers and cytokine profiles, shedding light on unique inflammatory processes in each group. By focusing on these relative measurements, we can identify potential biomarkers, such as specific cytokine ratios, that are key for distinguishing disease states and understanding the pathophysiology of SCD, ultimately supporting the development of targeted therapeutic strategies.

The Principal Component Analysis (PCA) of CBC, cytokine, and chemokine levels showed that HbSS and HbSC Hb genotypes form distinct clusters from other Hb groups, especially when external factors unrelated to SCD are excluded (PCA; Figure 9). This suggests that HbSS and HbSC Hb genotypes exhibit unique blood characteristics and immune response markers, highlighting the potential of using CBC, cytokine, and chemokine profiles to differentiate SCD forms and predict potential crises. This differentiation is vital for accurate diagnosis, prognosis, and the development of personalized treatment strategies.

Our study highlights the importance of modulating biomarker levels to reduce inflammation and SCD complications. It also emphasizes the need for a reliable test to measure free heme and related inflammatory mediators, which is crucial for understanding heme toxicity. Current tests only indirectly assess hemolysis severity by measuring Hp and Hpx levels (Belcher et al., 2018), leaving the full extent of heme toxicity and its broader effects on the body unclear (Immenschuh et al., 2017). We conducted a cross-sectional study comparing sickle Hb genotypes to identify biomarkers for therapeutic interventions and predict hemolysis-associated crises in SCD. This study, the first to examine such an extensive combination of circulating factors in children, offers insights into biomarker signatures that distinguish between mild and severe disease, enhancing our understanding of SCD pathophysiology and guiding clinical management. Predicting SCD outcomes is challenging due to its variability. Our study identified several biomarkers linked to risk of complications, highlighting the need to model multiple biomarkers, especially in children, to improve disease management.

Overexpression of inflammatory, injury, and angiogenic markers contributes to chronic inflammation and heme toxicity in SCA patients. Assessing biomarkers like BDNF, Ang 2, CXCL10, CCL11, TNF-α, IL-6, ICAM1, VCAM1, Tie 2, and VEGFA could inform therapeutic strategies and Table 2 summarizes the detailed roles and functions of these biomarkers associated with SCD. Biomarker evaluation is already a routine practice in clinical settings, and obtaining data for managing pediatric SCD patient can be done quickly. Blood samples are easily collected during routine visits or hospital admissions, with analysis completed via standard tests like multiplex assays or ELISA—often within 24 h or even faster in on-site labs. Rapid interpretation of results allows clinicians to promptly assess and initiate interventions. As point-of-care testing advances, biomarker tests at the bedside will further reduce wait times, enabling immediate clinical decisions and seamless integration into routine care.

The conclusion of this study is limited by the small sample size, although there was a strong association between some circulating markers levels and clinical characteristics. While this study assessed the sensitivity and specificity of these markers, further research using simple random sampling, larger pediatric sample sizes is needed. Additionally, longitudinal assessment of within-genotype differences is required to correlate these findings with disease severity and validate their universality and reliability across diverse populations, supporting more robust clinical applications. These findings emphasize the role of inflammation, angiogenesis, and endothelial activation in SCD pathophysiology, highlighting potential biomarkers for monitoring and management through specific biomarkers that predict disease crises and guide personalized therapy in SCD.

## Data Availability

The original contributions presented in the study are included in the article/Supplementary Material, further inquiries can be directed to the corresponding authors.
