# Supplementary material for: Circulating biomarkers associated with pediatric sickle cell disease
Source: Front Mol Biosci. 2024 Dec 19;11:1481441. doi: 10.3389/fmolb.2024.1481441 (PMC11694143; doi:10.3389/fmolb.2024.1481441)
Supplement: Supplementary file 1 [file Presentation1.pptx]

## Slide 1
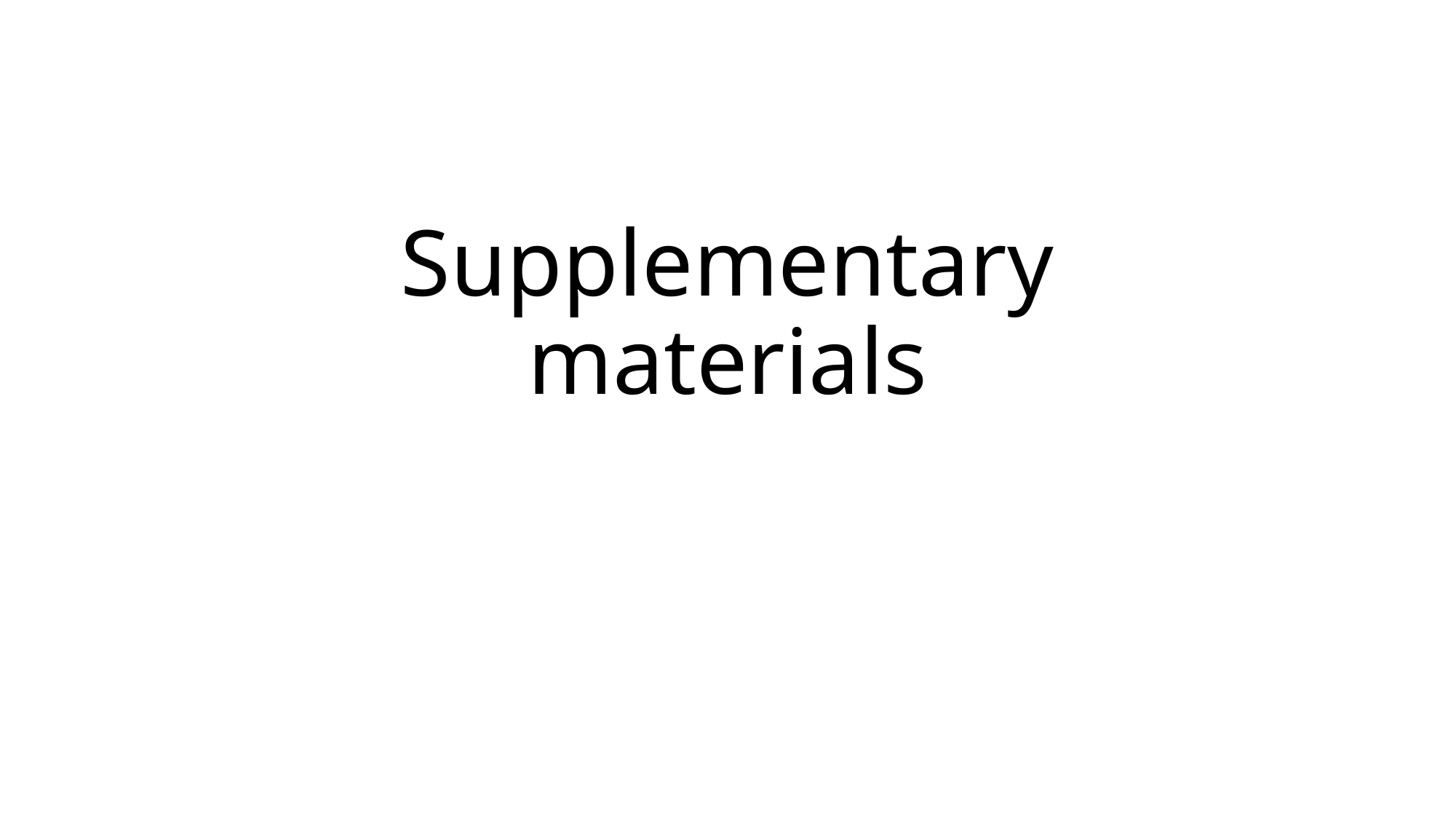

# Supplementary materials

## Slide 2
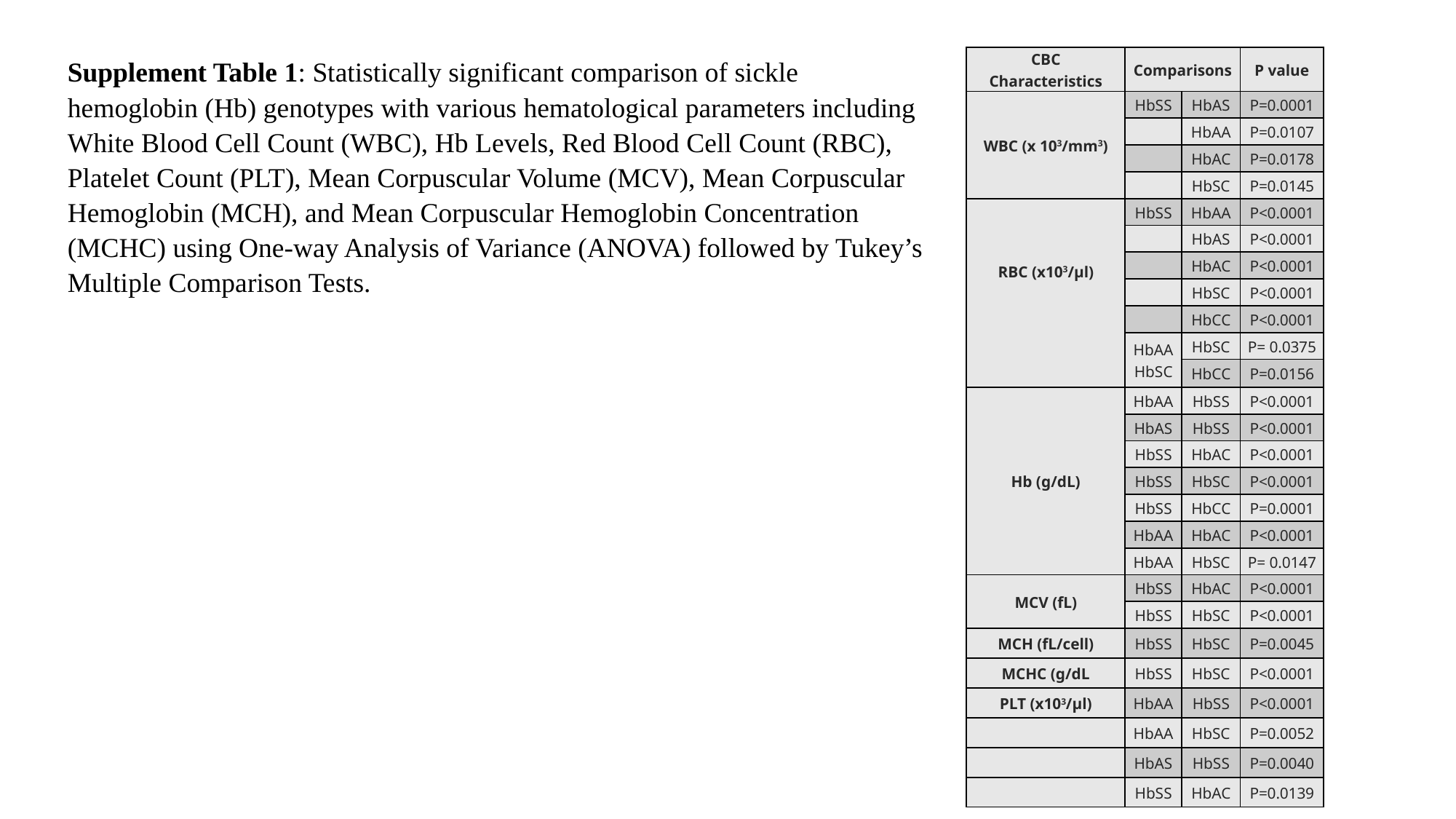

Supplement Table 1: Statistically significant comparison of sickle hemoglobin (Hb) genotypes with various hematological parameters including White Blood Cell Count (WBC), Hb Levels, Red Blood Cell Count (RBC), Platelet Count (PLT), Mean Corpuscular Volume (MCV), Mean Corpuscular Hemoglobin (MCH), and Mean Corpuscular Hemoglobin Concentration (MCHC) using One-way Analysis of Variance (ANOVA) followed by Tukey’s Multiple Comparison Tests.
| CBC Characteristics | Comparisons | | P value |
| --- | --- | --- | --- |
| WBC (x 103/mm3) | HbSS | HbAS | P=0.0001 |
| | | HbAA | P=0.0107 |
| | | HbAC | P=0.0178 |
| | | HbSC | P=0.0145 |
| RBC (x103/µl) | HbSS | HbAA | P<0.0001 |
| | | HbAS | P<0.0001 |
| | | HbAC | P<0.0001 |
| | | HbSC | P<0.0001 |
| | | HbCC | P<0.0001 |
| | HbAA HbSC | HbSC | P= 0.0375 |
| | | HbCC | P=0.0156 |
| Hb (g/dL) | HbAA | HbSS | P<0.0001 |
| | HbAS | HbSS | P<0.0001 |
| | HbSS | HbAC | P<0.0001 |
| | HbSS | HbSC | P<0.0001 |
| | HbSS | HbCC | P=0.0001 |
| | HbAA | HbAC | P<0.0001 |
| | HbAA | HbSC | P= 0.0147 |
| MCV (fL) | HbSS | HbAC | P<0.0001 |
| | HbSS | HbSC | P<0.0001 |
| MCH (fL/cell) | HbSS | HbSC | P=0.0045 |
| MCHC (g/dL | HbSS | HbSC | P<0.0001 |
| PLT (x103/µl) | HbAA | HbSS | P<0.0001 |
| | HbAA | HbSC | P=0.0052 |
| | HbAS | HbSS | P=0.0040 |
| | HbSS | HbAC | P=0.0139 |

## Slide 3
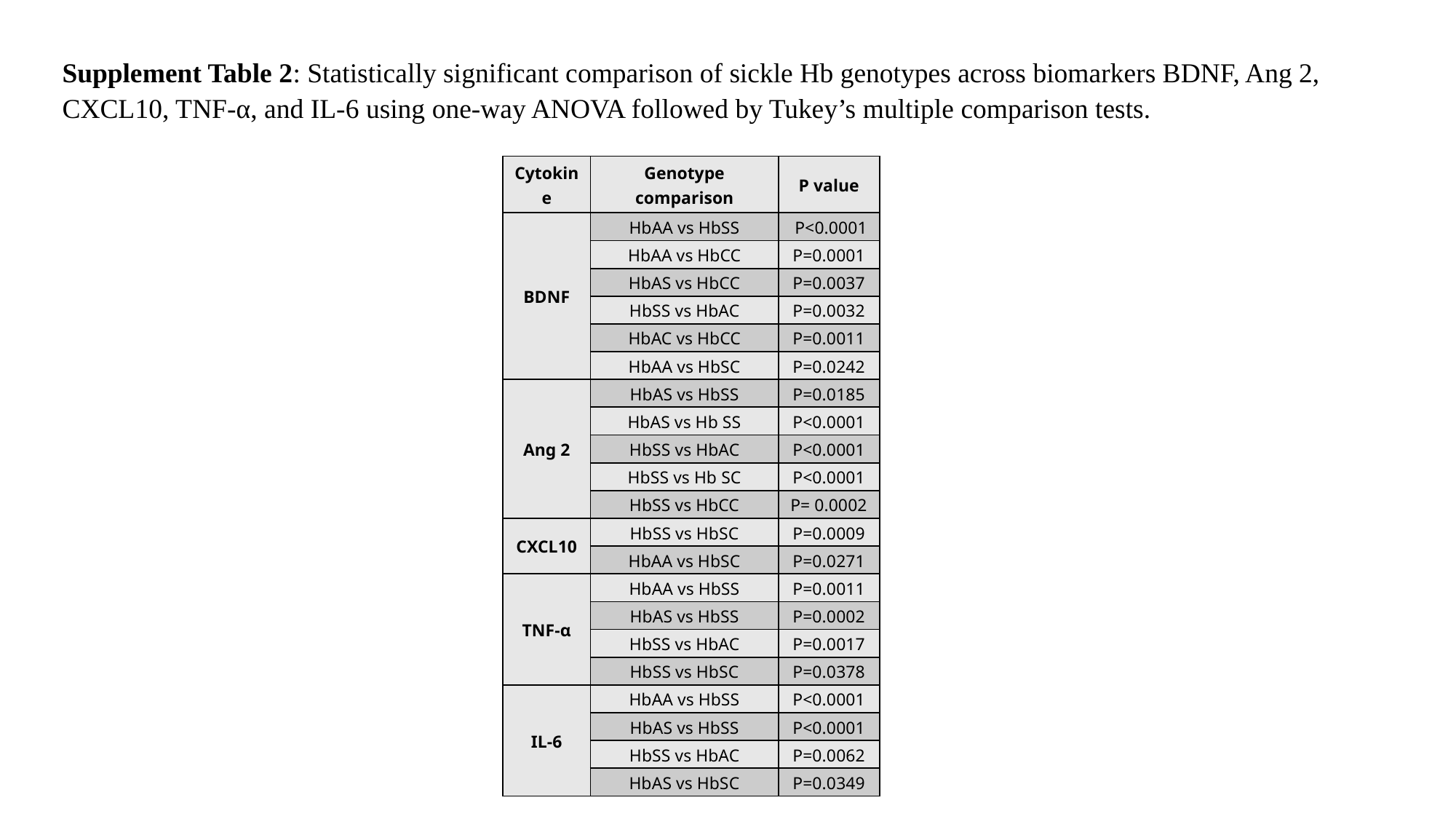

Supplement Table 2: Statistically significant comparison of sickle Hb genotypes across biomarkers BDNF, Ang 2, CXCL10, TNF-α, and IL-6 using one-way ANOVA followed by Tukey’s multiple comparison tests.
| Cytokine | Genotype comparison | P value |
| --- | --- | --- |
| BDNF | HbAA vs HbSS | P<0.0001 |
| | HbAA vs HbCC | P=0.0001 |
| | HbAS vs HbCC | P=0.0037 |
| | HbSS vs HbAC | P=0.0032 |
| | HbAC vs HbCC | P=0.0011 |
| | HbAA vs HbSC | P=0.0242 |
| Ang 2 | HbAS vs HbSS | P=0.0185 |
| | HbAS vs Hb SS | P<0.0001 |
| | HbSS vs HbAC | P<0.0001 |
| | HbSS vs Hb SC | P<0.0001 |
| | HbSS vs HbCC | P= 0.0002 |
| CXCL10 | HbSS vs HbSC | P=0.0009 |
| | HbAA vs HbSC | P=0.0271 |
| TNF-α | HbAA vs HbSS | P=0.0011 |
| | HbAS vs HbSS | P=0.0002 |
| | HbSS vs HbAC | P=0.0017 |
| | HbSS vs HbSC | P=0.0378 |
| IL-6 | HbAA vs HbSS | P<0.0001 |
| | HbAS vs HbSS | P<0.0001 |
| | HbSS vs HbAC | P=0.0062 |
| | HbAS vs HbSC | P=0.0349 |

## Slide 4
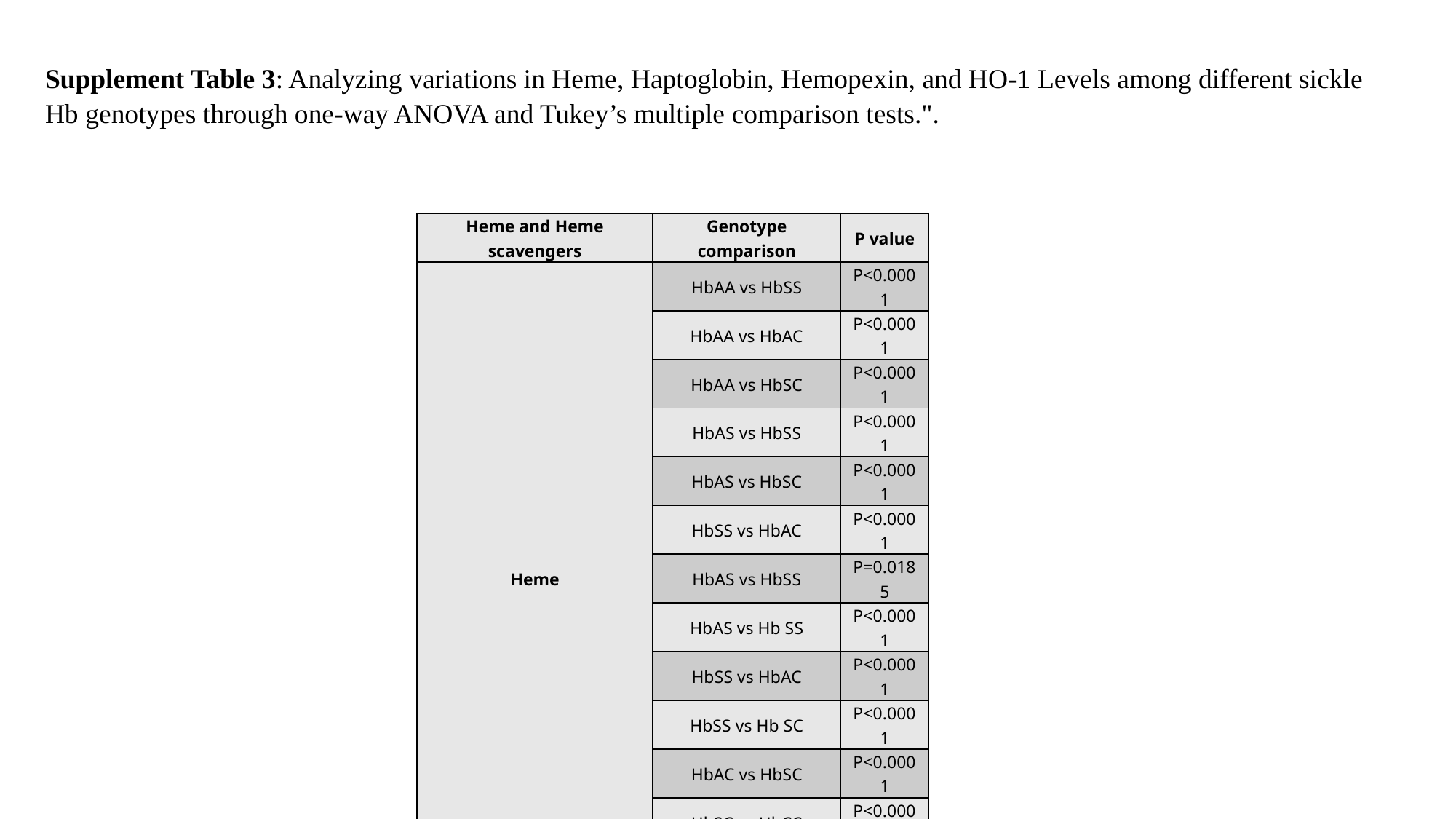

Supplement Table 3: Analyzing variations in Heme, Haptoglobin, Hemopexin, and HO-1 Levels among different sickle Hb genotypes through one-way ANOVA and Tukey’s multiple comparison tests.".
| Heme and Heme scavengers | Genotype comparison | P value |
| --- | --- | --- |
| Heme | HbAA vs HbSS | P<0.0001 |
| | HbAA vs HbAC | P<0.0001 |
| | HbAA vs HbSC | P<0.0001 |
| | HbAS vs HbSS | P<0.0001 |
| | HbAS vs HbSC | P<0.0001 |
| | HbSS vs HbAC | P<0.0001 |
| | HbAS vs HbSS | P=0.0185 |
| | HbAS vs Hb SS | P<0.0001 |
| | HbSS vs HbAC | P<0.0001 |
| | HbSS vs Hb SC | P<0.0001 |
| | HbAC vs HbSC | P<0.0001 |
| | HbSC vs HbCC | P<0.0001 |
| | HbSS vs HbCC | P=0.0002 |
| Haptoglobin | HbAA vs HBSS | P<0.0001 |
| | HbAA vs HbSC | P<0.0001 |
| | HbAS vs HbSC | P<0.0001 |
| | HbAC vs HbSC | P<0.0001 |
| | HbSS vs HbAC | P=0.0007 |
| Hemopexin | HbAA vs HbSC | P=0.0416 |
| HO-1 | HbAA vs HbSS | P<0.0001 |
| | HbAS vs HbSS | P<0.0001 |
| | HbSS vs HbAC | P<0.0001 |
| | HbSS vs HbSC | P<0.0001 |
| | HbSS vs HbCC | P=0.0005 |

## Slide 5
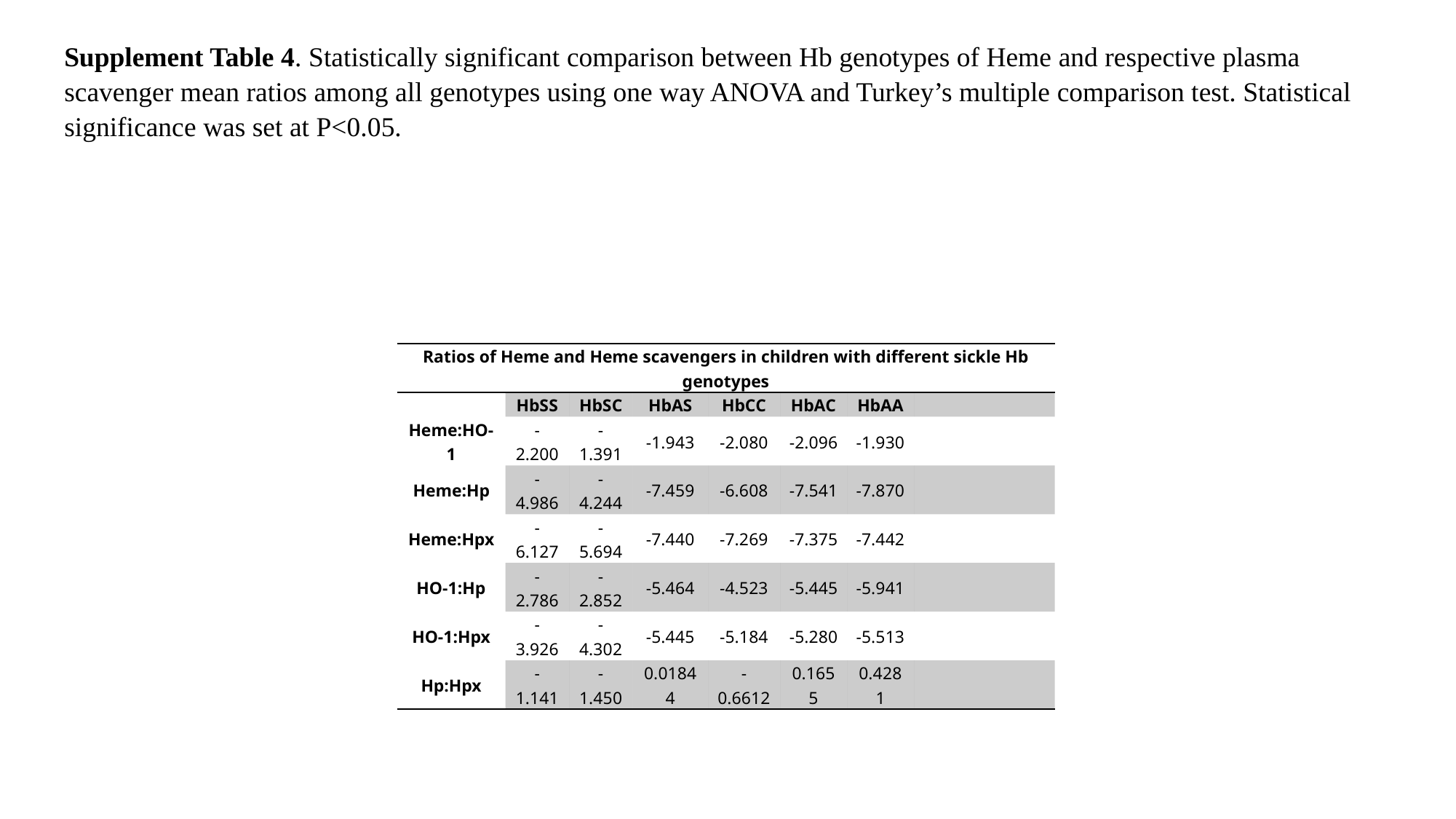

Supplement Table 4. Statistically significant comparison between Hb genotypes of Heme and respective plasma scavenger mean ratios among all genotypes using one way ANOVA and Turkey’s multiple comparison test. Statistical significance was set at P<0.05.
| Ratios of Heme and Heme scavengers in children with different sickle Hb genotypes | | | | | | | |
| --- | --- | --- | --- | --- | --- | --- | --- |
| | HbSS | HbSC | HbAS | HbCC | HbAC | HbAA | |
| Heme:HO-1 | -2.200 | -1.391 | -1.943 | -2.080 | -2.096 | -1.930 | |
| Heme:Hp | -4.986 | -4.244 | -7.459 | -6.608 | -7.541 | -7.870 | |
| Heme:Hpx | -6.127 | -5.694 | -7.440 | -7.269 | -7.375 | -7.442 | |
| HO-1:Hp | -2.786 | -2.852 | -5.464 | -4.523 | -5.445 | -5.941 | |
| HO-1:Hpx | -3.926 | -4.302 | -5.445 | -5.184 | -5.280 | -5.513 | |
| Hp:Hpx | -1.141 | -1.450 | 0.01844 | -0.6612 | 0.1655 | 0.4281 | |

## Slide 6
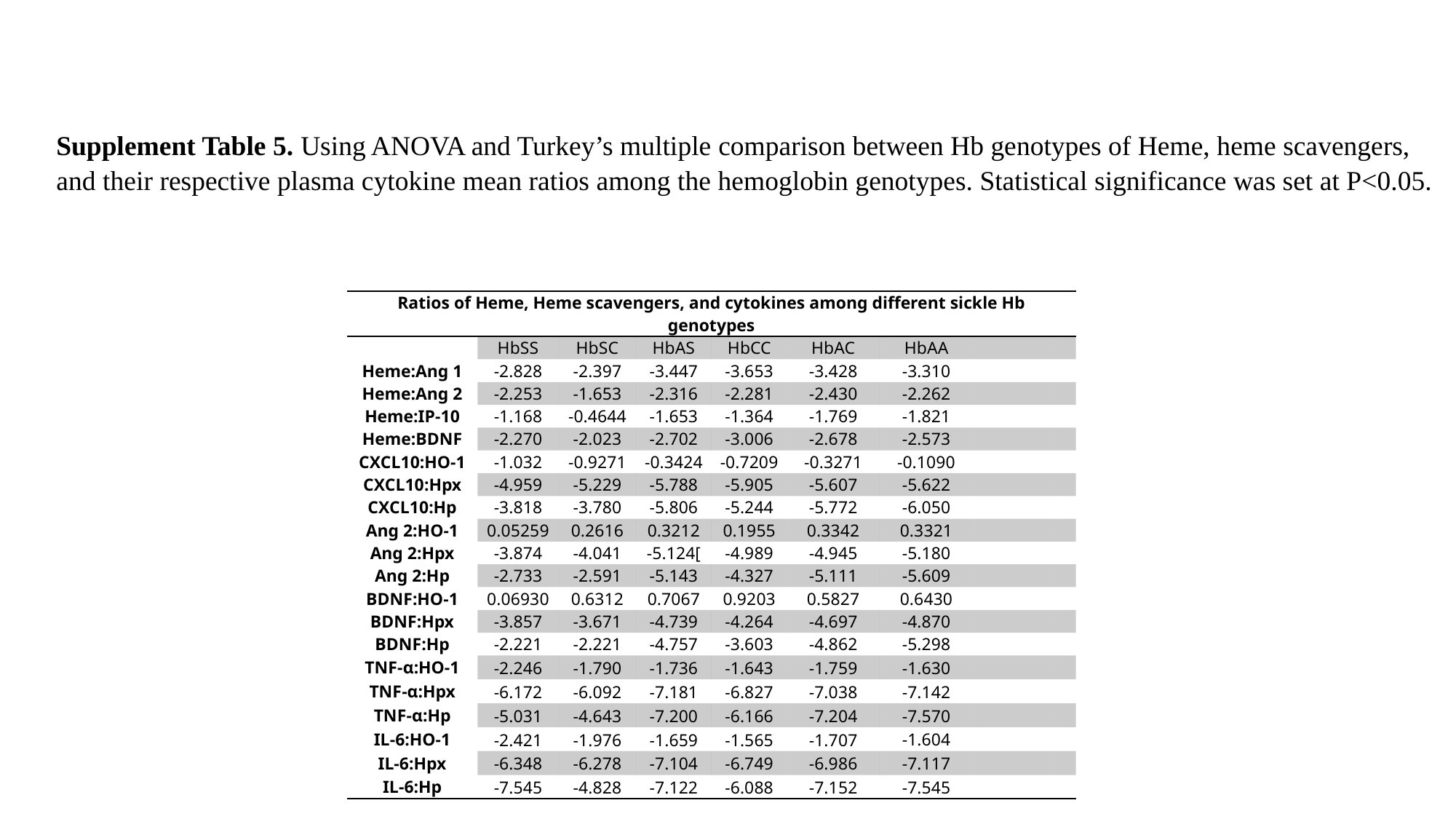

Supplement Table 5. Using ANOVA and Turkey’s multiple comparison between Hb genotypes of Heme, heme scavengers, and their respective plasma cytokine mean ratios among the hemoglobin genotypes. Statistical significance was set at P<0.05.
| Ratios of Heme, Heme scavengers, and cytokines among different sickle Hb genotypes | | | | | | | | |
| --- | --- | --- | --- | --- | --- | --- | --- | --- |
| | HbSS | HbSC | HbAS | HbCC | HbAC | HbAA | | |
| Heme:Ang 1 | -2.828 | -2.397 | -3.447 | -3.653 | -3.428 | -3.310 | | |
| Heme:Ang 2 | -2.253 | -1.653 | -2.316 | -2.281 | -2.430 | -2.262 | | |
| Heme:IP-10 | -1.168 | -0.4644 | -1.653 | -1.364 | -1.769 | -1.821 | | |
| Heme:BDNF | -2.270 | -2.023 | -2.702 | -3.006 | -2.678 | -2.573 | | |
| CXCL10:HO-1 | -1.032 | -0.9271 | -0.3424 | -0.7209 | -0.3271 | -0.1090 | | |
| CXCL10:Hpx | -4.959 | -5.229 | -5.788 | -5.905 | -5.607 | -5.622 | | |
| CXCL10:Hp | -3.818 | -3.780 | -5.806 | -5.244 | -5.772 | -6.050 | | |
| Ang 2:HO-1 | 0.05259 | 0.2616 | 0.3212 | 0.1955 | 0.3342 | 0.3321 | | |
| Ang 2:Hpx | -3.874 | -4.041 | -5.124[ | -4.989 | -4.945 | -5.180 | | |
| Ang 2:Hp | -2.733 | -2.591 | -5.143 | -4.327 | -5.111 | -5.609 | | |
| BDNF:HO-1 | 0.06930 | 0.6312 | 0.7067 | 0.9203 | 0.5827 | 0.6430 | | |
| BDNF:Hpx | -3.857 | -3.671 | -4.739 | -4.264 | -4.697 | -4.870 | | |
| BDNF:Hp | -2.221 | -2.221 | -4.757 | -3.603 | -4.862 | -5.298 | | |
| TNF-α:HO-1 | -2.246 | -1.790 | -1.736 | -1.643 | -1.759 | -1.630 | | |
| TNF-α:Hpx | -6.172 | -6.092 | -7.181 | -6.827 | -7.038 | -7.142 | | |
| TNF-α:Hp | -5.031 | -4.643 | -7.200 | -6.166 | -7.204 | -7.570 | | |
| IL-6:HO-1 | -2.421 | -1.976 | -1.659 | -1.565 | -1.707 | -1.604 | | |
| IL-6:Hpx | -6.348 | -6.278 | -7.104 | -6.749 | -6.986 | -7.117 | | |
| IL-6:Hp | -7.545 | -4.828 | -7.122 | -6.088 | -7.152 | -7.545 | | |

## Slide 7
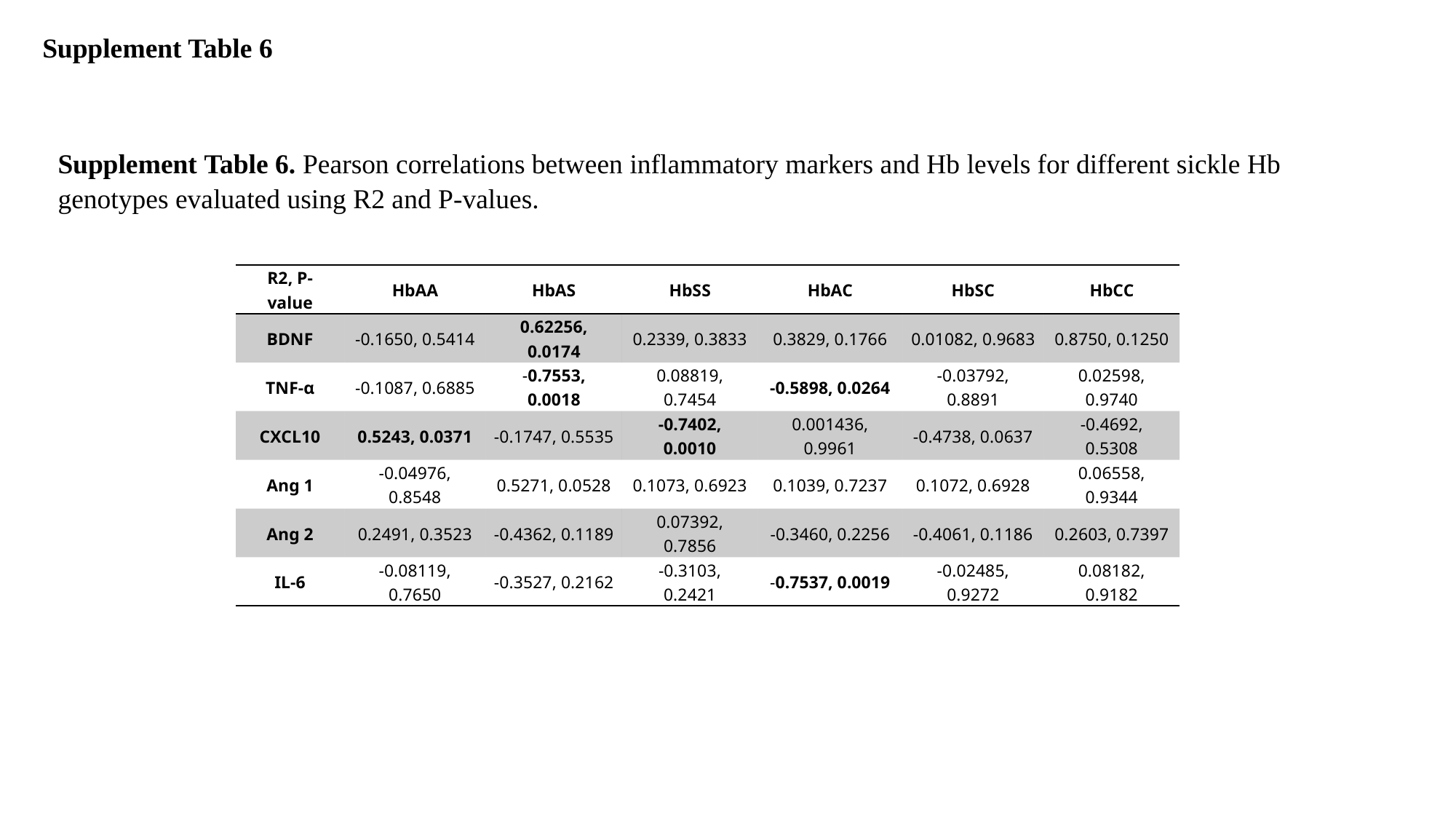

Supplement Table 6
Supplement Table 6. Pearson correlations between inflammatory markers and Hb levels for different sickle Hb genotypes evaluated using R2 and P-values.
| R2, P- value | HbAA | HbAS | HbSS | HbAC | HbSC | HbCC |
| --- | --- | --- | --- | --- | --- | --- |
| BDNF | -0.1650, 0.5414 | 0.62256, 0.0174 | 0.2339, 0.3833 | 0.3829, 0.1766 | 0.01082, 0.9683 | 0.8750, 0.1250 |
| TNF-α | -0.1087, 0.6885 | -0.7553, 0.0018 | 0.08819, 0.7454 | -0.5898, 0.0264 | -0.03792, 0.8891 | 0.02598, 0.9740 |
| CXCL10 | 0.5243, 0.0371 | -0.1747, 0.5535 | -0.7402, 0.0010 | 0.001436, 0.9961 | -0.4738, 0.0637 | -0.4692, 0.5308 |
| Ang 1 | -0.04976, 0.8548 | 0.5271, 0.0528 | 0.1073, 0.6923 | 0.1039, 0.7237 | 0.1072, 0.6928 | 0.06558, 0.9344 |
| Ang 2 | 0.2491, 0.3523 | -0.4362, 0.1189 | 0.07392, 0.7856 | -0.3460, 0.2256 | -0.4061, 0.1186 | 0.2603, 0.7397 |
| IL-6 | -0.08119, 0.7650 | -0.3527, 0.2162 | -0.3103, 0.2421 | -0.7537, 0.0019 | -0.02485, 0.9272 | 0.08182, 0.9182 |

## Slide 8
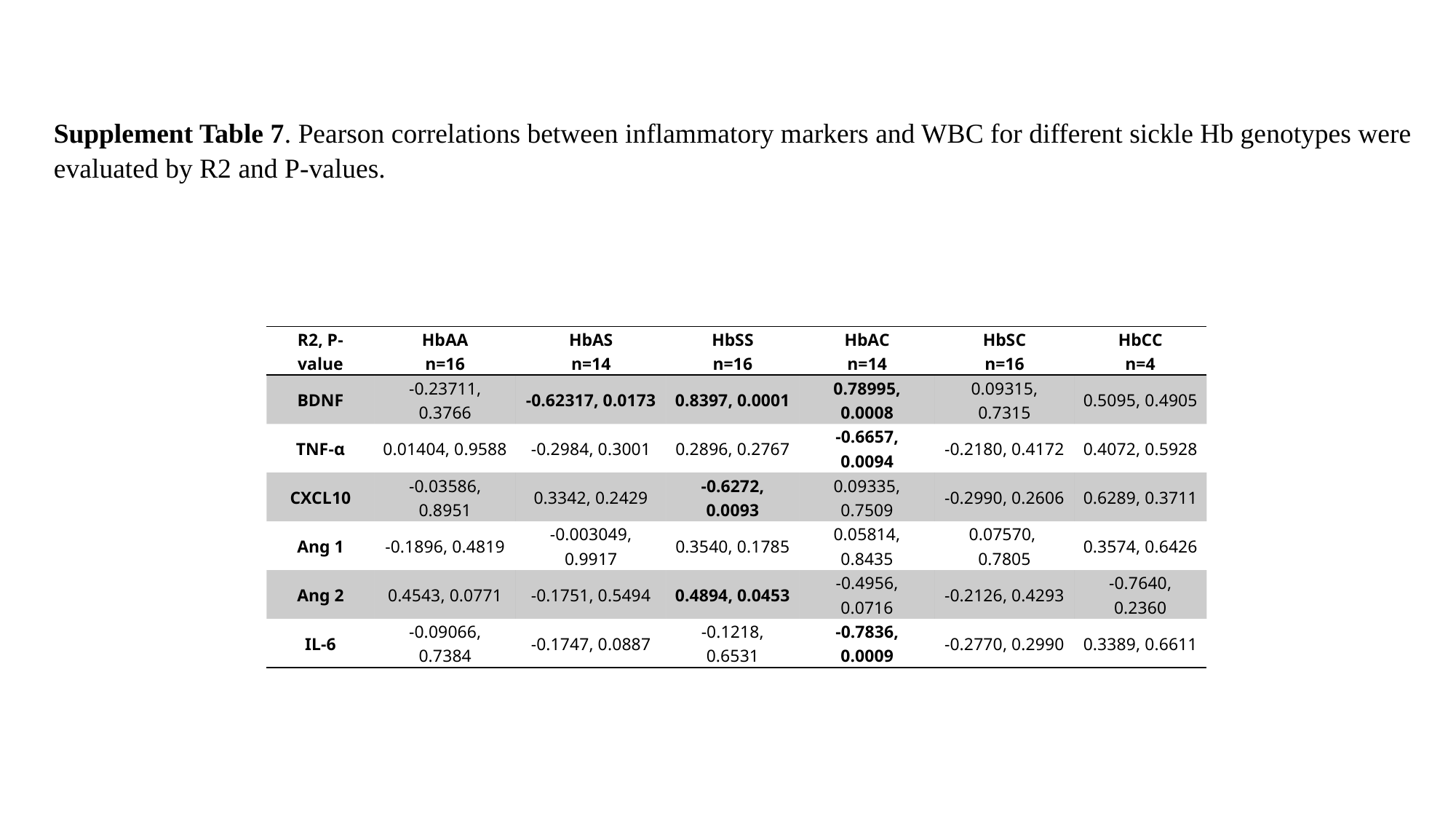

Supplement Table 7. Pearson correlations between inflammatory markers and WBC for different sickle Hb genotypes were evaluated by R2 and P-values.
| R2, P- value | HbAA n=16 | HbAS n=14 | HbSS n=16 | HbAC n=14 | HbSC n=16 | HbCC n=4 |
| --- | --- | --- | --- | --- | --- | --- |
| BDNF | -0.23711, 0.3766 | -0.62317, 0.0173 | 0.8397, 0.0001 | 0.78995, 0.0008 | 0.09315, 0.7315 | 0.5095, 0.4905 |
| TNF-α | 0.01404, 0.9588 | -0.2984, 0.3001 | 0.2896, 0.2767 | -0.6657, 0.0094 | -0.2180, 0.4172 | 0.4072, 0.5928 |
| CXCL10 | -0.03586, 0.8951 | 0.3342, 0.2429 | -0.6272, 0.0093 | 0.09335, 0.7509 | -0.2990, 0.2606 | 0.6289, 0.3711 |
| Ang 1 | -0.1896, 0.4819 | -0.003049, 0.9917 | 0.3540, 0.1785 | 0.05814, 0.8435 | 0.07570, 0.7805 | 0.3574, 0.6426 |
| Ang 2 | 0.4543, 0.0771 | -0.1751, 0.5494 | 0.4894, 0.0453 | -0.4956, 0.0716 | -0.2126, 0.4293 | -0.7640, 0.2360 |
| IL-6 | -0.09066, 0.7384 | -0.1747, 0.0887 | -0.1218, 0.6531 | -0.7836, 0.0009 | -0.2770, 0.2990 | 0.3389, 0.6611 |

## Slide 9
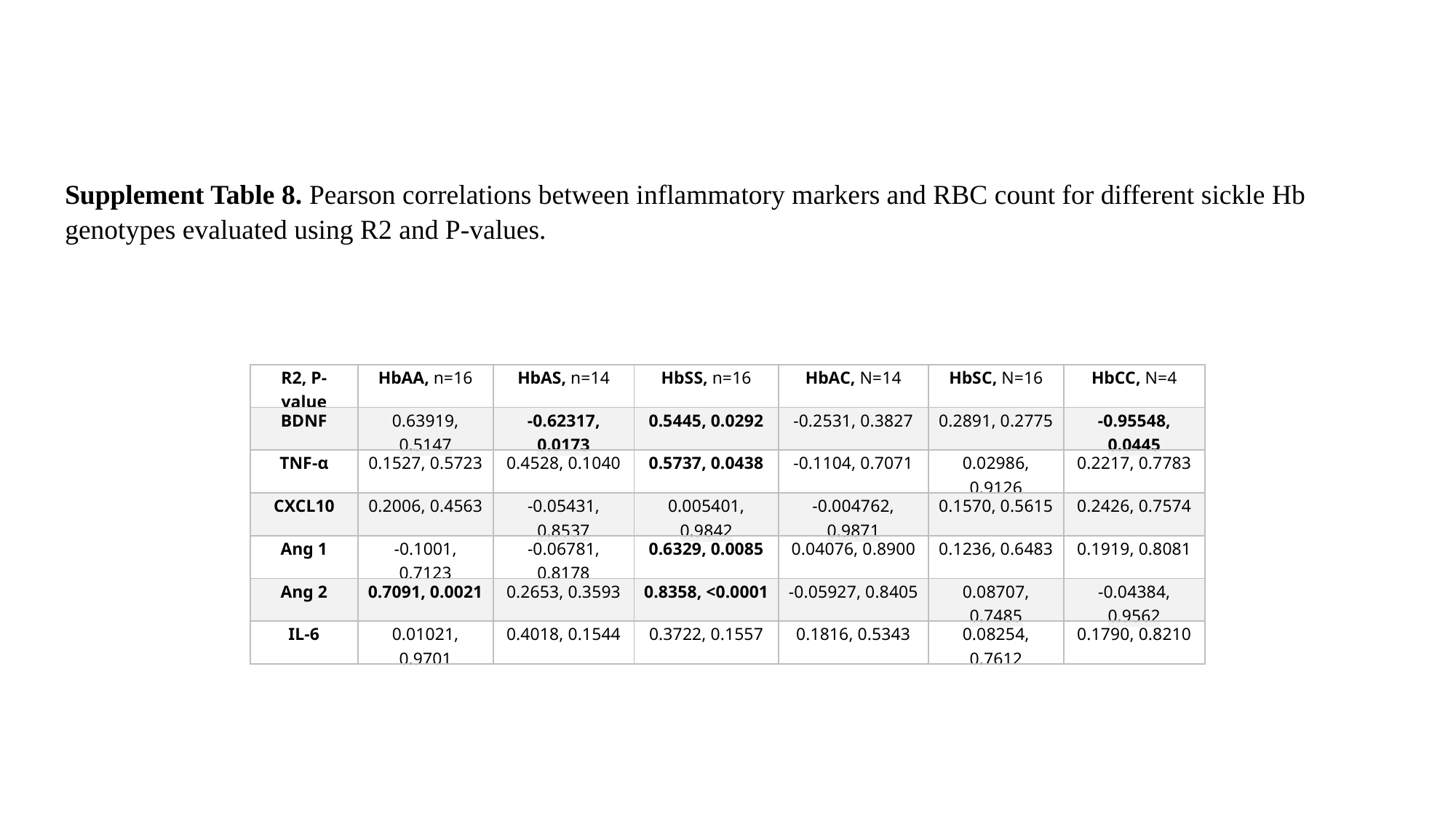

Supplement Table 8. Pearson correlations between inflammatory markers and RBC count for different sickle Hb genotypes evaluated using R2 and P-values.
| R2, P- value | HbAA, n=16 | HbAS, n=14 | HbSS, n=16 | HbAC, N=14 | HbSC, N=16 | HbCC, N=4 |
| --- | --- | --- | --- | --- | --- | --- |
| BDNF | 0.63919, 0.5147 | -0.62317, 0.0173 | 0.5445, 0.0292 | -0.2531, 0.3827 | 0.2891, 0.2775 | -0.95548, 0.0445 |
| TNF-α | 0.1527, 0.5723 | 0.4528, 0.1040 | 0.5737, 0.0438 | -0.1104, 0.7071 | 0.02986, 0.9126 | 0.2217, 0.7783 |
| CXCL10 | 0.2006, 0.4563 | -0.05431, 0.8537 | 0.005401, 0.9842 | -0.004762, 0.9871 | 0.1570, 0.5615 | 0.2426, 0.7574 |
| Ang 1 | -0.1001, 0.7123 | -0.06781, 0.8178 | 0.6329, 0.0085 | 0.04076, 0.8900 | 0.1236, 0.6483 | 0.1919, 0.8081 |
| Ang 2 | 0.7091, 0.0021 | 0.2653, 0.3593 | 0.8358, <0.0001 | -0.05927, 0.8405 | 0.08707, 0.7485 | -0.04384, 0.9562 |
| IL-6 | 0.01021, 0.9701 | 0.4018, 0.1544 | 0.3722, 0.1557 | 0.1816, 0.5343 | 0.08254, 0.7612 | 0.1790, 0.8210 |

## Slide 10
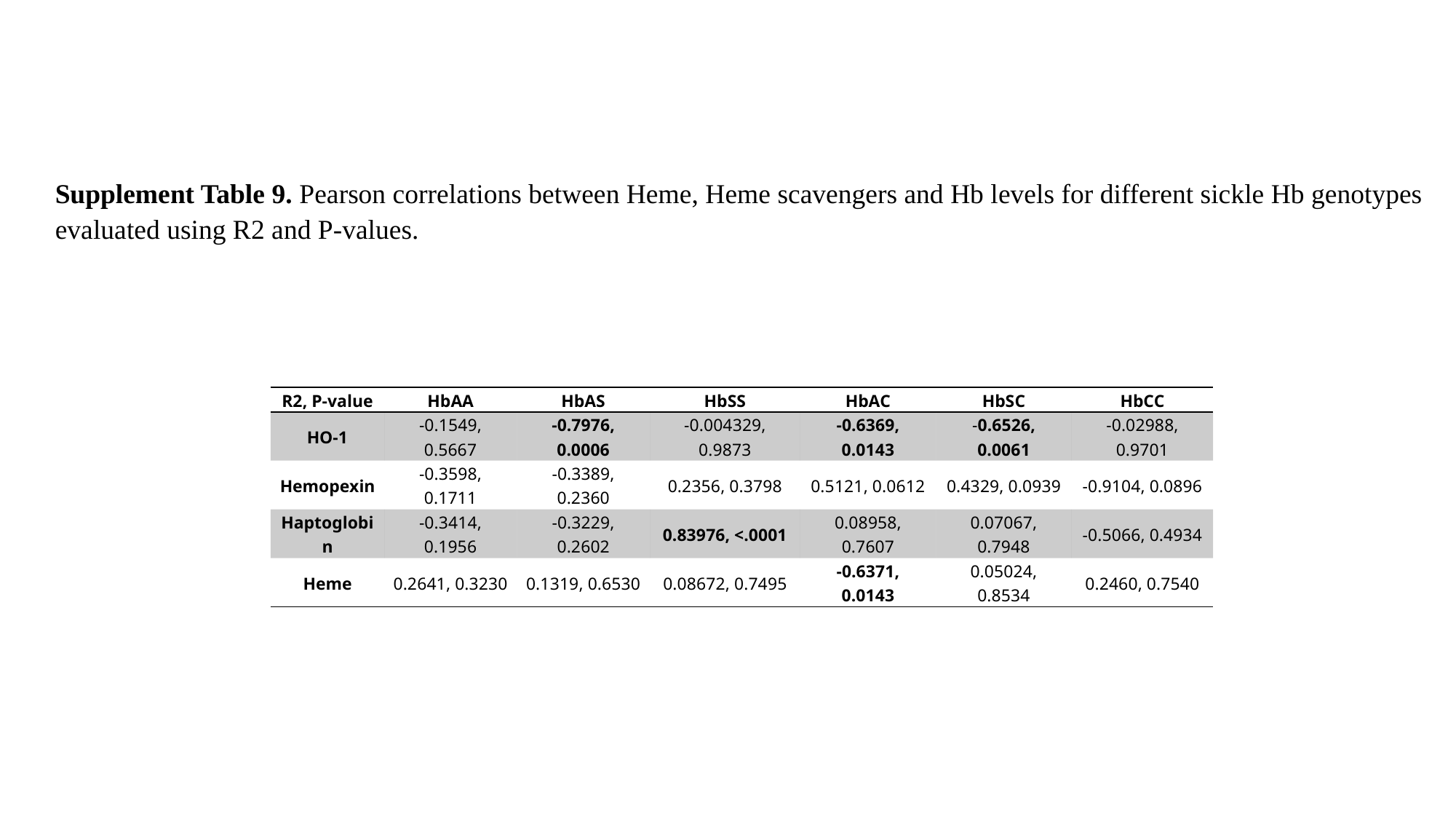

Supplement Table 9. Pearson correlations between Heme, Heme scavengers and Hb levels for different sickle Hb genotypes evaluated using R2 and P-values.
| R2, P-value | HbAA | HbAS | HbSS | HbAC | HbSC | HbCC |
| --- | --- | --- | --- | --- | --- | --- |
| HO-1 | -0.1549, 0.5667 | -0.7976, 0.0006 | -0.004329, 0.9873 | -0.6369, 0.0143 | -0.6526, 0.0061 | -0.02988, 0.9701 |
| Hemopexin | -0.3598, 0.1711 | -0.3389, 0.2360 | 0.2356, 0.3798 | 0.5121, 0.0612 | 0.4329, 0.0939 | -0.9104, 0.0896 |
| Haptoglobin | -0.3414, 0.1956 | -0.3229, 0.2602 | 0.83976, <.0001 | 0.08958, 0.7607 | 0.07067, 0.7948 | -0.5066, 0.4934 |
| Heme | 0.2641, 0.3230 | 0.1319, 0.6530 | 0.08672, 0.7495 | -0.6371, 0.0143 | 0.05024, 0.8534 | 0.2460, 0.7540 |

## Slide 11
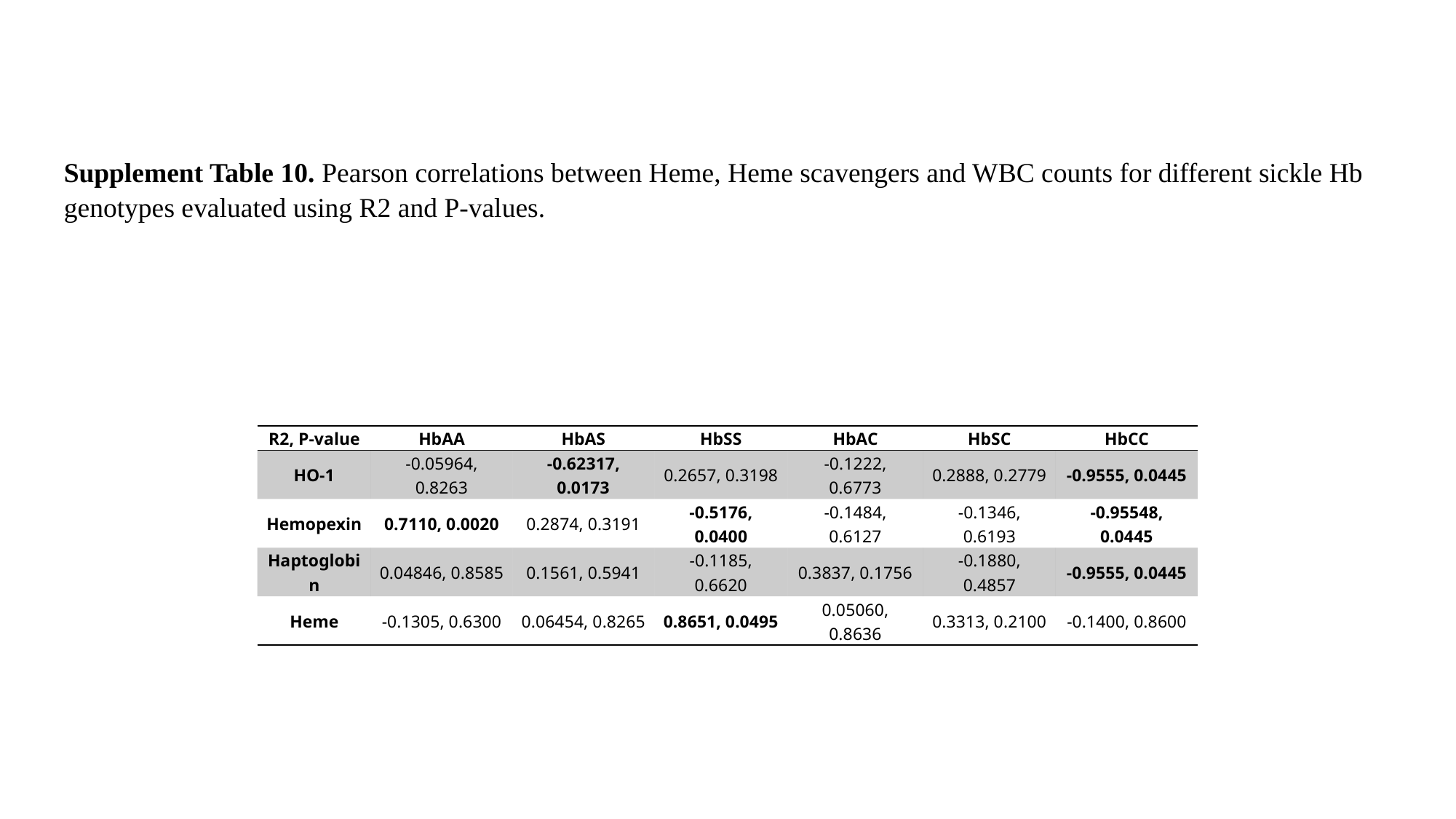

Supplement Table 10. Pearson correlations between Heme, Heme scavengers and WBC counts for different sickle Hb genotypes evaluated using R2 and P-values.
| R2, P-value | HbAA | HbAS | HbSS | HbAC | HbSC | HbCC |
| --- | --- | --- | --- | --- | --- | --- |
| HO-1 | -0.05964, 0.8263 | -0.62317, 0.0173 | 0.2657, 0.3198 | -0.1222, 0.6773 | 0.2888, 0.2779 | -0.9555, 0.0445 |
| Hemopexin | 0.7110, 0.0020 | 0.2874, 0.3191 | -0.5176, 0.0400 | -0.1484, 0.6127 | -0.1346, 0.6193 | -0.95548, 0.0445 |
| Haptoglobin | 0.04846, 0.8585 | 0.1561, 0.5941 | -0.1185, 0.6620 | 0.3837, 0.1756 | -0.1880, 0.4857 | -0.9555, 0.0445 |
| Heme | -0.1305, 0.6300 | 0.06454, 0.8265 | 0.8651, 0.0495 | 0.05060, 0.8636 | 0.3313, 0.2100 | -0.1400, 0.8600 |

## Slide 12
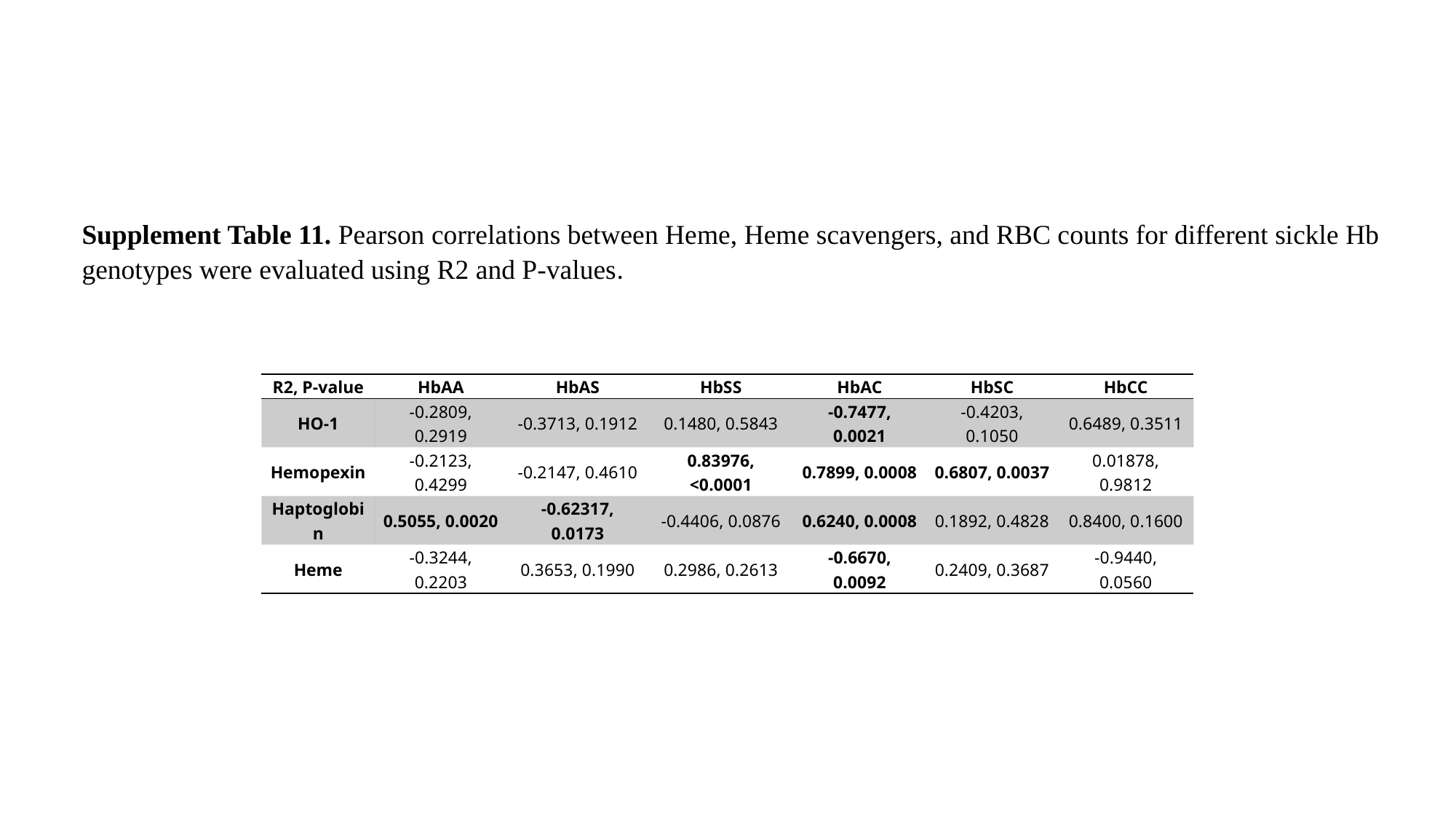

Supplement Table 11. Pearson correlations between Heme, Heme scavengers, and RBC counts for different sickle Hb genotypes were evaluated using R2 and P-values.
| R2, P-value | HbAA | HbAS | HbSS | HbAC | HbSC | HbCC |
| --- | --- | --- | --- | --- | --- | --- |
| HO-1 | -0.2809, 0.2919 | -0.3713, 0.1912 | 0.1480, 0.5843 | -0.7477, 0.0021 | -0.4203, 0.1050 | 0.6489, 0.3511 |
| Hemopexin | -0.2123, 0.4299 | -0.2147, 0.4610 | 0.83976, <0.0001 | 0.7899, 0.0008 | 0.6807, 0.0037 | 0.01878, 0.9812 |
| Haptoglobin | 0.5055, 0.0020 | -0.62317, 0.0173 | -0.4406, 0.0876 | 0.6240, 0.0008 | 0.1892, 0.4828 | 0.8400, 0.1600 |
| Heme | -0.3244, 0.2203 | 0.3653, 0.1990 | 0.2986, 0.2613 | -0.6670, 0.0092 | 0.2409, 0.3687 | -0.9440, 0.0560 |

## Slide 13
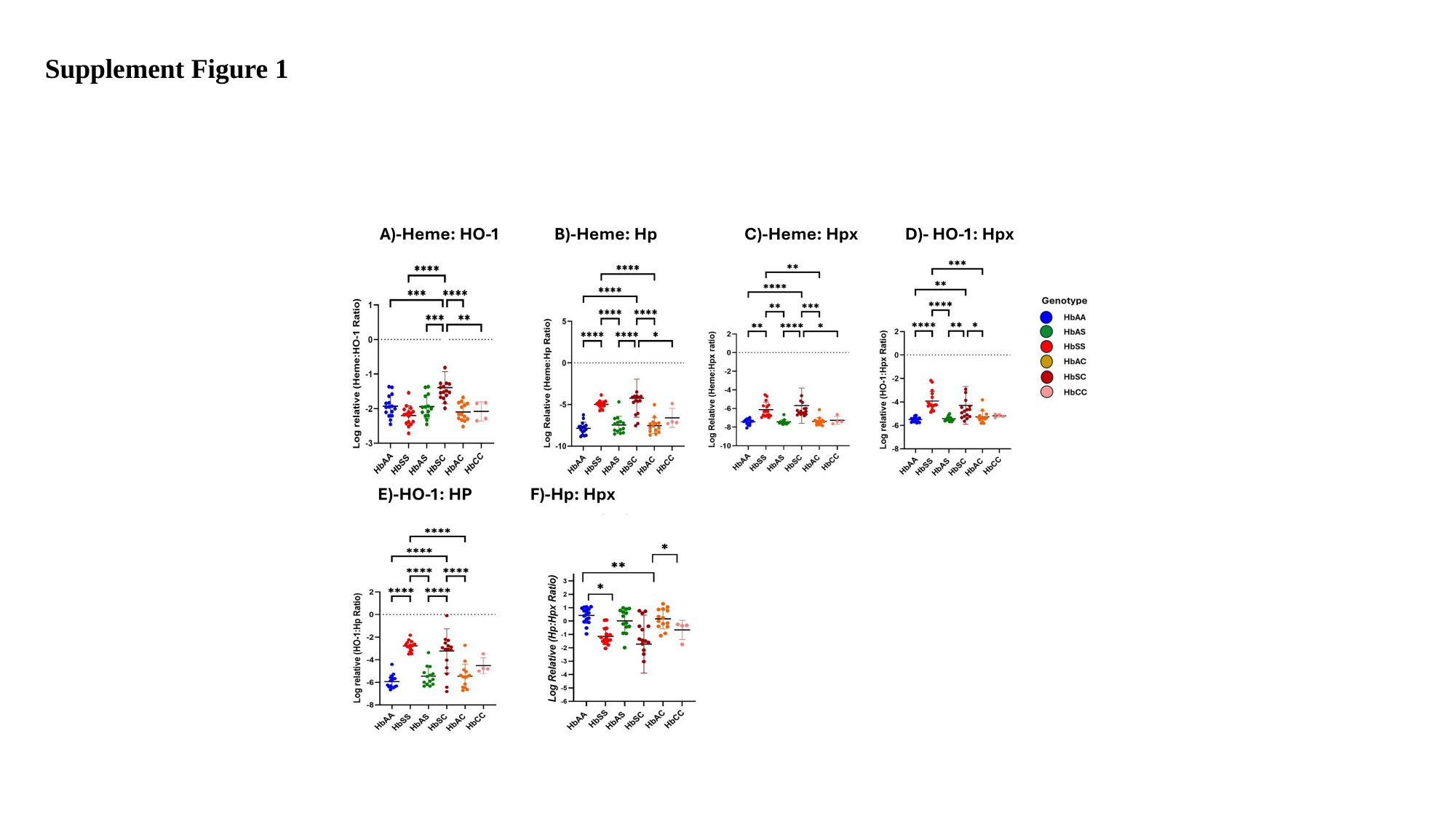

Supplement Figure 1

## Slide 14
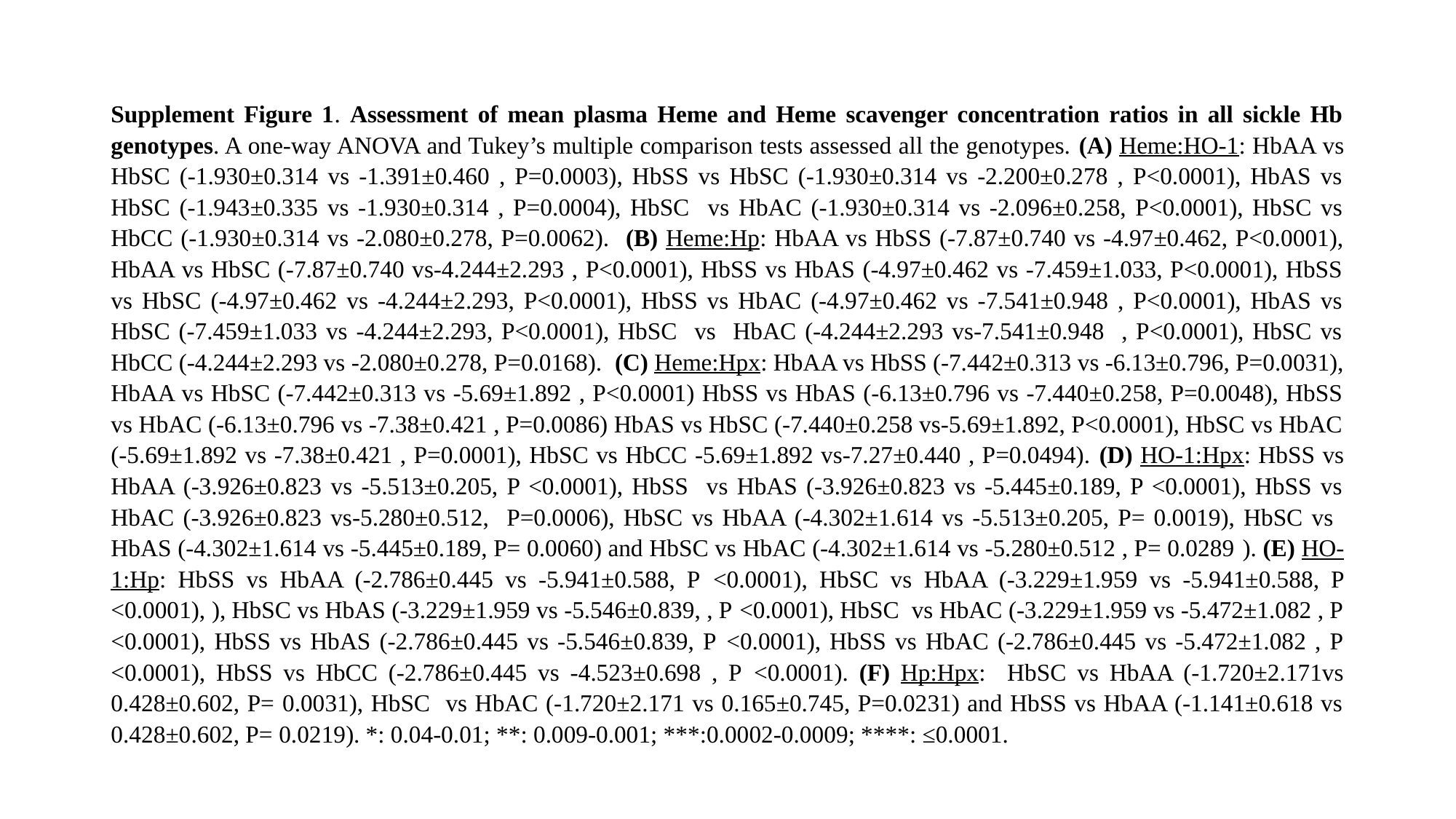

Supplement Figure 1. Assessment of mean plasma Heme and Heme scavenger concentration ratios in all sickle Hb genotypes. A one-way ANOVA and Tukey’s multiple comparison tests assessed all the genotypes. (A) Heme:HO-1: HbAA vs HbSC (-1.930±0.314 vs -1.391±0.460 , P=0.0003), HbSS vs HbSC (-1.930±0.314 vs -2.200±0.278 , P<0.0001), HbAS vs HbSC (-1.943±0.335 vs -1.930±0.314 , P=0.0004), HbSC vs HbAC (-1.930±0.314 vs -2.096±0.258, P<0.0001), HbSC vs HbCC (-1.930±0.314 vs -2.080±0.278, P=0.0062). (B) Heme:Hp: HbAA vs HbSS (-7.87±0.740 vs -4.97±0.462, P<0.0001), HbAA vs HbSC (-7.87±0.740 vs-4.244±2.293 , P<0.0001), HbSS vs HbAS (-4.97±0.462 vs -7.459±1.033, P<0.0001), HbSS vs HbSC (-4.97±0.462 vs -4.244±2.293, P<0.0001), HbSS vs HbAC (-4.97±0.462 vs -7.541±0.948 , P<0.0001), HbAS vs HbSC (-7.459±1.033 vs -4.244±2.293, P<0.0001), HbSC vs HbAC (-4.244±2.293 vs-7.541±0.948 , P<0.0001), HbSC vs HbCC (-4.244±2.293 vs -2.080±0.278, P=0.0168). (C) Heme:Hpx: HbAA vs HbSS (-7.442±0.313 vs -6.13±0.796, P=0.0031), HbAA vs HbSC (-7.442±0.313 vs -5.69±1.892 , P<0.0001) HbSS vs HbAS (-6.13±0.796 vs -7.440±0.258, P=0.0048), HbSS vs HbAC (-6.13±0.796 vs -7.38±0.421 , P=0.0086) HbAS vs HbSC (-7.440±0.258 vs-5.69±1.892, P<0.0001), HbSC vs HbAC (-5.69±1.892 vs -7.38±0.421 , P=0.0001), HbSC vs HbCC -5.69±1.892 vs-7.27±0.440 , P=0.0494). (D) HO-1:Hpx: HbSS vs HbAA (-3.926±0.823 vs -5.513±0.205, P <0.0001), HbSS vs HbAS (-3.926±0.823 vs -5.445±0.189, P <0.0001), HbSS vs HbAC (-3.926±0.823 vs-5.280±0.512, P=0.0006), HbSC vs HbAA (-4.302±1.614 vs -5.513±0.205, P= 0.0019), HbSC vs HbAS (-4.302±1.614 vs -5.445±0.189, P= 0.0060) and HbSC vs HbAC (-4.302±1.614 vs -5.280±0.512 , P= 0.0289 ). (E) HO-1:Hp: HbSS vs HbAA (-2.786±0.445 vs -5.941±0.588, P <0.0001), HbSC vs HbAA (-3.229±1.959 vs -5.941±0.588, P <0.0001), ), HbSC vs HbAS (-3.229±1.959 vs -5.546±0.839, , P <0.0001), HbSC vs HbAC (-3.229±1.959 vs -5.472±1.082 , P <0.0001), HbSS vs HbAS (-2.786±0.445 vs -5.546±0.839, P <0.0001), HbSS vs HbAC (-2.786±0.445 vs -5.472±1.082 , P <0.0001), HbSS vs HbCC (-2.786±0.445 vs -4.523±0.698 , P <0.0001). (F) Hp:Hpx: HbSC vs HbAA (-1.720±2.171vs 0.428±0.602, P= 0.0031), HbSC vs HbAC (-1.720±2.171 vs 0.165±0.745, P=0.0231) and HbSS vs HbAA (-1.141±0.618 vs 0.428±0.602, P= 0.0219). *: 0.04-0.01; **: 0.009-0.001; ***:0.0002-0.0009; ****: ≤0.0001.

## Slide 15
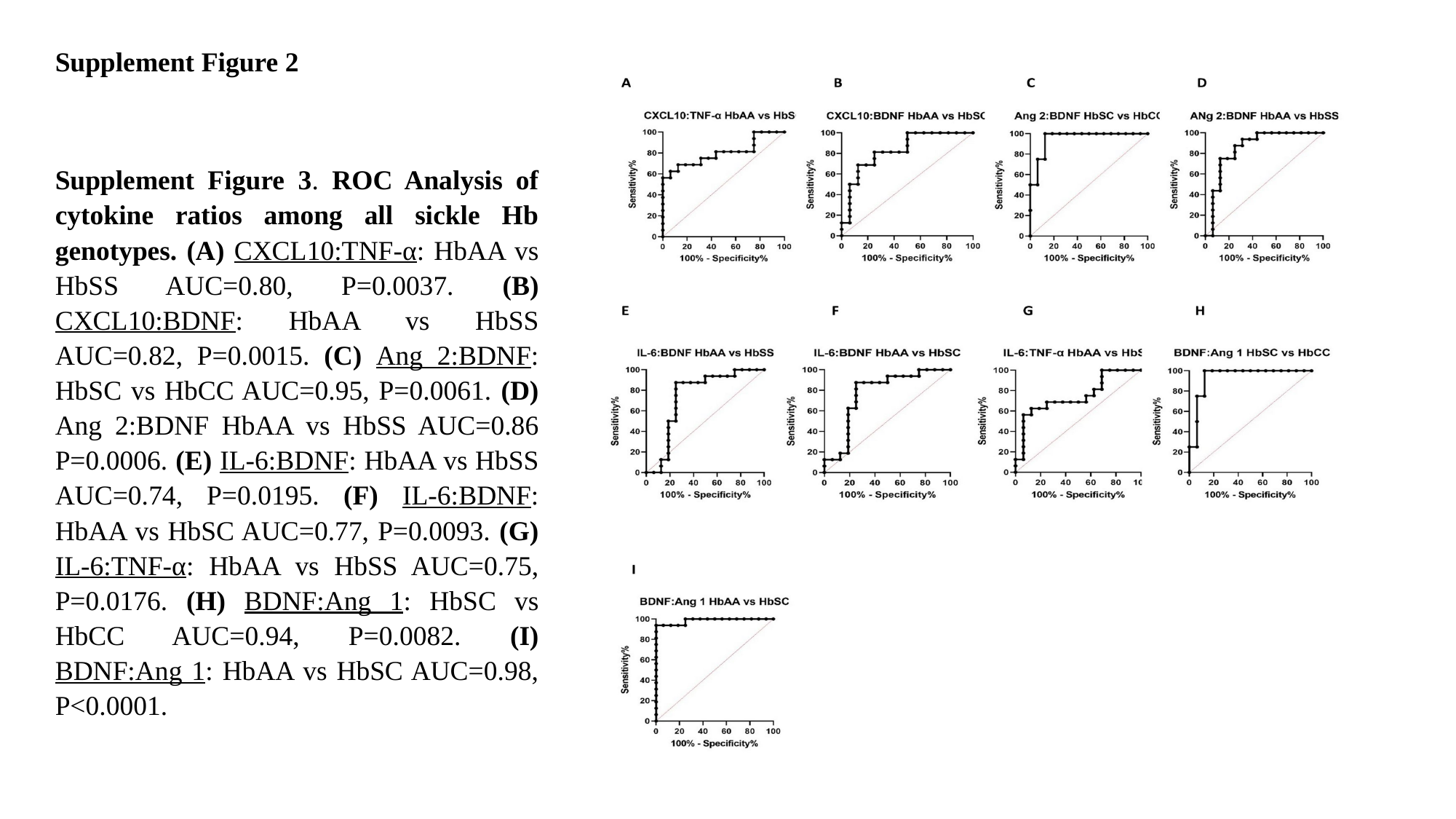

Supplement Figure 2
Supplement Figure 3. ROC Analysis of cytokine ratios among all sickle Hb genotypes. (A) CXCL10:TNF-α: HbAA vs HbSS AUC=0.80, P=0.0037. (B) CXCL10:BDNF: HbAA vs HbSS AUC=0.82, P=0.0015. (C) Ang 2:BDNF: HbSC vs HbCC AUC=0.95, P=0.0061. (D) Ang 2:BDNF HbAA vs HbSS AUC=0.86 P=0.0006. (E) IL-6:BDNF: HbAA vs HbSS AUC=0.74, P=0.0195. (F) IL-6:BDNF: HbAA vs HbSC AUC=0.77, P=0.0093. (G) IL-6:TNF-α: HbAA vs HbSS AUC=0.75, P=0.0176. (H) BDNF:Ang 1: HbSC vs HbCC AUC=0.94, P=0.0082. (I) BDNF:Ang 1: HbAA vs HbSC AUC=0.98, P<0.0001.

## Slide 16
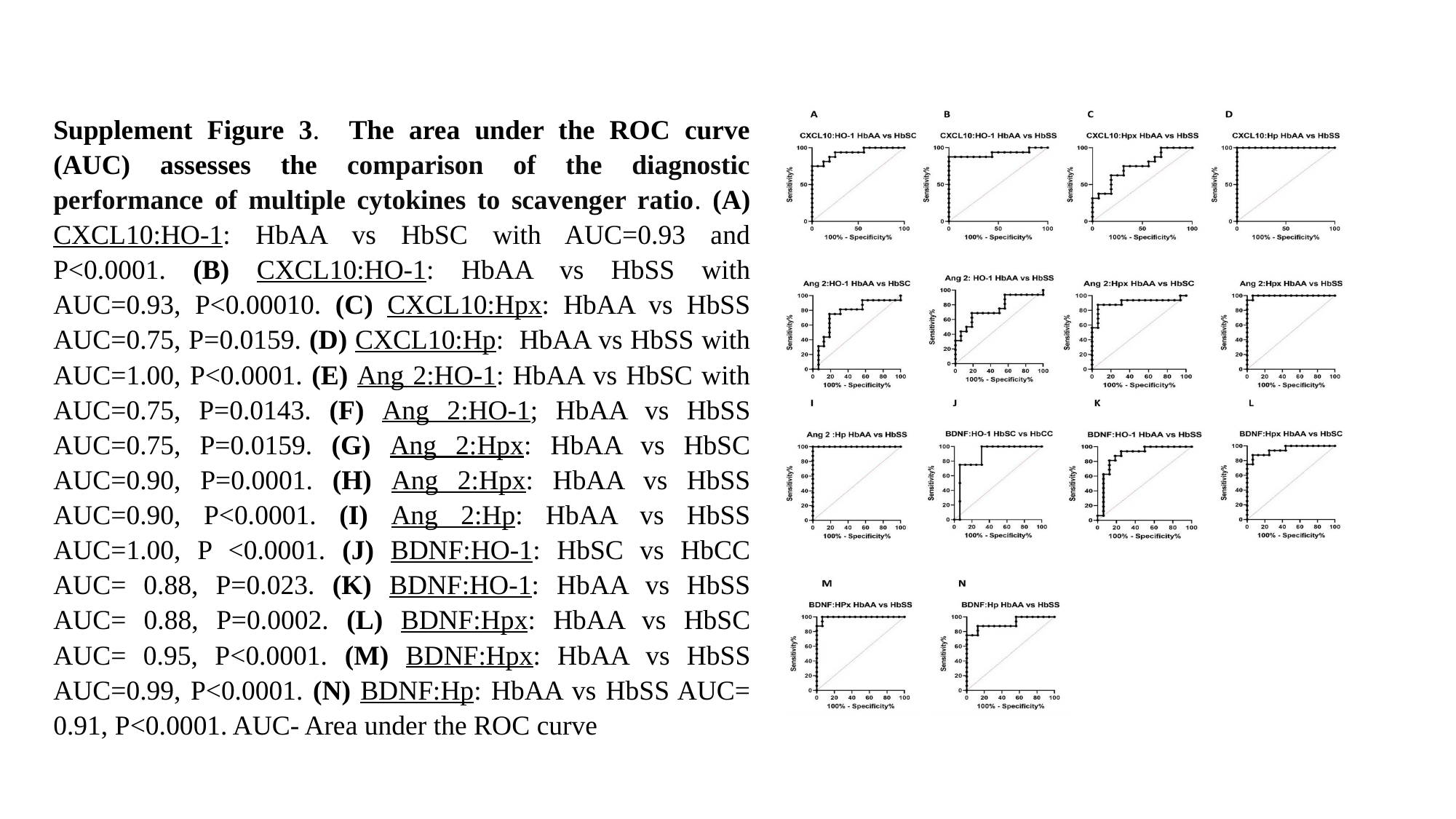

Supplement Figure 3. The area under the ROC curve (AUC) assesses the comparison of the diagnostic performance of multiple cytokines to scavenger ratio. (A) CXCL10:HO-1: HbAA vs HbSC with AUC=0.93 and P<0.0001. (B) CXCL10:HO-1: HbAA vs HbSS with AUC=0.93, P<0.00010. (C) CXCL10:Hpx: HbAA vs HbSS AUC=0.75, P=0.0159. (D) CXCL10:Hp: HbAA vs HbSS with AUC=1.00, P<0.0001. (E) Ang 2:HO-1: HbAA vs HbSC with AUC=0.75, P=0.0143. (F) Ang 2:HO-1; HbAA vs HbSS AUC=0.75, P=0.0159. (G) Ang 2:Hpx: HbAA vs HbSC AUC=0.90, P=0.0001. (H) Ang 2:Hpx: HbAA vs HbSS AUC=0.90, P<0.0001. (I) Ang 2:Hp: HbAA vs HbSS AUC=1.00, P <0.0001. (J) BDNF:HO-1: HbSC vs HbCC AUC= 0.88, P=0.023. (K) BDNF:HO-1: HbAA vs HbSS AUC= 0.88, P=0.0002. (L) BDNF:Hpx: HbAA vs HbSC AUC= 0.95, P<0.0001. (M) BDNF:Hpx: HbAA vs HbSS AUC=0.99, P<0.0001. (N) BDNF:Hp: HbAA vs HbSS AUC= 0.91, P<0.0001. AUC- Area under the ROC curve

## Slide 17
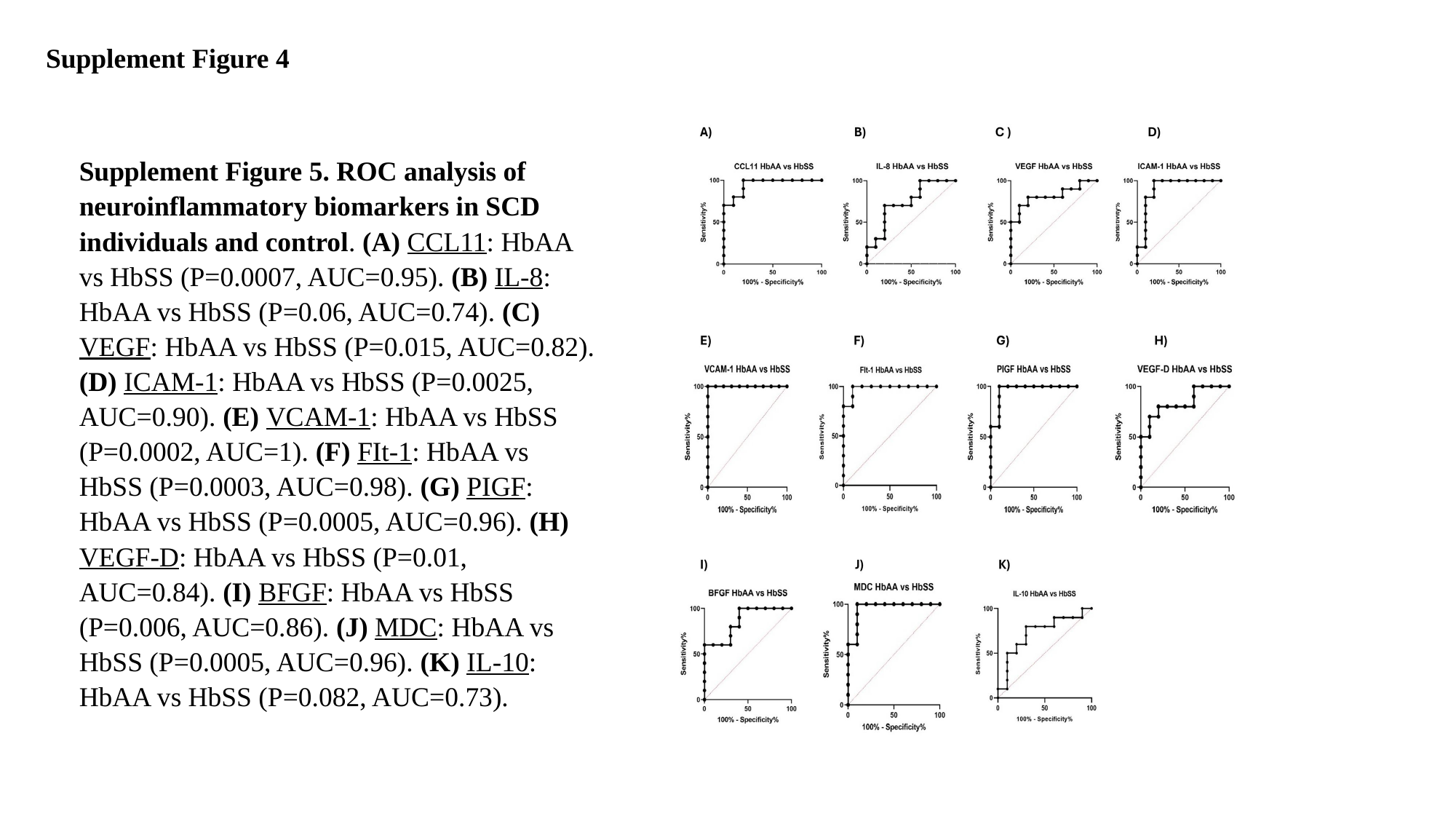

Supplement Figure 4
Supplement Figure 5. ROC analysis of neuroinflammatory biomarkers in SCD individuals and control. (A) CCL11: HbAA vs HbSS (P=0.0007, AUC=0.95). (B) IL-8: HbAA vs HbSS (P=0.06, AUC=0.74). (C) VEGF: HbAA vs HbSS (P=0.015, AUC=0.82). (D) ICAM-1: HbAA vs HbSS (P=0.0025, AUC=0.90). (E) VCAM-1: HbAA vs HbSS (P=0.0002, AUC=1). (F) FIt-1: HbAA vs HbSS (P=0.0003, AUC=0.98). (G) PIGF: HbAA vs HbSS (P=0.0005, AUC=0.96). (H) VEGF-D: HbAA vs HbSS (P=0.01, AUC=0.84). (I) BFGF: HbAA vs HbSS (P=0.006, AUC=0.86). (J) MDC: HbAA vs HbSS (P=0.0005, AUC=0.96). (K) IL-10: HbAA vs HbSS (P=0.082, AUC=0.73).

## Slide 18
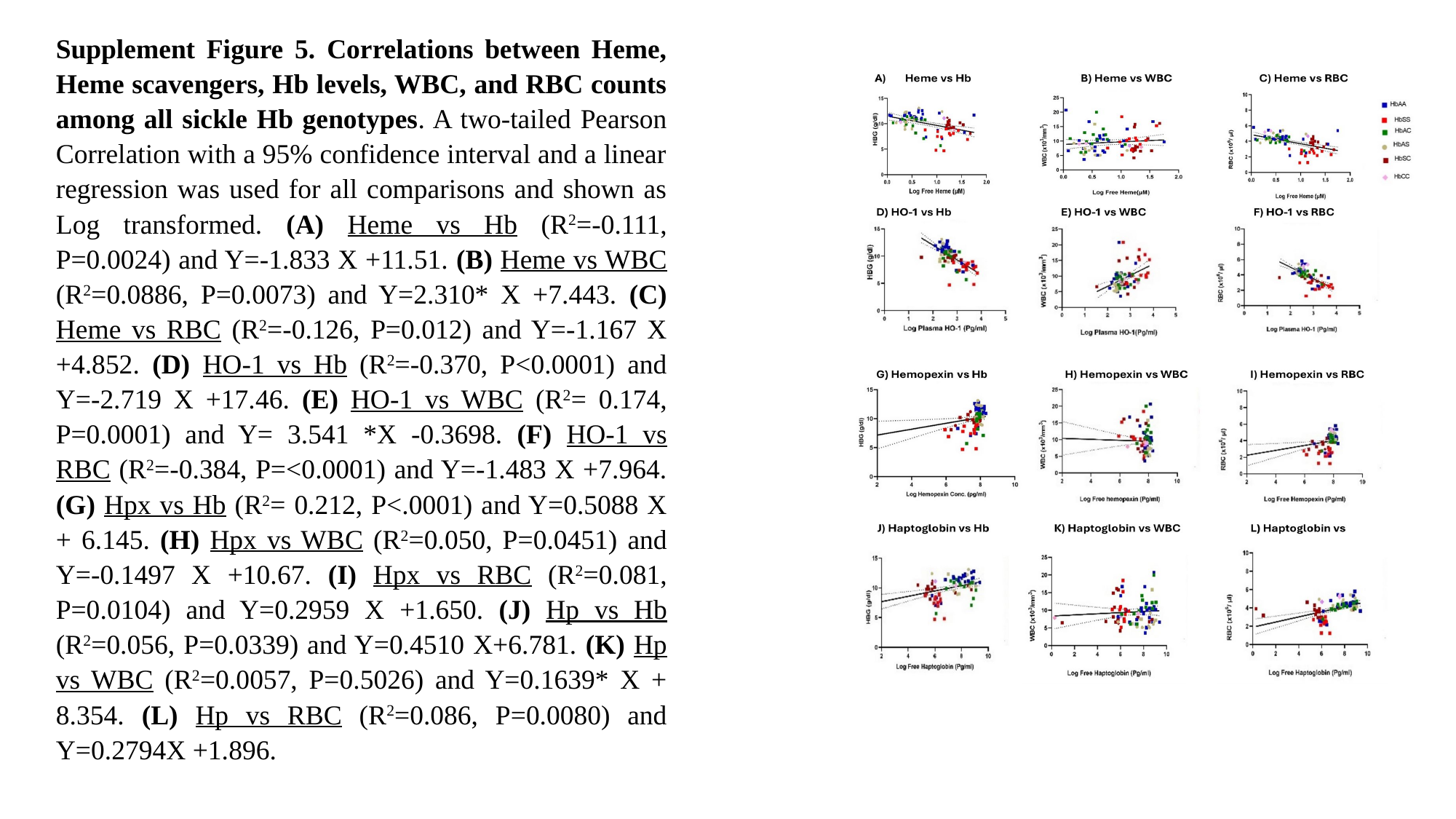

Supplement Figure 5. Correlations between Heme, Heme scavengers, Hb levels, WBC, and RBC counts among all sickle Hb genotypes. A two-tailed Pearson Correlation with a 95% confidence interval and a linear regression was used for all comparisons and shown as Log transformed. (A) Heme vs Hb (R2=-0.111, P=0.0024) and Y=-1.833 X +11.51. (B) Heme vs WBC (R2=0.0886, P=0.0073) and Y=2.310* X +7.443. (C) Heme vs RBC (R2=-0.126, P=0.012) and Y=-1.167 X +4.852. (D) HO-1 vs Hb (R2=-0.370, P<0.0001) and Y=-2.719 X +17.46. (E) HO-1 vs WBC (R2= 0.174, P=0.0001) and Y= 3.541 *X -0.3698. (F) HO-1 vs RBC (R2=-0.384, P=<0.0001) and Y=-1.483 X +7.964. (G) Hpx vs Hb (R2= 0.212, P<.0001) and Y=0.5088 X + 6.145. (H) Hpx vs WBC (R2=0.050, P=0.0451) and Y=-0.1497 X +10.67. (I) Hpx vs RBC (R2=0.081, P=0.0104) and Y=0.2959 X +1.650. (J) Hp vs Hb (R2=0.056, P=0.0339) and Y=0.4510 X+6.781. (K) Hp vs WBC (R2=0.0057, P=0.5026) and Y=0.1639* X + 8.354. (L) Hp vs RBC (R2=0.086, P=0.0080) and Y=0.2794X +1.896.

## Slide 19
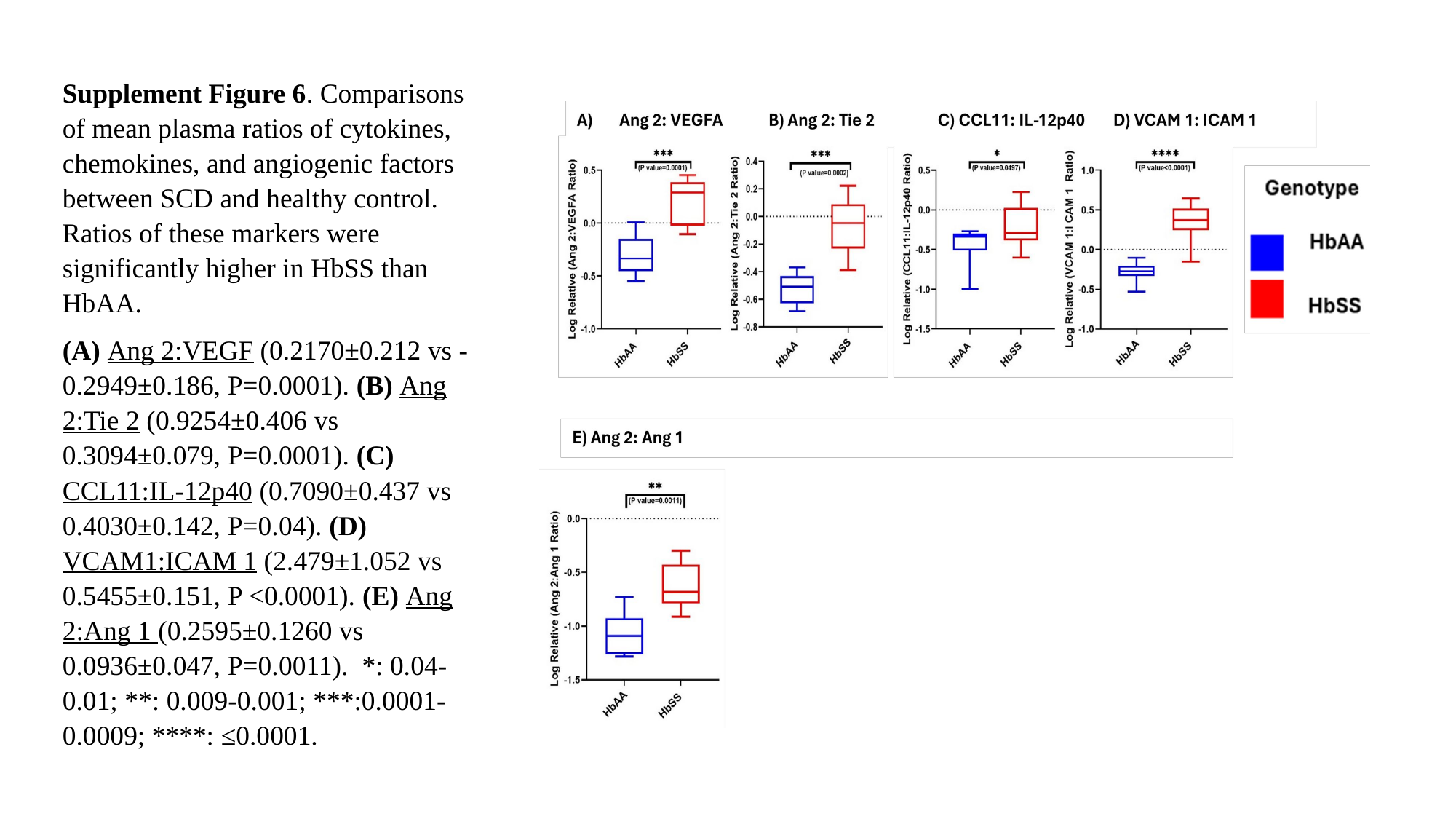

Supplement Figure 6. Comparisons of mean plasma ratios of cytokines, chemokines, and angiogenic factors between SCD and healthy control. Ratios of these markers were significantly higher in HbSS than HbAA.
(A) Ang 2:VEGF (0.2170±0.212 vs -0.2949±0.186, P=0.0001). (B) Ang 2:Tie 2 (0.9254±0.406 vs 0.3094±0.079, P=0.0001). (C) CCL11:IL-12p40 (0.7090±0.437 vs 0.4030±0.142, P=0.04). (D) VCAM1:ICAM 1 (2.479±1.052 vs 0.5455±0.151, P <0.0001). (E) Ang 2:Ang 1 (0.2595±0.1260 vs 0.0936±0.047, P=0.0011). *: 0.04-0.01; **: 0.009-0.001; ***:0.0001-0.0009; ****: ≤0.0001.
